# Race-Specific Genetic Profiles of Homologous Recombination Deficiency in Multiple Cancers

**DOI:** 10.3390/jpm11121287

**Published:** 2021-12-03

**Authors:** Yi-Wen Hsiao, Tzu-Pin Lu

**Affiliations:** 1 Institute of Epidemiology and Preventive Medicine, College of Public Health, National Taiwan University, Taipei 100, Taiwan; d08849010@ntu.edu.tw; 2 Bioinformatics and Biostatistics Core, Center of Genomic and Precision Medicine, National Taiwan University, Taipei 100, Taiwan

**Keywords:** homologous recombination deficiency, mutation, structural variation, pan-cancer, racial difference, therapeutic targets

## Abstract

Homologous recombination deficiency (HRD) has been used to predict both cancer prognosis and the response to DNA-damaging therapies in many cancer types. HRD has diverse manifestations in different cancers and even in different populations. Many screening strategies have been designed for detecting the sensitivity of a patient’s HRD status to targeted therapies. However, these approaches suffer from low sensitivity, and are not specific to each cancer type and population group. Therefore, identifying race-specific and targetable HRD-related genes is of clinical importance. Here, we conducted analyses using genomic sequencing data that was generated by the Pan-Cancer Atlas. Collapsing non-synonymous variants with functional damage to HRD-related genes, we analyzed the association between these genes and race within cancer types using the optimal sequencing kernel association test (SKAT-O). We have identified race-specific mutational patterns of curated HRD-related genes across cancers. Overall, more significant mutation sites were found in *ATM*, *BRCA2*, *POLE*, and *TOP2B* in both the ‘White’ and ‘Asian’ populations, whereas *PTEN*, *EGFG*, and *RIF1* mutations were observed in both the ‘White’ and ‘African American/Black’ populations. Furthermore, supported by pathogenic tendency databases and previous reports, in the ‘African American/Black’ population, several associations, including *BLM* with breast invasive carcinoma, *ERCC5* with ovarian serous cystadenocarcinoma, as well as *PTEN* with stomach adenocarcinoma, were newly described here. Although several HRD-related genes are common across cancers, many of them were found to be specific to race. Further studies, using a larger cohort of diverse populations, are necessary to identify HRD-related genes that are specific to race, for guiding gene testing methods.

## 1. Introduction

Homologous recombination deficiency (HRD) is a dysfunction of the homologous recombination repair (HRR) pathway, which is responsible for the repair of DNA double-strand breaks [1]. HRR pathway defects are often caused by the mutation of *BRCA1* or *BRCA2* but they can also arise from mutations in HRR-related genes, such as *AMT* or *CDK12,* that carry different mutation loads [2]. Aside from genetic mutations, structural aberrations of DNA, such as a loss of heterozygosity (LOH), large-scale state transitions (LST), and telomeric allelic imbalance (TAI), have been recognized as defining characteristics of HRD [3,4,5]. These genetic features are associated with an increased sensitivity to DNA-damaging agents, such as poly ADP-ribose polymerase (PARP) inhibitors and platinum-based antitumor drugs [6]. For this reason, they have served as targets of chemotherapy and immunotherapy agents for several cancers, especially in breast cancer, ovarian cancer, pancreatic cancer, and prostate cancer [7,8,9,10]. In addition to their response to drugs, the survival outcomes of many cancer patients are also associated with the degree of HRD in many cancers [11]. Thus, the genetic features related to HRD are capable of being used as biomarkers to predict patients’ drug response and survival outcomes.

Several epidemiological studies have pointed to large racial differences in cancer incidence and survival of many cancer types [12,13]. According to data from US cancer registries, African American/Black (AA/B) people have a higher incidence and lower survival of all cancers when compared to the White population, after adjusting for confounding factors, such as socioeconomic status and behavior [14]. Some of this difference may be caused by non-genetic factors, but a considerable amount may be attributed to genomic architecture. Substantial evidence has shown that the population-specific genetic background can at least partially explain the reason for unequal cancer burden among different racial groups [15,16]. For instance, Conti et al. found that an African American/Black male has a 75% higher risk of getting prostate cancer than a White man, noting that past studies have overrepresented the White population and ignored this variation in cancer risk by race [17]. Recent research has also revealed that there is a higher prevalence of HRD events in lung cancer occurring in African Americans than in European Americans, highlighting the necessity of including underrepresented populations in genetic studies [18]. Therefore, bringing the genetic risk for people of various racial groups into focus may help to explain the role of race in the progression of cancer and HRD events, leading to better screening protocols in people of all races, as well as the earlier detection of each type of cancer.

The fundamental goal of precision medicine in cancer care is to use genetic information to prevent, diagnose, or treat cancers, and advances in this area have led to the development of targeted cancer therapies [19]. Several genetic testing strategies are available for many cancers, especially breast cancer, which is the most well-studied cancer type. For example, the Oncotype DX Breast Recurrence Score test, which is based on 21 genes [20], and the MammaPrint test, which is based on a 70-gene signature, can estimate the risk of recurrence [21], and have both been widely applied to guide the clinical treatment of breast cancer patients. Recently, an increasing number of tumor testing kits have been designed for HRD detection, such as Myriad myChoice^®^ CDx [22] and FoundationOne^®^ CDx [23]. Although current genetic testing still suffers from limited availability across cancer types, as well as false positive/negative issues, the role of such testing remains essential in clinical applications to achieve the ultimate goal of precision medicine.

Currently, several genetic testing methods, such as *BRCA1/2* germline testing alone, in a combination with HRR-related genes, and in a combination with genomic instability, have shown a wide range of sensitivity in HRD detection [24]. The best testing approach identifies only about 50% of women who are eligible for treatment with breast cancer drugs, such as a mixture of LYNPARZA and bevacizumab [25], highlighting that many patients are missed due to high false-negative results, such that the accurate detection of this population, using better biomarkers of HRD, is of clinical relevance. Although multiple factors underlie the racial differences in cancer prognosis and drug response, many population-based studies have pointed out that these differences may be partially attributed to inherent differences at the DNA level [26,27,28]. Therefore, the overarching aim of this study is to identify race-specific pathogenic HRD-related variants among cancers, providing new insight that is specific to each cancer type and population group.

## 2. Materials and Methods

### 2.1. Study Cohorts and Patients

Clinical and biospecimen annotation files in txt format (*n* = 9125, across 33 cancer types) were downloaded from the TCGA Pan-Cancer Clinical Data Resource [29]. A full list of TCGA cancer type abbreviations can be found in the Genomic Data Commons (https://gdc.cancer.gov/resources-tcga-users/tcga-code-tables/tcga-study-abbreviations (accessed on 2 June 2021)). Samples that did not belong to one of the three racial groups according to the TCGA Pan-Cancer Clinical Data—‘White’, ‘Asian’, and ‘African American/Black’—or did not have survival information were excluded. To ensure we had sufficient sample sizes to achieve adequate power in each cohort, only the cancer cohorts with >100 samples were included, and racial subgroups within each cancer cohort that had <10 samples were excluded. Consequently, 7241 patients across 24 cancer types were retained and combined (i.e., the pan-cancer cohort) for the downstream analysis. All sample selections were conducted using R software (v4.0.5).

### 2.2. The Determination of Pathogenicity for Somatic Variant Calls

A mutation annotation file (MAF; mc3.v0.2.8.PUBLIC.maf), including 33 cancer types, which was generated by the MuTect2 pipeline according to the GRCh38.d1.vd1 reference sequence, was also retrieved through the Pan-Cancer Atlas (https://gdc.cancer.gov/about-data/publications/pancanatlas (accessed on 2 June 2021)). To define the causal HRD genes, gene lists from a survey of the literature were collected and named as ‘DDRD_assay_42 [30], ‘mutated_gene_21’ [1], ‘HR_PARP_132’ [1], and ‘DDR_276’ [31]. The approximate chromosomal position of each gene in the lists, based on the GRCh38 genome version, was extracted from GeneCards (https://www.genecards.org/ (accessed on 2 June 2021)) by an in-house python web scraping script (https://github.com/ywhsiao/jpm_submission (accessed on 2 June 2021)). Through such information, the somatic variants in the MAF file within the region of these genes were then extracted. A measurement of global mutation loads, called tumor mutation burden (TMB), was calculated by directly counting the number of variants and then dividing that number by the genomic length of the target gene (unit: mutations/Mb). The above-mentioned filtering and calculation steps were performed by R software (v4.0.5).

### 2.3. Groupwise Association Test, Outlier Detection Analysis, and Variant Annotation

The previously downloaded MAF was modified and combined with the racial information from the previously downloaded clinical data for the groupwise association test. The optimal sequencing kernel association test (SKAT-O), which is a linear combination of the burden test and SKAT statistics [32], was performed to evaluate the association between the missense variants with a functional impact, which were classified as “probably/possibly damaging” or “deleterious” in two functional prediction algorithms (SIFT and PolyPhen-2), and a phenotype (i.e., a specific race) using the SKAT R package. In addition, a principal component analysis of all variants was conducted by PLINK (v2.0), and the resulting eigenvectors were used as a covariate to control the regression-based analysis. Then, we applied the rare variant influential filtering tool (RIFT), an R package which generates a delta chi-square score for each significant variant, and non-parametric outlier detection methods to identify the most influential variants that were specific to race [33]. Finally, we used pathogenicity determinations by REVEL and ClinVar to validate the pathogenic tendency of the identified variants [34,35].

### 2.4. HRD Score Calculation

The copy number segmentation file with annotations (TCGA_mastercalls.abs_segtabs.fixed.txt) and the purity/ploidy file (TCGA_mastercalls.abs_tables_JSedit.fixed.txt) that was generated by ABSOLUTE software were downloaded from the above-mentioned Pan-Cancer Atlas portal. These two files were then compiled to generate the input format supported by the scarHRD R package [36] for the calculation of the counts of each HRD component (HRD-loss of heterozygosity (HRD-LOH), HRD-large-scale state transitions (HRD-LST), and HRD-telomeric allelic imbalance (HRD-TAI)) and then summarized the total HRD score. Here, we evaluated the global and geneset-specific HRD scores based on previously reported genesets across cancers.

### 2.5. Statistical Analysis

To further compare the HRD scores and TMB values across race in each specific cancer cohort, the Wilcoxon rank-sum test or the Kruskal-Wallis test were used. When comparing such values among the three races, the false discovery rate was used for the correlation of multiple comparisons. A *p*-value of less than 0.05 is reported as statistically significant. Spearman’s correlation coefficient was calculated to evaluate the correlations among the genome-wide HRD scores and global TMB values across cancers. Statistical analyses and data visualizations were performed using the ‘ggpubr’ [37] and ‘ggsignif’ [38] R packages.

### 2.6. Survival Analysis

To investigate the effect of genomic features on survival, univariate or multivariate covariate-adjusted Cox proportional hazards models were used when the following covariates were provided in the sample’s clinical information: gender, age at initial pathogenic diagnosis, TNM (tumor, nodes, and metastases) status, and stage. The performance of the survival predictors was assessed by the concordance index (c-index). The hazard ratios (HRs), along with their 95% confidence intervals (CIs) and the corresponding *p*-values of these predictors, were calculated. We also dichotomized the patients according to the second tertile value (66.7%) of the HRD score to create two groups (‘HRD’ versus ‘not HRD’) [39]. This allowed the group with the higher level of HRD, defined as HRD in this study, to always possess one-third of the patients in each dataset. The Kaplan-Meier method was used to estimate the survival endpoints and assess the significant differences in the survival outcomes between the two predefined groups through the log-rank test. The above-mentioned statistical tests were conducted by the ‘ggpubr’, ‘survival’ [40], and ‘survminer’ [41] R packages.

## 3. Results

### 3.1. Distribution of the TCGA Pan-Cancer Atlas Cohort across Racial Groups

We summarize the distribution of the 7241 TGCA cases with their HRD scores across the three racial groups in Table 1. The ‘White’ group contained 83.04% (*n* = 6013) of the cohort, and the rest of the cohort consisted of 10.04% (*n* = 727) African American/Black and 6.92% (*n* = 501) Asian patients. Among the 24 cancer types, the largest population-specific cancer cohorts were breast invasive carcinoma (BRCA) for the ‘White’ group (*n* = 678) and the African American/Black group (*n* = 159), and liver hepatocellular carcinoma (LIHC) for the ‘Asian’ group (*n* = 150).

### 3.2. Association of HRD Scores with Survival across Cancer Types

**Figure 1:**
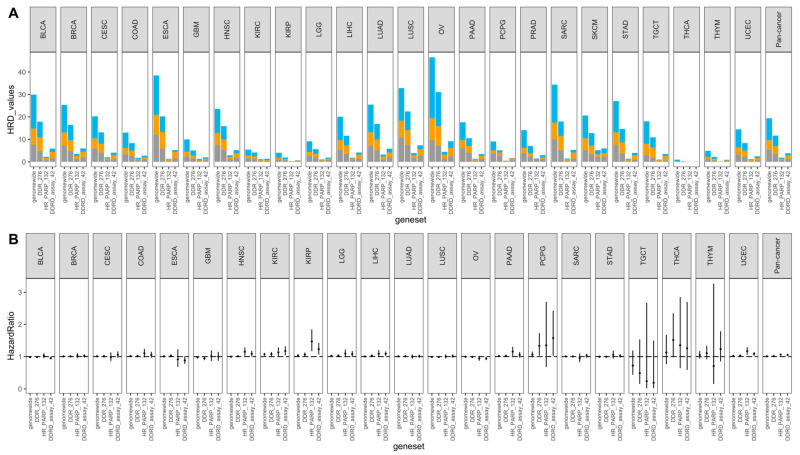
The distribution of HRD scores defined by different genesets were varied in relation to their prognostic prediction. (**A**) Stacked bar plot for the distribution of HRD scores. Total scores were the sum of HRD-LOH (gray), HRD-TAI (orange), and HRD-LST (blue) across the different genesets for 24 individual cancer types and pan-cancer. (**B**) Forest plots of the association between the HRD score and overall survival. Results are shown for 24 cancer types and pan-cancer with valid outcomes data. Hazard ratios and 95% confidence intervals are shown by the dot and the line, respectively. HRD: homologous recombination deficiency; LOH: loss of heterozygosity; TAI: telomeric allelic imbalance; LST: large-scale state transitions.

We first determined the HRD scores across the Pan-Cancer Atlas cancer types by integrating the data on copy number segment and ploidy, as defined by ABSOLUTE. The HRD scores are shown in Figure 1A. The HRD scores, which were defined by different genesets, varied by cancer type. We do knowledge that a geneset with a lower number of genes, or which did not have a proper gene list, had a lower HRD score estimated by the scarHRD R package. Therefore, we discarded the result defined by ‘mutated_gene_21’ here. The HRD distribution patterns of the genome-wide and ‘DDR_276’ genesets were similar, though the calculated HRD scores that were based on ‘DDR_276’ were generally lower than the genome-wide scores. For example, the cancer types which were ranked as having the top 5 highest HRD scores were the same in both genesets: ovarian serous cystadenocarcinoma (OV), esophageal carcinoma (ESCA), sarcoma (SARC), lung squamous cell carcinoma (LUSC), and bladder urothelial carcinoma (BLCA). Intriguingly, the HRD scores that were defined by ‘DDRD_assay_42’ were higher than those defined by ‘HR_PARP_132’ across the cancers analyzed.

**Figure 2:**
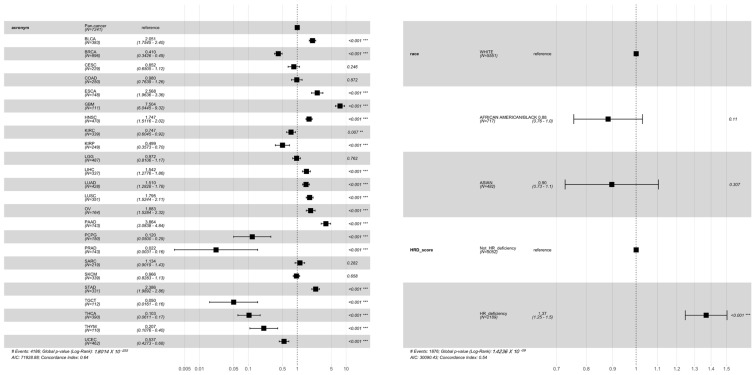
Forest plot of the association between variable clinical characteristics (cancer type, race, and dichotomized HRD scores) and survival. HRD scores were defined at the genome-wide level. The hazard ratios (dots) and their 95% confident intervals (lines) were estimated via a Cox proportional hazards analysis. “**”, “***” represents the statistical significance.

We next investigated the association of the HRD scores that were defined by the different genesets with overall survival. The ability of HRD scores to predict survival was also different across cancers and even across the defined genesets; some of the genesets (e.g., ‘HR_PARP_132’) even showed diverse prediction outcomes when compared with the rest of the genesets (Figure 1B). These results show that the HRD, defined by different genesets, may affect the incidence of HRD events and their corresponding clinical outcomes across cancers. For the risk prediction (Figure S1A,B), we only focused on four cancer types (BRCA, pancreatic adenocarcinoma (PAAD), prostate adenocarcinoma (PRAD), and OV) that are widely discussed in the field of HRD and PARP inhibitors, along with the pan-cancer results. In general, the HRD scores were not significantly linked to survival for the individual cancers, however, they were for the pan-cancer analysis. Of note, HRD, as defined here (top tertile), was associated with the survival of OV only when using genome-wide scores to define HRD (and in a counterintuitive direction), whereas survival was associated with the HRD score in the pan-cancer cohort under HRDs from any of the genesets. The relationship between the cancer types and survival, which was determined by the Cox proportional hazards analysis (Figure 2), further clarified that different cancer types had different survival outcomes, suggesting that the mortality risk, as defined by HRD scores, should take cancer type into consideration.

### 3.3. Synergistic Effects between Genome-Wide HRD and Global TMB among Cancers

It is known that both the quantity of DNA mutations (global TMB value) and the changes in the copy number (genome-wide HRD scores) can be used to identify patients with HRD [42,43]. To explore whether these two indicators were correlated, we calculated the Spearman correlation coefficients between genome-wide HRD scores and global TMB values, and the c-index was examined to evaluate their ability to predict overall survival. In Figure S2A, distinct correlation levels between HRD scores and TMB levels were observed in several cancer cohorts, as well as the pan-cancer cohort. These two genomic indicators were positively correlated (*p* < 0.001) in PAAD (*R* = 0.54), SARC (*R* = 0.5), and BRCA (*R* = 0.54), and negatively correlated in colon adenocarcinoma (COAD; *R* = −0.24, *p* < 0.00) and uterine corpus endometrial carcinoma (UCEC; *R* = −0.32, *p* < 0.001) (Figure S2A, Table 2). As shown in Figure S2B, the predictive values of these indicators were generally the same in most cancers, as well as in the pan-cancer analysis; however, the predictive ability of the HRD scores in some cancer cohorts (e.g., kidney renal papillary cell carcinoma (KIRP), PAAD, PRAD, and testicular germ cell tumors (TGCT)) outweighed that of the global TMB values. Nevertheless, these results still suggest that TMB may be representative of HRD events in some cancers.

### 3.4. Racial Differences of Genome-Wide HRD and Global TMB across Cancers

**Figure 3:**
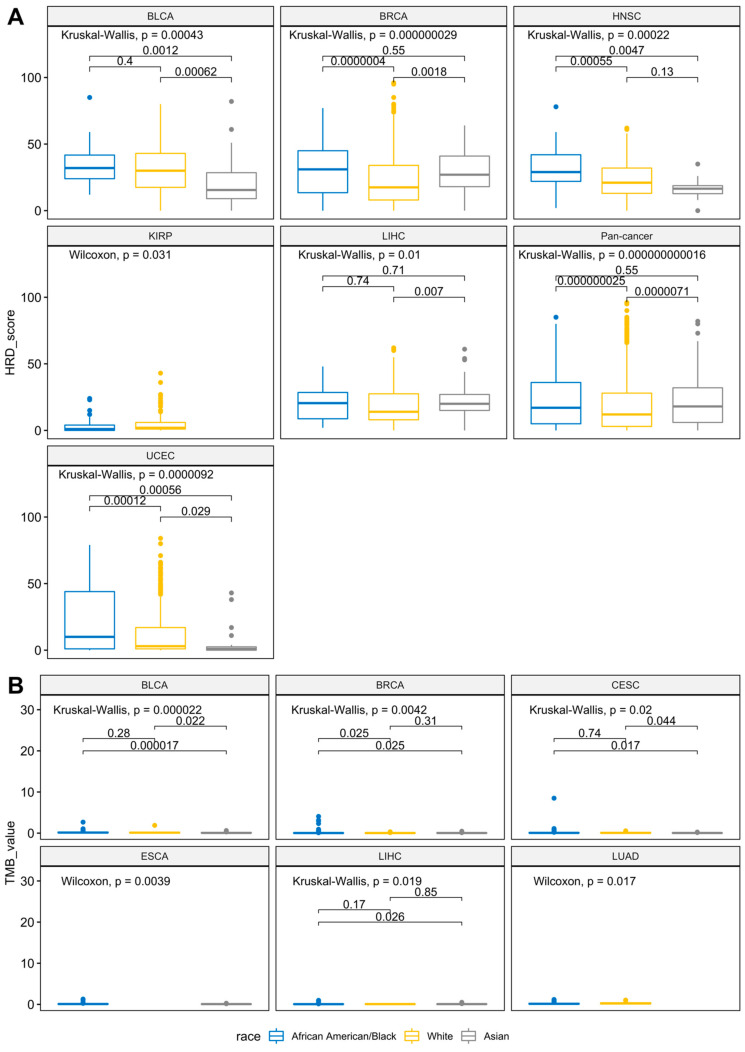
The significant interpopulation differences in the HRD scores and TMB among cancer types and pan-cancer. (**A**) Genome-wide HRD scores of all cancer types are stratified by racial population. (**B**) Global TMB values of all cancer types are stratified by racial population. The Wilcoxon rank-sum test or Kruskal-Wallis test *p* values in each cancer are displayed at the top of each plot. The groupwise Wilcoxon rank-sum test with false discovery rate adjustment *p* values are shown above the bracket for each race-specific comparison.

To explore the racial differences in the genomic instability and mutation patterns across the 24 cancer types, we also examined the genome-wide HRD scores and TMB values, after being stratified by race in each tumor type (Figure 3A,B and Figure S3). Due to the limitations of the sample size following the racial stratification, we only considered cohorts that contained at least two populations with a subgroup sample size larger than 10. Cancers with significant racial differences (*p* < 0.05) in terms of the HRD score or TMB value are summarized in Table S2. Overall, six cancer types, including BLCA, BRCA, and head and neck squamous cell carcinoma (HNSC), had significant differences in terms of the genome-wide HRD scores among the populations (Kruskal-Wallis test; *p* < 0.05). Specifically, based on the pairwise comparison results (Wilcoxon rank-sum test; *p*-adj < 0.05), five cohorts between the ‘White’ and the ‘Asian’ groups (such as BLCA, BRCA, and LIHC), three cohorts between the ‘Asian’ and the ‘African American/Black’ groups (such as BLCA, HNSC, and UCEC), and five cohorts between the ‘White’ and the ‘African American/Black’ groups (such as BRCA, HNSC, and KIRP) were significantly different. Similar to the HRD score, racial differences were also observed in the global TMB values (Table S2). Generally, four cancers, including BLCA, BRCA, and LUAD, were statistically different in terms of the TMB values among all three races. According to the pairwise comparison results (Wilcoxon rank-sum test; *p*-adj < 0.05), five cohorts between the ‘White’ and the ‘Asian’ groups (such as BLCA, BRCA, and LIHC), two cohorts between the ‘Asian’ and the ‘African American/Black’ groups (BLCA and CESC), and two cohorts between the ‘White’ and the ‘African American/Black’ groups (BRCA and LUAD) were significantly different. Collectively, these results demonstrated that racial differences that manifest in molecular changes at the DNA level, such as TMB and HRD, should be considered in many cancers.

### 3.5. Predisposing HRD-Related Genes That Are Specific to Race

**Figure 4:**
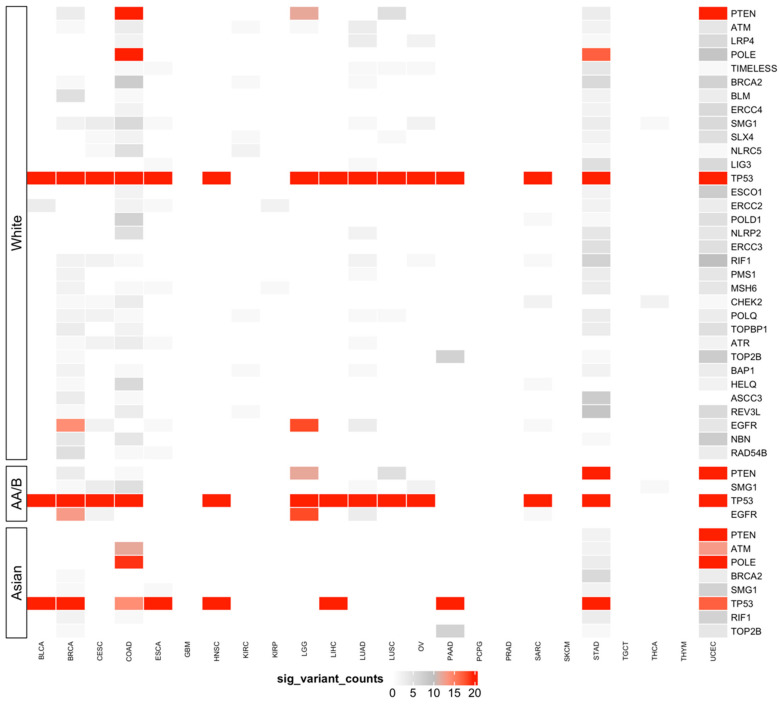
HRD-predisposing genes across 7241 TCGA cases across cancers in the ‘White’, ‘African American/Black’, and ‘Asian’ populations. Race-specific cancer-gene pairs containing HRD-predisposing variants as identified by SKAT-O and an outlier approach. The color scale represents the number of significant variants of predisposing genes within that cancer cohort, and only the genes with more than 10 variants summed in all cancer types are presented. AA/B stands for African American/Black.

We next examined the race-specific pathogenicity of non-synonymous mutations in 364 non-repeated HRD-related genes that were extracted from the five above-mentioned genesets (‘DDRD_assay_41’, ‘mutated_gene_21’, ‘HR_PARP_132’, and ‘DDR_276’) using groupwise association tests (Table 3 and Figures S4–S6). Here, prefiltering mutations with functional meaning, based on two well-known annotation databases (SIFT and PolyPhen-2) that were provided by the Pan-Cancer Atlas, allowed us to focus on those mutations with a biological impact. Four cohorts (GBM, PRAD, SKCM, and TGCT) did not meet the sample size criteria for conducting groupwise association tests, because only one population was available. The top associated predisposing genes and the numbers of their significant variants varied widely across races (Figure 4). The top predisposing HRD genes which were common to all three racial populations were *TP53, RIF1*, and *SMG1*. Some genes were shared only between two populations; for example, *ATM, BRCA2, POLE*, and *TOP2B* were found in both ‘White’ and ‘Asian’ populations, whereas *PTEN* and *EGFG* were observed in both the ‘White’ and ‘African American/Black’ populations. For genes with the highest variant counts in the ‘White’ population, we observed similar mutation levels in other populations. Nevertheless, cancer type-specific differences were apparent. For instance, in BRCA and HNSC, a highly mutated *TP53* was observed in all three populations, whereas in ESCA and PAAD, such an event was only found in the ‘White’ and ‘Asian’ populations. Together, these results highlight the race-based genetic patterns of HRD-related genes that occur in multiple cancers, which can be used to help elucidate customized targeted therapy for each population.

## 4. Discussion

Here we report one of the most extensive multi-race investigations of HRD-predisposing genes, encompassing 7241 samples across 24 cancer types. By using a combination of bioinformatics tools and statistic methods in systematic pan-cancer analysis, we revealed novel insights into race-specific prognostic/therapeutic targets in the HRR pathway with potentially important clinical relevance.

Genome-wide HRD scores have been widely used as a gold standard to evaluate a sample’s HRD status [44,45], and current computational tools were mainly designed to simply count the number of DNA structural changes [36,46]. Such counting results rely heavily on the selected gene lists and the number of genes in that list. Accordingly, our results showed that genome-wide HRD scores were generally higher than the scores that were defined by different gene lists. However, a larger number of genes in the gene list did not guarantee the detection of HRD events that were included in the calculation; for example, the scores that were defined by ‘HR_PARP_132’ were lower than those defined by ‘DDRD_assay_42’ across cancers, reiterating that a good selection of HRD-related genes is important to evaluate the HRD status. In addition, the distribution of HRD scores that were defined by ‘DDR_276’ across cancers showed a good reflection of the genome-wide pattern, but the cut-off value that assigned HRD status might need to be adjusted when using this geneset. Still, these results demonstrated that the ‘DDR_276’ geneset is relatively comprehensive and representative for describing HRD status.

One previous study has demonstrated the synergistic effects between the number of DNA mutations and changes in the copy number [31]. Consistent with this study, our results additionally have revealed that their predictive abilities were relatively similar across many cancers, although HRD scores were associated with a slightly better prediction in terms of overall survival. These findings underscored that, to some extent, different degrees of DNA changes that are related to HRD were correlated in terms of not only their quantity, but also the patient’s prognosis. The racial difference in the HRD scores and TMB values has been systematically investigated in previous studies [18]; however, few of them have considered the Asian population in their research. Hence, in this study, we included as many Asian samples as possible in each cancer cohort to compare these genetic features among racial populations. Our results firstly demonstrated that, in several cancer cohorts, both genetic indicators were significantly different between the ‘White’ and ‘Asian’ populations. This was supported by previous studies using other cancer cohorts [47]. Similar to a previous report, such a difference between the ‘African American/Black’ and ‘Asian’ populations was also observed in HNSC [18]. Intriguingly, genome-wide HRD scores showed that racial differences exist between the ‘White’ population and both other populations at a pan-cancer level. Collectively, these results suggest that considering racial differences, including Asian samples, for a race-based description of HRD status may provide valuable information when defining prognostic groups.

Genomic instability in association with an increased HRD scores has shown a significant impact on the loss of TP53 function across multiple cancers [31,48,49]. Likewise, in our study, cancer-associated TP53 mutations were observed in 15 TCGA cancer types, and such mutations were not associated with the race variable, so this was used as the baseline of our groupwise association tests to support the reliability of the rest of the findings. Our analysis of groupwise association identified mutations in *EGFR* as being specific to the ‘African American/Black’ and ‘White’ populations in BRCA; EGFR is widely used in clinical drug targeting therapy [50]. Mutations in *TOP2B*, which is related to the recruitment of DNA double-strand break repair proteins [51], were exclusive to the ‘Asian’ and ‘White’ populations in PAAD. Additionally, *ATM*, which is involved in DNA damage response pathways [52], was highly mutated in Asian COAD patients. To aid the interpretation of the identified race-specific variants, evidence of pathogenic tendency from public databases provided further support. The majority of the most significant variants that were identified from the association test were likely disease-causing (Figure S7). Additionally, we were able to uncover many race-specific variants that are not currently presented in REVEL or ClinVar. Several associations of significant HRD-predisposing genes and cancer types in specific populations were previously reported for the same population (Table 4 and Table S3), for example, *BAP1* with BRCA in the ‘White’ and ‘African American/Black’ populations [53] and *ATM* with STAD in the ‘White’ and ‘Asian’ populations [54,55]. The association of *BLM* with BRCA in the ‘African American/Black’ and the ‘White’ populations was described for the first time here, but had been identified previously in other populations [56]. While the association of *ERCC5* with OV was first described in the ‘African American/Black’ population here, *BLM* was previously found to be associated with OV in the ‘White’ population [57]. Intriguingly, the association of *MSH6* with STAD was also first identified in the ‘White’ and ’Asian’ populations, while *PTEN* was newly found to be associated with STAD in the ‘African American/Black’ population. However, such associations have been described in other populations [58,59]. These findings, including novel associations, were further supported by older studies that evaluated individual HRD predisposition genes across populations. Overall, the knowledge of different HRD-predisposing genes and their prevalence among populations suggests the importance of incorporating race-specific interpretations into the detection of HRD for achieving the ultimate goal of personalized medicine: a tailored disease diagnosis and intervention based on an individual’s unique HRD pattern.

Leveraging the Pan-Cancer Atlas data, we found multiple significant HRD-predisposing genes for the three populations, yet there was a lack of many cancer cohorts with a sufficient sample size for the ‘Asian’ and ‘African American/Black’ populations. Even when adjusting for confounding factors in statistical tests, such small cancer cohorts likely generated false negatives. To improve the statistical power, we only included cancer–race groups that contained at least 10 samples and applied an outlier approach to stringently filter the most influential variants after the groupwise association test [60]. Sometimes the existence of more variants tended to increase the association in smaller cohorts. It is also necessary to be cautious when interpreting these variants in the context of previous reports. In addition, a racial difference in the sequencing data might affect the reliability of these findings [61]. Furthermore, evidence from population-based databases or public databases that were designed specifically for evaluating the pathogenic tendency of each variant will be needed to provide further validation. The analysis of Pan-Cancer Atlas data has potentially reached saturation in studying the racial differences in genomic features, due to the limited samples of non-White races that constitute a considerable fraction of the US population. Future cancer genomic studies should focus on racial differences in these genetic features in terms of their quantity and prognostic prediction.

## 5. Conclusions

In summary, we identified race-specific predisposing HRD genes and variants contributing to different cancer types. Our analysis of HRD events confirmed the limitations of HRD calculations that are defined by different genesets. Their syngeneic effects and racial differences between them were observed, especially for Asian populations. These results collectively reinforce the importance of considering differences in race when determining the definition, detection, and prognostic value of HRD events. Future studies in larger population-based cohorts are warranted and are prerequisite to conducting HRD-directed precision medicine in patients with distinct genetic backgrounds.

## Figures and Tables

**Table 1:** Summary of demographic distribution of the Pan-Cancer Atlas.

	Asian	African American/Black	White	Total
BLCA	42	22	319	383
BRCA	59	159	678	896
CESC	18	27	184	229
COAD	11	55	184	250
ESCA	44	0	104	148
GBM	0	0	111	111
HNSC	10	46	414	470
KIRC	0	52	287	339
KIRP	0	59	190	249
LGG	0	21	466	487
LIHC	150	16	171	337
LUAD	0	52	376	428
LUSC	0	26	325	351
OV	0	18	146	164
PAAD	11	0	132	143
PCPG	0	19	131	150
PRAD	0	0	143	143
SARC	0	17	202	219
SKCM	0	0	339	339
STAD	75	11	245	331
TGCT	0	0	112	112
THCA	49	26	315	390
THYM	12	0	98	110
UCEC	20	101	341	462
**Total**	**501** **(6.92%)**	**727** **(10.04%)**	**6013** **(83.04%)**	**7241** **(100%)**

**Table 2:** Summary of cancer types **^1^** with significant correlations (*p* < 0.001) between the quantity of DNA mutations (global TMB value) and the changes in the copy number (genome-wide HRD scores).

Positive Correlation	Inverse Correlation
OV (*R* = 0.31)	COAD (*R* = −0.24)
LUSC (*R* = 0.33)	UCEC (*R* = −0.32)
BLCA (*R* = 0.38)	
PAAD (*R* = 0.54)	
LUAD (*R* = 0.49)	
SARC (*R* = 0.5)	
BRCA (*R* = 0.54)	
THYM (*R* = 0.32)	
KIRC (*R* = 0.25)	
HNSC (*R* = 0.23)	
PRAD (*R* = 0.39)	
LGG (*R* = 0.34)	
PCPG (*R* = 0.27)	
Pan-cancer (*R* = 0.26)	

^1^ A full list of TCGA cancer type abbreviations can be found in the Genomic Data Commons (https://gdc.cancer.gov/resources-tcga-users/tcga-code-tables/tcga-study-abbreviations (accessed on 2 June 2021)).

**Table 3:** Summary of the number of significant variants identified by SKAT-O and the RIFT R package.

	White	African American/Black	Asian
BLCA	18	51	11
BRCA	135	97	56
CESC	48	41	2
COAD	200	9	160
ESCA	52	0	52
HNSC	13	20	14
KIRC	27	27	0
KIRP	9	9	0
LGG	17	17	0
LIHC	6	13	4
LUAD	118	118	0
LUSC	52	52	0
OV	18	18	0
PAAD	57	0	57
SARC	25	25	0
STAD	206	20	191
THCA	10	3	7
THYM	2	0	2
UCEC	891	43	213

**Table 4:** Several significant HRD-predisposing gene associations.

Cancer	Gene	Population	Previous Reports		Identified Variant (GRCh38)	REVEL Score ^1^	Clinvar ^2^
			Same Population	Other Population			
BRCA	*BAP1*	White	Shahriyari et al. (2019)		3:52442072-T/C	0.829	pathogenic
		African American/Black					
	*BLM*	White		Cybulski et al. (2019)	15:91312388-C/T	0.677	uncertain significance
		African American/Black					
OV	*ERCC5*	White	Doherty et al. (2011)		3:103525633-G/T	0.939	pathogenic
		African American/Black		Doherty et al. (2011)			
STAD	*ATM*	White	Helgason et al. (2015)		11:108141997-C/T	0.739	uncertain significance
		Asian	Cai et al. (2015)				
	*MSH6*	White		Karpińska-Kaczmarczyk et al. (2016)	2:48028049-G/A	0.857	uncertain significance
		Asian					
	*PTEN*	African American/Black		Nemtsova et al. (2020)	10:89692905-G/A	0.976	likely pathogenic, pathogenic

^1^ REVEL scores above 0.5 represent likely disease-causing variants. ^2^ Five levels of variants defined in ClinVar database are as follows: pathogenic, likely pathogenic, uncertain significance, likely benign, and benign.

## Data Availability

Publicly available datasets were analyzed in this study. This data can be found here: https://gdc.cancer.gov/about-data/publications/pancanatlas (accessed on 2 June 2021).

## Supplementary Materials

The following are available online at https://www.mdpi.com/article/10.3390/jpm11121287/s1, Figure S1: Association of cancer survival with HRD as defined by different genesets. Representative Kaplan-Meier (KM) survival curves for overall survival of four cancer types as a function of HRD. Cancer samples in BRCA, OV, PAAD, PRAD, and pan-cancer were defined as HRD if the HRD score was above the second tertile value (66.7%) within a cancer type. (A) The cohorts with the significant association of survival. (B) The cohorts without significant association of survival. The number of samples in each risk group are displayed at the top of each plot. Log rank test *p* values are presented in the bottom left-hand corner of each plot, Figure S2: Correlation of HRD score and TMB across 24 cancer types and pan-cancer. (A) Spearman correlation between genome-wide HRD score and global TMB value. The TMB value is defined by the number of non-synonymous variants per mega-base in coding regions. The correlation and *p* value in each cancer are displayed in the top of each plot. (B) Association of HRD score, TMB, and their combination with overall survival by fitting Cox proportional hazards models. The c-index values represent the predictivity ability of the model. A value of 0.5 represents a random prediction, a value of 1 equals a perfect prediction, Figure S3: The non-significant interpopulation differences in the HRD scores and TMB among cancer types and pan-cancer. (A) Genome-wide HRD scores of all cancer types are stratified by racial population. (B) Global TMB values of all cancer types are stratified by racial population. Wilcoxon rank-sum test or Kruskal-Wallis test *p* values in each cancer are displayed at the top of each plot. Groupwise Wilcoxon rank-sum test with false discovery rate adjustment *p* values are shown above the bracket for each race-specific comparison, Figure S4: HRD-predisposing genes across cancers in the ‘White’ population. Race-specific cancer–gene pairs containing HRD-predisposing variants were identified by SKAT-O and an outlier approach. The color scale represents the number of significant variants of predisposing genes within each cancer cohort, Figure S5: HRD-predisposing genes across cancers in the ‘African American/Black’ (AA/B) population. Race-specific cancer–gene pairs containing HRD-predisposing variants were identified by SKAT-O and an outlier approach. The color scale represents the number of significant variants of predisposing genes within each cancer cohort, Figure S6: HRD-predisposing genes across cancers in the ‘Asian’ population. Race-specific cancer–gene pairs containing HRD-predisposing variants were identified by SKAT-O and an outlier approach. The color scale represents the number of significant variants of predisposing genes within each cancer cohort, Figure S7: Summary of the validations of the most significant variants identified by groupwise association tests. (A) The histogram of the sample counts of pathogenicity scores of variants validated by the REVEL database are stratified by racial population. Red dashed lines represent the median of the pathogenicity scores across races. REVEL scores above 0.5 represent a likely disease-causing variant. (B) The histogram of the sample counts of pathogenicity scores of variants validated by ClinVar database are stratified by racial population. Although the labels on the graph have various mixtures, five important levels of variants generally defined in this database are as follows: pathogenic, likely pathogenic, uncertain significance, likely benign and benign, Table S1: Summary of cancers with significant racial differences (*p* < 0.05) in terms of the incidence of HRD or global mutation patterns, Table S2: Lists of cancer-associated genes with statistically significant variants that are specific to race, Table S3: Annotation of statistically significant variants identified by RIFT R package according to REVEAL and ClinVar databases.
