# Supplementary material for: Race-Specific Genetic Profiles of Homologous Recombination Deficiency in Multiple Cancers
Source: J Pers Med. 2021 Dec 3;11(12):1287. doi: 10.3390/jpm11121287 (PMC8705317; doi:10.3390/jpm11121287)
Supplement: Supplementary file 1 [file jpm-11-01287-s001.zip › jpm-1472742-supplementary.pdf]

Supplementary Materials

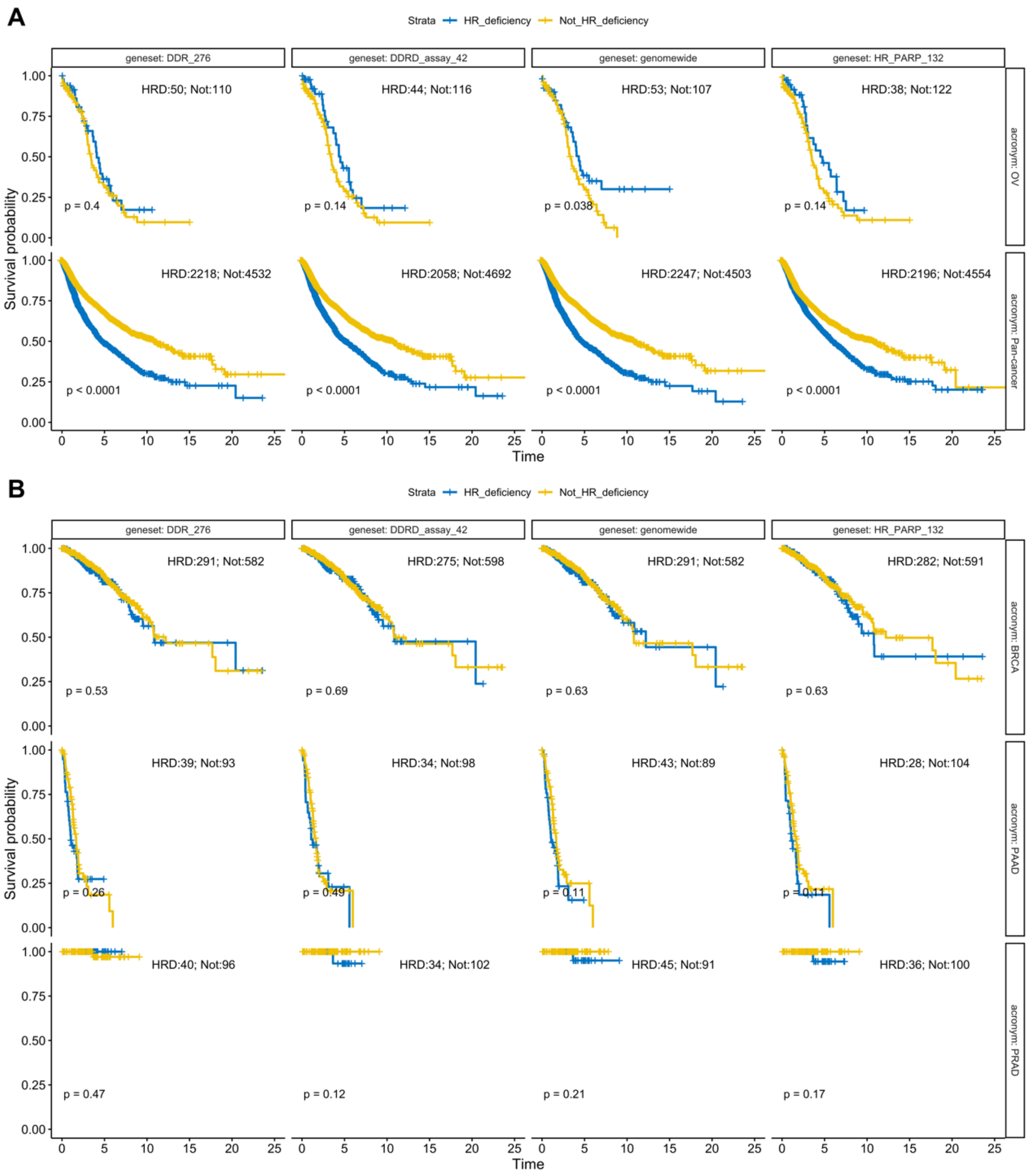

Figure S1. Association of cancer survival with HRD as defined by different genesets. Representative Kaplan-Meier (KM) survival curves for overall survival of four cancer types as a function of HRD. Cancer samples in BRCA, OV, PAAD, PRAD, and pan-cancer were defined as HRD if the HRD score was above the second tertile value (66.7%) within a cancer type. (A) The cohorts with the significant association of survival. (B) The cohorts without significant association of survival. The number of samples in each risk group are displayed at the top of each plot. Log rank test *p* values are presented in the bottom left-hand corner of each plot.

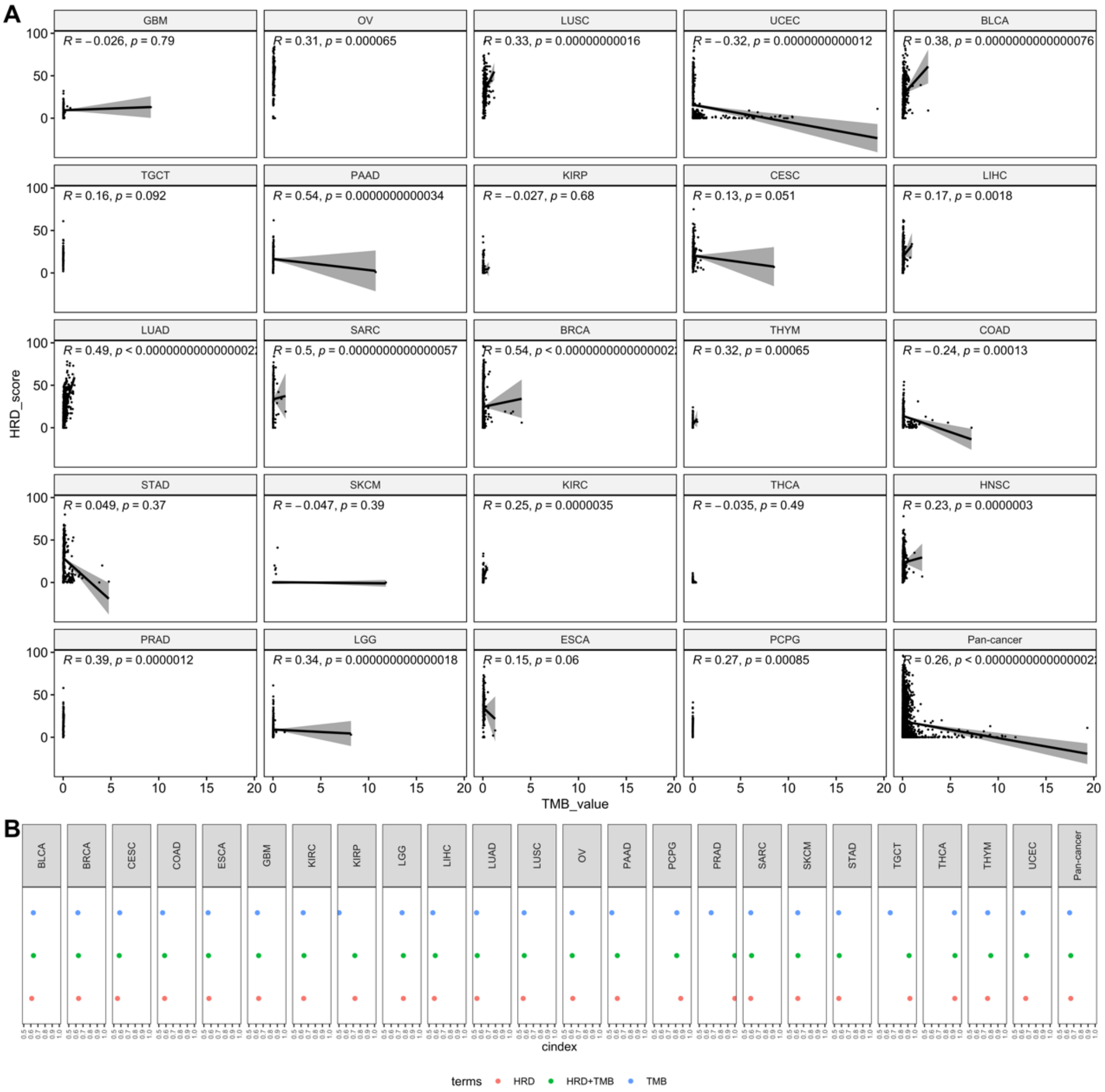

Figure S2. Correlation of HRD score and TMB across 24 cancer types and pan-cancer. (A) Spearman correlation between genome-wide HRD score and global TMB value. The TMB value is defined by the number of non-synonymous variants per mega-base in coding regions. The correlation and *p* value in each cancer are displayed in the top of each plot. (B) Association of HRD score, TMB, and their combination with overall survival by fitting Cox proportional hazards models. The c-index values represent the predictivity ability of the model. A value of 0.5 represents a random prediction, a value of 1 equals a perfect prediction.

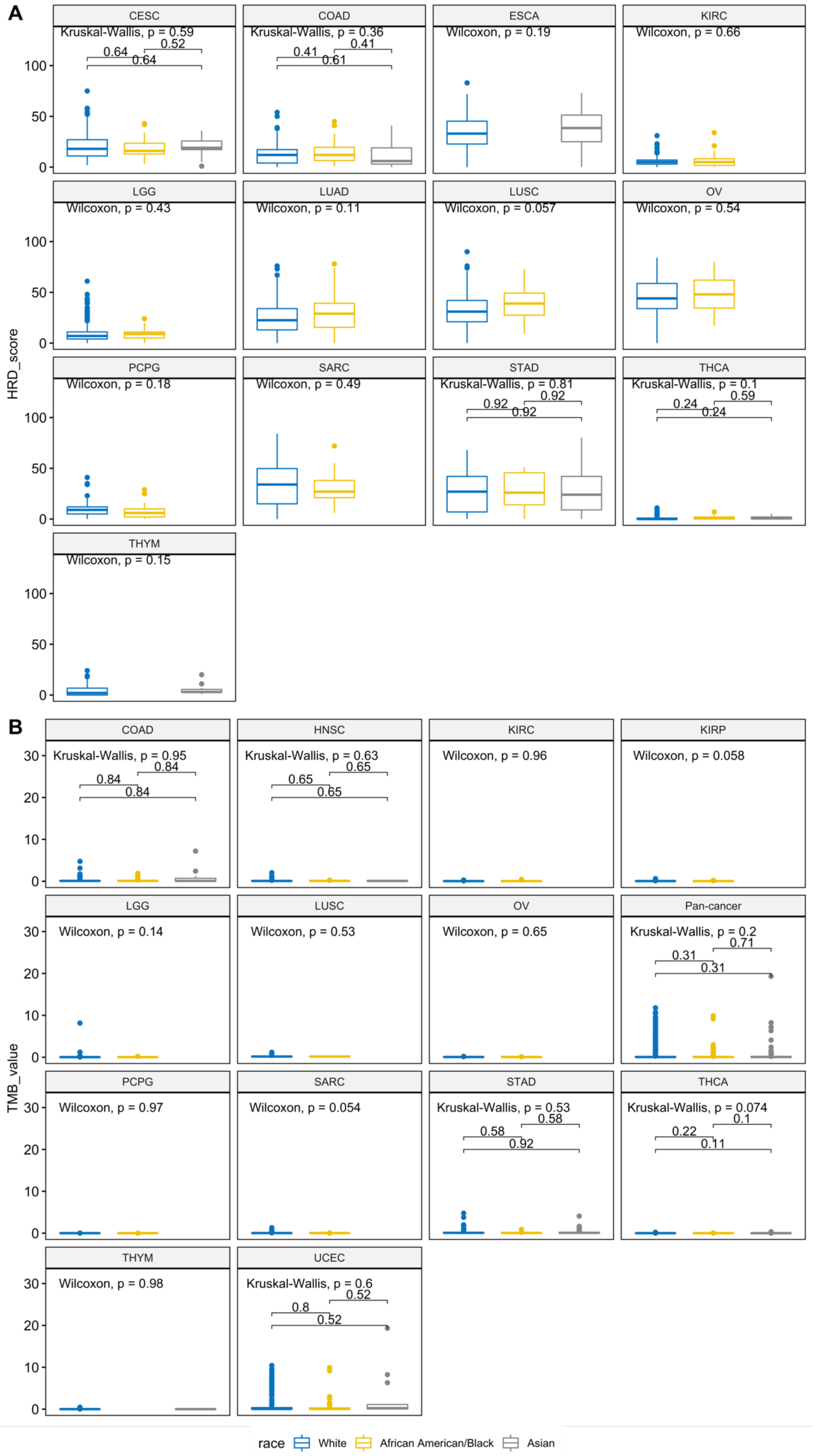
A
CESC
Kruskal-Wallis, p = 0.59
0.64
0.52
0.64
COAD
Kruskal-Wallis, p = 0.36
0.41
0.41
0.61
ESCA
Wilcoxon, p = 0.19
KIRC
Wilcoxon, p = 0.66
LGG
Wilcoxon, p = 0.43
LUAD
Wilcoxon, p = 0.11
LUSC
Wilcoxon, p = 0.057
OV
Wilcoxon, p = 0.54
PCPG
Wilcoxon, p = 0.18
SARC
Wilcoxon, p = 0.49
STAD
Kruskal-Wallis, p = 0.81
0.92
0.92
0.92
THCA
Kruskal-Wallis, p = 0.1
0.24
0.59
0.24
THYM
Wilcoxon, p = 0.15
HRD_score
0
50
100
B
COAD
Kruskal-Wallis, p = 0.95
0.84
0.84
0.84
HNSC
Kruskal-Wallis, p = 0.63
0.65
0.65
0.65
KIRC
Wilcoxon, p = 0.96
KIRP
Wilcoxon, p = 0.058
LGG
Wilcoxon, p = 0.14
LUSC
Wilcoxon, p = 0.53
OV
Wilcoxon, p = 0.65
Pan-cancer
Kruskal-Wallis, p = 0.2
0.31
0.71
0.31
PCPG
Wilcoxon, p = 0.97
SARC
Wilcoxon, p = 0.054
STAD
Kruskal-Wallis, p = 0.53
0.58
0.58
0.92
THCA
Kruskal-Wallis, p = 0.074
0.22
0.1
0.11
THYM
Wilcoxon, p = 0.98
UCEC
Kruskal-Wallis, p = 0.6
0.8
0.52
0.52
TMB_value
0
10
20
30
race
White
African American/Black
Asian

Figure S3. The non-significant interpopulation differences in the HRD scores and TMB among cancer types and pan-cancer. (A) Genome-wide HRD scores of all cancer types are stratified by racial population. (B) Global TMB values of all cancer types are stratified by racial population. Wilcoxon rank-sum test or Kruskal-Wallis test *p* values in each cancer are displayed at the top of each plot. Groupwise Wilcoxon rank-sum test with false discovery rate adjustment *p* values are shown above the bracket for each race-specific comparison.

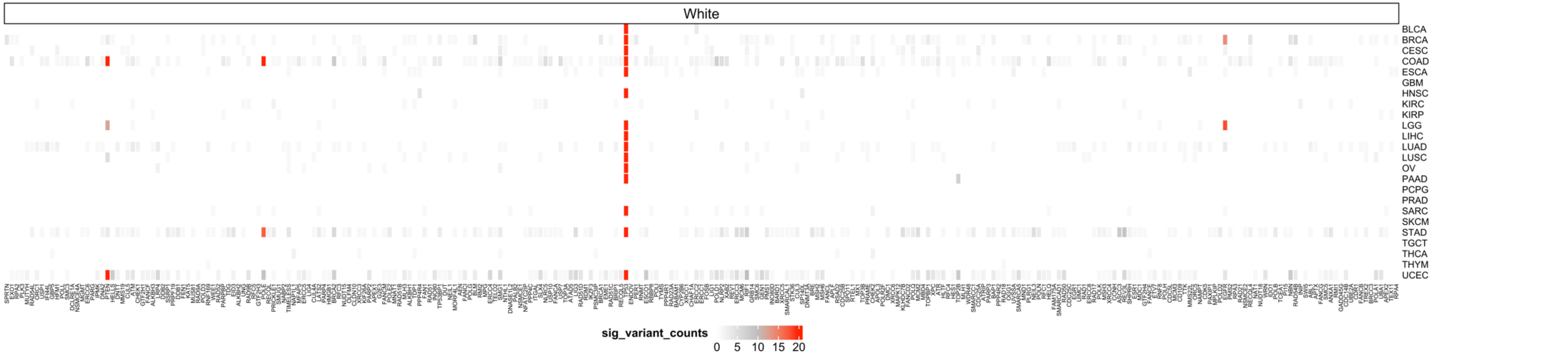

Figure S4. HRD-predisposing genes across cancers in the ‘White’ population. Race-specific cancer–gene pairs containing HRD-predisposing variants were identified by SKAT-O and an outlier approach. The color scale represents the number of significant variants of predisposing genes within each cancer cohort.

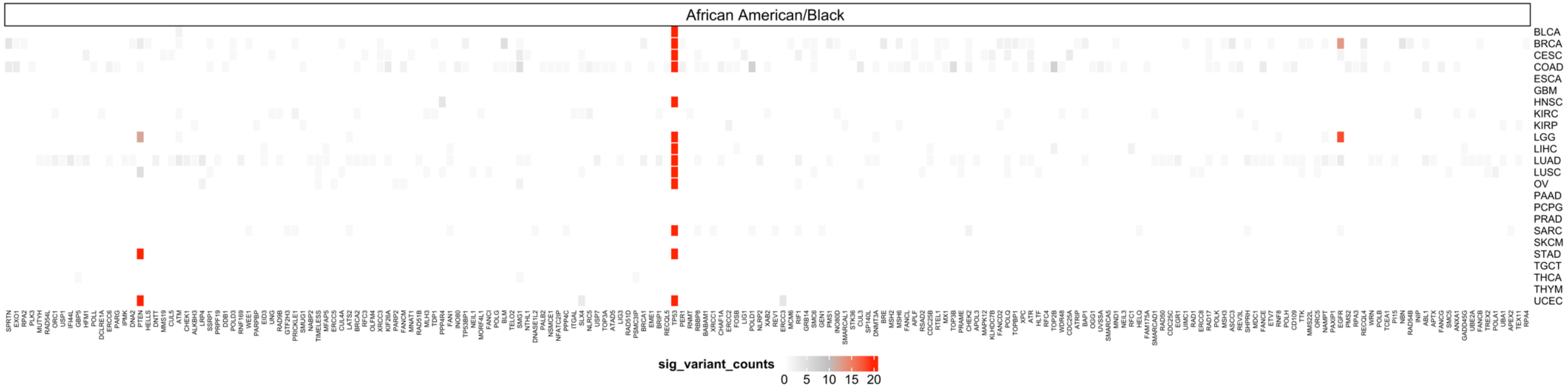

Figure S5. HRD-predisposing genes across cancers in the 'African American/AA/B' (AA/B) population. Race-specific cancer–gene pairs containing HRD-predisposing variants were identified by SKAT-O and an outlier approach. The color scale represents the number of significant variants of predisposing genes within each cancer cohort.

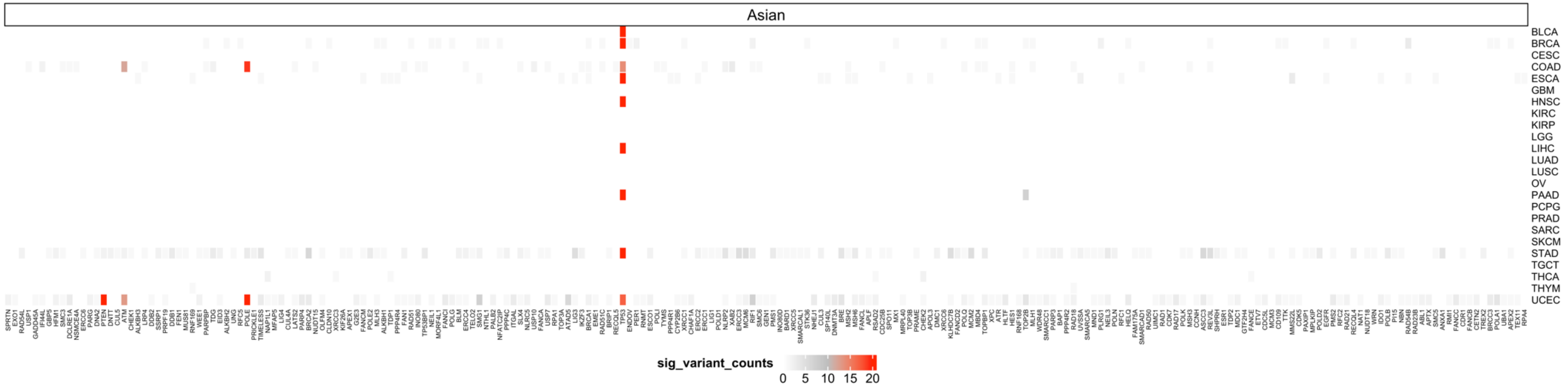

Figure S6. HRD-predisposing genes across cancers in the 'Asian' population. Race-specific cancer–gene pairs containing HRD-predisposing variants were identified by SKAT-O and an outlier approach. The color scale represents the number of significant variants of predisposing genes within each cancer cohort.

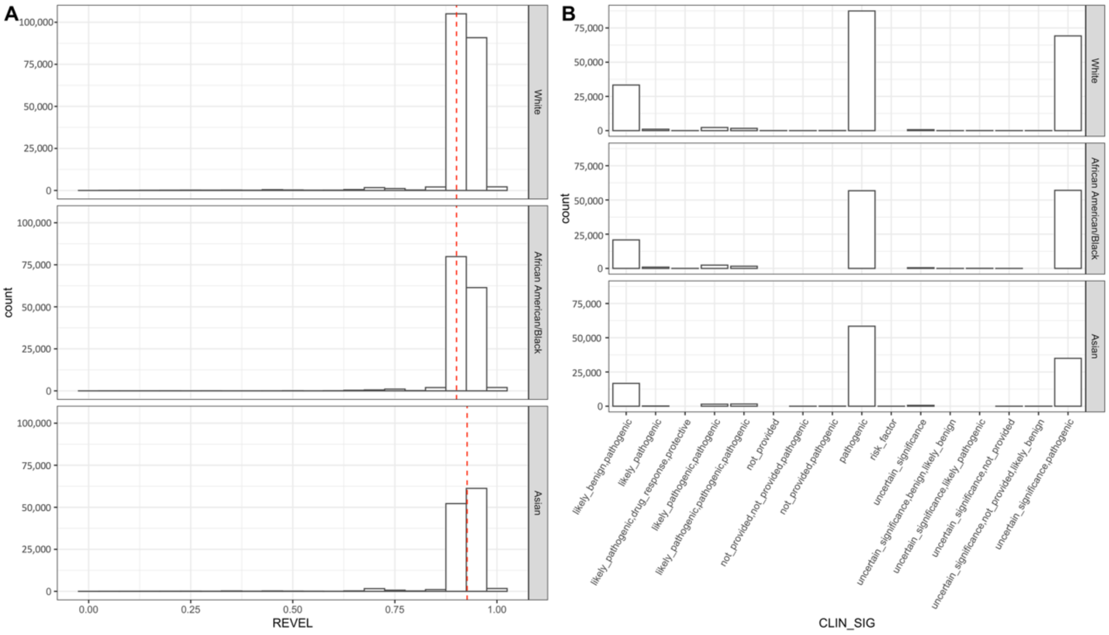

Figure S7. Summary of the validations of the most significant variants identified by groupwise association tests. (A) The histogram of the sample counts of pathogenicity scores of variants validated by the REVEL database are stratified by racial population. Red dashed lines represent the median of the pathogenicity scores across races. REVEL scores above 0.5 represent a likely disease-causing variant. (B) The histogram of the sample counts of pathogenicity scores of variants validated by ClinVar database are stratified by racial population. Although the labels on the graph have various mixtures, five important levels of variants generally defined in this database are as follows: pathogenic, likely pathogenic, uncertain significance, likely benign and benign.

Table S1. Summary of cancers with significant racial differences ($p < 0.05$) in terms of the incidence of HRD or global mutation patterns.

Among three populations[1]	Between two populations[1]		
	White vs. AA/B	White vs. Asian	AA/B vs. Asian
● *Genetic features: HRD*			
**BLCA (p<0.001)**[2]	**BRCA (p<0.001)**	**BLCA (p<0.001)**	**BLCA (p=0.0012)**
**BRCA (p<0.001)**	HNSC (p<0.001)	**BRCA (p=0.0018)**	HNSC (p=0.0047)
HNSC (p<0.001)	KIRP (p=0.031)	**LIHC (p=0.007)**	UCEC (p<0.001)
**LIHC (p=0.01)**	UCEC (p<0.001)	UCEC (p=0.029)	
UCEC (p<0.001)	Pan-cancer (p<0.001)	Pan-cancer (p<0.001)	
Pan-cancer (p<0.001)			
● *Genetic features: TMB*			
**BLCA (p<0.001)**	**BRCA (p=0.025)**	**BLCA (p<0.001)**	**BLCA (p=0.022)**
**BRCA (p<0.001)**	LUAD (p=0.017)	**BRCA (p=0.025)**	CESC (p=0.044)
CESC (p = 0.02)		CESC (p=0.017)	
**LIHC (p = 0.019)**		ESCA (p<0.001)	
		**LIHC (p=0.026)**	

[1] Wilcoxon rank-sum tests or Kruskal-Wallis tests were conducted to compare the difference of these genetic features across populations and the false discovery rate was used for the correlation of multiple comparisons if necessary. A p-value of less than 0.05 is reported as statistically significant.

[2] A bold font indicates that both genetic features showed racial differences in these cancer types.

Table S2. Lists of cancer-associated genes with statis-tically significant variants that are specific to race.

Race	Cancer	# of sig_variants	# of sig_genes	Hugo_Symbol
Asian	BLCA	7	1	*TP53*
Asian	BRCA	62	41	*ATM,TP53*
Asian	COAD	47	43	*TP53,ERCC2*
Asian	ESCA	57	33	*PTPRC,PARP1,PARPBP,ALKBH2,RFC5,BRCA2,CLDN10,MLH3,ALKBH1,RAD51,NEIL1,MORF4L1,POLG,SMG1,NTHL1,NFATC2IP,BRCA1,RAD51C,TP53,ENDOV,PER1,XRCC1,RIF1,STK36,MX1,XRCC6,MBD4,TOPBP1,TOP2B,MLH1,PLRG1,HELQ,REV3L,CD109,TTK,EGFR,RECQL4,RAD54B,BRCC3,POLA1,APEX2*
Asian	HNSC	12	1	*SPRTN,EXO1,RPA2,DNA2,PTEN,HELLS,ATM,SSRP1,POLD3,WEE1,PRICKLE1,CUL4A,XRCC3,TDP1,TP53BP1,BLM,SMG1,BRCA1,TP53,PER1,FOSB,MCM6,GRB14,SMC6,PMS1,INO80D,BRE,MSH6,APLF,RTEL1,MX1,CHEK2,FANCD2,POLQ,TOPBP1,XPC,ATR,ATRIP,BAP1,MND1,UIMC1,RAD17,MSH3,ASCC3,SHPRH,ETV7,TTK,MMS22L,NAMPT,EGFR,RECQL4,NBN,RAD54B,ABL1,FANCB,RPA4*
Asian	LIHC	4	1	*PTPRC,PARP1,SPRTN,EXO1,RPA2,DNA2,PTEN,HELLS,ATM,SSRP1,POLD3,WEE1,PARPBP,ALKBH2,RFC5,PRICKLE1,CUL4A,BRCA2,CLDN10,XRCC3,MLH3,ALKBH1,TDP1,RAD51,TP53BP1,NEIL1,MORF4L1,POLG,BLM,SMG1,NTHL1,NFATC2IP,BRCA1,RAD51C,TP53,ENDOV,PER1,XRCC1,FOSB,MCM6,RIF1,GRB14,SMC6,PMS1,INO80D,STK36,MSH6,APLF,RTEL1,MX1,CHEK2,XRCC6,FANCD2,POLQ,MBD4,TOPBP1,XPC,ATR,TOP2B,MLH1,ATRIP,BAP1,MND1,PLRG1,HELQ,UIMC1,RAD17,MSH3,ASCC3,REV3L,SHPRH,ETV7,CD109,TTK,MMS22L,NAMPT,EGFR,RECQL4,NBN,RAD54B,ABL1,FANCB,BRCC3,POLA1,APEX2,RPA4*

Asian	PAAD	6	2	*CLK2,HFM1,MMS19,CUL5,POLD3,UNG,RFC3,XRCC3,KIF26A,MNAT1,SMG1,NTHL1,SLX4,TP53,RBBP8,LIG1,RIF1,SMC6,STK36,APLF,RTEL1,CHEK2,MAPK12,KLHDC7B,POLQ,ATR,CDC25A,RAD17,EGFR,RECQL4*
Asian	STAD	205	126	*CLK2,UBE2T,RAD54L,HFM1,MMS19,CUL5,POLD3,UNG,RFC3,XRCC3,KIF26A,MNAT1,SMG1,NTHL1,SLX4,NLRC5,TP53,RBBP8,FOSB,LIG1,RIF1,SMC6,STK36,APLF,RTEL1,CHEK2,MAPK12,KLHDC7B,POLQ,ATR,CDC25A,RAD17,GTF2H4,EGFR,RECQL4*
Asian	THCA	7	6	*PTPRC,USP1,IFI44L,SMC3,DCLRE1A,NSMCE4A,ATM,LRP4,PARPBP,TDG,POLE,LATS2,NUDT15,FAN1,INO80,TP53BP1,ERCC4,SLX4,USP10,USP7,IKZF3,BRCA1,RAD51C,RECQL5,TP53,POLI,TYMS,ERCC1,NLRP2,XAB2,RIF1,SMC6,MSH2,CDC25B,POLQ,MBD4,TOPBP1,HES1,MLH1,RAD18,SMARCAD1,MSH3,REV3L*
Asian	THYM	2	2	*ADAMTS4,PTPRC,SPRTN,EXO1,PLK3,POLL,ERCC6,PARG,PTEN,SSRP1,DDB1,SMUG1,BRCA2,XRCC3,KIF26A,FANCM,PPP4R4,INO80,TP53BP1,POLG,BLM,TELO2,SMG1,PALB2,NSMCE1,NFATC2IP,PPP4C,SLX4,NLRC5,USP7,TOP3A,ATAD5,RAD51D,EME1,RECQL5,TP53,PER1,RBBP8,BABAM1,CHAF1A,ERCC2,FOSB,POLD1,XAB2,SMC6,CUL3,DNMT3A,MSH2,MSH6,FANCL,RSAD2,CDC25B,MX1,TOP3B,CHEK2,KLHDC7B,TOPBP1,ATR,RFC4,TOP2B,WDR48,OGG1,UVSSA,SMARCA5,NEIL3,FAM175A,RAD17,POLK,ASCC3,REV3L,FANCE,POLH,CD109,PMS2,RPA3,RECQL4,TCEA1,ABL1,FANCG,ANXA1*
Asian	UCEC	231	133	*ADAMTS4,PTPRC,PARP1,EXO1,PLK3,USP1,IFI44L,HFM1,POLL,SMC3,DCLRE1A,NSMCE4A,ERCC6,PARG,DNA2,PTEN,CUL5,ATM,CHEK1,LRP4,SSRP1,DDB1,PARPBP,TDG,RAD9B,POLE,PRICKLE1,SMUG1,TIMELESS,NAP1L1,MFAP5,ERCC5,CUL4A,LATS2,BRCA2,NUDT15,XRCC3,KIF26A,G2E3,FANCM,MLH3,PPP4R4,FAN1,INO80,TP53BP1,FANCI,POLG,BLM,MPG,ERCC4,TELO2,SMG1,PALB2,NSMCE1,NFATC2IP,PPP4C,SLX4,NLRC5,USP10,USP7,TOP3A,ATAD5,RAD51D,IKZF3,BRCA1,EME1,RAD51C,BRIP1,RECQL5,TP53,PER1,ESCO1,RB*

				BP8,POLI,TYMS,PPP4R1,BABAM1,CHAF1A,ERCC2,ERCC1,FOSB,LIG1,POLD1,NLRP2,XAB2,RIF1,SMC6,XRCC5,CUL3,DNMT3A,MSH2,MSH6,FANCL,RSAD2,CDC25B,SPO11,RTEL1,MX1,TOP3B,CHEK2,POLR2F,KLHDC7B,POLQ,MBD4,TOPBP1,XPC,ATR,RFC4,HES1,MLH1,WDR48,CDC25A,BAP1,RAD18,OGG1,UVSSA,SMARCA5,NEIL3,HELQ,FAM175A,SMARCAD1,RAD50,CDC25C,RAD17,POLK,MSH3,ASCC3,REV3L,RNF8,POLH,CD109,NAMPT,CDK5,PMS2,RPA3,RAD21,NSMCE2,RECQL4,TCEA1,NBN,RAD54B,ABL1,APTX,FANCG,SMC5,ANXA1,RMI1,GADD45G,POLA1
AA/B	BLCA	5	2	*UBE2T,ALKBH3,RNF169,TIMELESS,FANCM,ALKBH1,TDP1,PPP4R4,SMG1,TOP3A,LIG3,BRCA1,TP53,PPP4R1,CYP2B6,ERCC2,CUL3,SP140L,MSH2,MSH6,TOP3B,APOL3,ATR,HES1,UVSSA,RFC1,MMS22L,EGFR,IDO1,RAD54B,SMC5,TEX11,RPA4*
AA/B	BRCA	103	56	*UBE2T,ALKBH3,RNF169,TIMELESS,FANCM,ALKBH1,TDP1,PPP4R4,SMG1,TOP3A,LIG3,BRCA1,TP53,PPP4R1,CYP2B6,ERCC2,CUL3,SP140L,MSH2,MSH6,TOP3B,APOL3,ATR,HES1,UVSSA,RFC1,MMS22L,EGFR,IDO1,RAD54B,SMC5,TEX11,RPA4*
AA/B	CESC	46	30	*TP53*
AA/B	COAD	112	80	*PPP4R4,TP53*
AA/B	HNSC	17	2	*PPP4R4,TP53,SP140L*
AA/B	KIRC	31	25	*ORC1,DCLRE1A,ATM,UNG,RAD9B,PRICKLE1,BRCA2,MLH3,TDP1,MORF4L1,ITGAL,SLX4,NLRC5,BRIP1,RNMT,XAB2,INO80D,POLQ,WDR48,BAP1,SMARCAD1,REV3L,INIP,ANXA1,UBE2A*
AA/B	KIRP	10	10	*ORC1,DCLRE1A,ATM,UNG,RAD9B,PRICKLE1,BRCA2,MLH3,TDP1,MORF4L1,ITGAL,SLX4,NLRC5,BRIP1,RNMT,XAB2,INO80D,POLQ,WDR48,BAP1,SMARCAD1,REV3L,INIP,ANXA1,UBE2A*
AA/B	LGG	18	8	*ALKBH3,PARPBP,SMUG1,ERCC2,SMARCAL1,MSH6,FANCD2,MDC1,UBA1,TEX11*

AA/B	LIHC	13	9	*ALKBH3,PARPBP,SMUG1,ERCC2,SMARCAL1,MSH6,FANCD2,MDC1,UBA1,TEX11*
AA/B	LUAD	126	83	*PTEN,ATM,LATS2,TP53,DNMT3A,RNF8,NAMPT,EGFR*
AA/B	LUSC	46	23	*PTEN,ATM,LATS2,TP53,DNMT3A,RNF8,NAMPT,EGFR*
AA/B	OV	21	10	*TP53*
AA/B	SARC	25	20	*EID3,MFAP5,FAN1,TP53,FOSB,CDC25B,TOP2B,RFC1,GADD45G*
AA/B	STAD	12	2	*TP53*
AA/B	THCA	3	3	*DCLRE1B,MAD2L2,PTPRC,MUTYH,RAD54L,ORC1,USP1,IFI44L,GBP5,HFM1,ERCC6,IPMK,DNA2,DNTT,CUL5,ATM,CHEK1,ALKBH3,LRP4,PRPF19,RNF169,EID3,RAD9B,NABP2,TIMELESS,MFAP5,LATS2,BRCA2,RFC3,OLFM4,KIF26A,RAD51B,FAN1,SMG1,NTHL1,USP7,LIG3,BRCA1,BRIP1,TP53,RBBP8,CHAF1A,NLRP2,RIF1,PMS1,SP140L,DNMT3A,CDC25B,TOP3B,PRAME,APOL3,POLQ,XPC,ATR,BAP1,SMARCAD1,RAD50,CDC25C,EGR1,RAD17,MSH3,SHPRH,MDC1,FANCE,ETV7,POLH,CD109,TTK,ORC5,NAMPT,PAXIP1,EGFR,WRN,POLB,PI15,ABL1,APTX,GADD45G,UBE2A,FANCB,TREX2,UBA1,TEX11*
AA/B	UCEC	8	4	*DCLRE1B,MAD2L2,PTPRC,MUTYH,RAD54L,ORC1,USP1,IFI44L,GBP5,HFM1,ERCC6,IPMK,DNA2,DNTT,CUL5,ATM,CHEK1,ALKBH3,LRP4,PRPF19,RNF169,EID3,RAD9B,NABP2,TIMELESS,MFAP5,LATS2,BRCA2,RFC3,OLFM4,KIF26A,RAD51B,FAN1,SMG1,NTHL1,USP7,LIG3,BRCA1,BRIP1,TP53,RBBP8,CHAF1A,NLRP2,RIF1,PMS1,SP140L,DNMT3A,CDC25B,TOP3B,PRAME,APOL3,POLQ,XPC,ATR,BAP1,SMARCAD1,RAD50,CDC25C,EGR1,RAD17,MSH3,SHPRH,MDC1,FANCE,ETV7,POLH,CD109,TTK,ORC5,NAMPT,PAXIP1,EGFR,WRN,POLB,PI15,ABL1,APTX,GADD45G,UBE2A,FANCB,TREX2,UBA1,TEX11*
White	BLCA	8	2	*ADAMTS4,PTEN,SSRP1,TIMELESS,CUL4A,MLH3,PPP4R4,NEIL1,FANCI,SLX4,TP53,SMC6,RSAD2,PRAME,POLQ,HLTF,RAD1,ERCC8,TTK,ORC5,SMC5,TREX2,POLA1*

White	BRCA	149	86	*ADAMTS4,PTEN,SSRP1,TIMELESS,CUL4A,MLH3,PPP4R4,NEIL1,FANCI,SLX4,TP53,SMC6, RSAD2,PRAME,POLQ,HLTF,RAD1,ERCC8,TTK,ORC5,SMC5,TREX2,POLA1*
White	CESC	54	35	*ADAMTS4,LRP4,TIMELESS,ERCC5,PARP2,FANCM,SMG1,TP53,RIF1,CUL3*
White	COAD	262	149	*ADAMTS4,LRP4,TIMELESS,ERCC5,PARP2,FANCM,SMG1,TP53,RIF1,CUL3*
White	ESCA	57	33	*TP53,TOP2B*
White	HNSC	19	3	*TP53,TOP2B*
White	KIRC	31	25	*DCLRE1B,WEE1,GTF2H3,PRICKLE1,LATS2,FAN1,DNASE1L2,PPP4C,TP53,RBBP8,XRCC1, POLD1,REV1,RIF1,GEN1,CHEK2,HELQ,SHPRH,EGFR,APEX2*
White	KIRP	10	10	*DCLRE1B,WEE1,GTF2H3,PRICKLE1,LATS2,FAN1,DNASE1L2,PPP4C,TP53,RBBP8,XRCC1, POLD1,REV1,RIF1,GEN1,CHEK2,HELQ,SHPRH,EGFR,APEX2*
White	LGG	18	8	*DCLRE1B,CD2,ADAMTS4,PTPRC,RAD54L,GBP5,HFM1,SMC3,ERCC6,DNA2,PTEN,DNTT,CUL5,ATM,CHEK1,LRP4,SSRP1,PRPF19,DDB1,FEN1,MUS81,WEE1,PARPBP,TDG,EID3,UNG,POLE,PRICKLE1,TIMELESS,CUL4A,LATS2,BRCA2,OLFM4,KIF26A,APEX1,FANCM,POLE2,MLH3,ALKBH1,FAN1,TP53BP1,BLM,ERCC4,TELO2,SMG1,NFATC2IP,PPP4C,SLX4,NLRC5,FANCA,USP7,LIG3,IKZF3,BRIP1,TP53,ESCO1,XRCC1,ERCC2,ERCC1,LIG1,POLD1,NLRP2,XAB2,ERCC3,MCM6,RIF1,PMS1,INO80D,BARD1,XRCC5,SMARCAL1,STK36,CUL3,BRE, MSH2,MSH6,APLF,CDC25B,SPO11,PRAME,DMC1,KLHDC7B,FANCD2,POLQ,MCM2,TOPBP1,TOP2B,WDR48,SMARCC1,PARP3,BAP1,RAD18,UVSSA,SMARCA5,MND1,PLRG1,NEIL3, POLN,FAM175A,SMARCAD1,RAD50,POLK,MSH3,ASCC3,REV3L,SHPRH,ESR1,MDC1,GTF2H4,MCM3,MMS22L,PAXIP1,MPLKIP,POLD2,PMS2,RECQL4,NUDT18,WRN,POLB,PI15,NBN,SMC5,ANXA1,CDR1,TREX2,APEX2*
White	LIHC	4	1	*PTEN,TP53*

White	LUAD	126	83	*DCLRE1B,CD2,ADAMTS4,PTPRC,RAD54L,USP1,GBP5,HFM1,SMC3,MGMT,ERCC6,DNA2,PTEN,DNTT,MMS19,CUL5,ATM,CHEK1,LRP4,SSRP1,PRPF19,DDB1,FEN1,MUS81,RNF169,WEE1,PARPBP,TDG,EID3,UNG,POLE,RECQL,PRICKLE1,TIMELESS,CUL4A,LATS2,PARP4,BRCA2,NUDT15,OLFM4,XRCC3,KIF26A,APEX1,FANCM,POLE2,MLH3,ALKBH1,FAN1,TP53BP1,MORF4L1,FANCI,BLM,RMI2,ERCC4,TELO2,SMG1,NFATC2IP,PPP4C,ITGAL,SLX4,NLRC5,USP10,FANCA,USP7,LIG3,RDM1,IKZF3,BRIP1,TP53,RNMT,ESCO1,CYP2B6,XRCC1,ERCC2,ERCC1,LIG1,POLD1,NLRP2,XAB2,ERCC3,MCM6,RIF1,PMS1,INO80D,BARD1,XRCC5,SMARCAL1,STK36,CUL3,BRE,MSH2,MSH6,APLF,CDC25B,SPO11,PRAME,DMC1,KLHDC7B,FANCD2,POLQ,MCM2,TOPBP1,HLTF,TOP2B,WDR48,SMARCC1,PARP3,BAP1,RAD18,UVSSA,SMARCA5,MND1,PLRG1,NEIL3,POLN,FAM175A,SMARCAD1,RAD50,RAD17,POLK,MSH3,XRCC4,ASCC3,REV3L,SHPRH,ESR1,MDC1,GTF2H4,CDC5L,MCM3,MMS22L,PAXIP1,MPLKIP,POLD2,PMS2,NSMCE2,RECQL4,NUDT18,WRN,POLB,PI15,NBN,ABL1,SMC5,ANXA1,CDC14B,CDR1,TREX2,UBA1,APEX2*
White	LUSC	46	23	*NAP1L1,XRCC3,TDP1,RSAD2,CHEK2,FANCE*
White	OV	21	10	*GBP5,SMG1,PSMC3IP*
White	PAAD	6	2	*GBP5,NAP1L1,XRCC3,TDP1,SMG1,PSMC3IP,RSAD2,CHEK2,FANCE*
White	SARC	25	20	*RNF169,RAD18*
White	STAD	269	150	*RNF169,RAD18*
White	THCA	10	9	*PRMT6,DCLRE1B,BCAS2,PTPRC,SPRTN,EXO1,GADD45A,IFI44L,HFM1,SMC3,DCLRE1A,PARG,DNA2,PTEN,ATM,DDB2,PRPF19,WEE1,EID3,POLE,TIMELESS,NAP1L1,MFAP5,LIG4,LATS2,PARP4,BRCA2,OLFM4,G2E3,FANCM,MLH3,PPP4R4,FAN1,RAD51,INO80,NEIL1,FANCI,POLG,BLM,ERCC4,SMG1,PALB2,PPP4C,ITGAL,SLX4,NLRC5,USP7,RPA1,ATAD5,LIG3,BRCA1,EME1,BRIP1,TP53,PER1,RNMT,ESCO1,CYP2B6,XRCC1,CHAF1A,NLRP2,ERCC3,M*

				CM6,RIF1,SMC6,GEN1,PMS1,NHEJ1,SP140L,DNMT3A,BRE,MSH6,FANCL,APLF,RSAD2,MRPL40,CHEK2,KLHDC7B,POLQ,MCM2,TOPBP1,XPC,ATR,HLTF,RNF168,TOP2B,MLH1,WDR48,SMARCC1,PARP3,PPP4R2,RAD18,MND1,PLRG1,NEIL3,HELQ,UIMC1,RAD1,CDK7,RAD17,POLK,CCNH,ASCC3,REV3L,TDP2,MDC1,ETV7,CDC5L,CD109,TTK,MMS22L,CDK5,PMS2,RFC2,RAD21,NAT1,IDO1,POLB,NBN,RAD54B,RAD23B,ABL1,APTX,RMI1,FANCC,CDR1,FANCB,CETN2,BRCC3,POLA1,UBA1,APEX2,TEX11
White	THYM	2	2	*PTEN,SLX4,TP53,ERCC3*
White	UCEC	312	158	*BCAS2,CD2,CLK2,ADAMTS4,PTPRC,PARP1,EXO1,RPA2,PLK3,MUTYH,ORC1,GBP5,SMC3,IPMK,PTEN,HELLS,DNTT,CUL5,ATM,GTF2H1,FANCF,ALKBH3,LRP4,DDB2,SSRP1,DDB1,KAT5,RAD9A,RAD52,EID3,RAD9B,POLE,PRICKLE1,TIMELESS,MFAP5,ERCC5,LIG4,PARP4,HMGB1,BRCA2,PARP2,FANCM,POLE2,MNAT1,MLH3,ALKBH1,PPP4R4,FAN1,INO80,TP53BP1,DUT,NEIL1,AEN,FANCI,BLM,ERCC4,SMG1,PPP4C,ITGAL,SLX4,NLRC5,FANCA,USP7,TOP3A,ATAD5,LIG3,RAD51D,IKZF3,BRCA1,EME1,RAD51C,BRIP1,RECQL5,TP53,PER1,ESCO1,RBBP8,POLI,RAD23A,BABAM1,XRCC1,ERCC2,ERCC1,LIG1,POLD1,NLRP2,XAB2,REV1,ERCC3,MCM6,RIF1,SMC6,GEN1,PMS1,INO80D,SMARCAL1,STK36,CUL3,SP140L,DNMT3A,MSH2,MSH6,RTEL1,TOP3B,CHEK2,XRCC6,POLQ,MCM2,TOPBP1,XPC,ATR,HLTF,HES1,TOP2B,SMARCC1,CDC25A,ATRIP,BAP1,PPP4R2,RAD18,UVSSA,SMARCA5,MND1,PLRG1,POLN,RFC1,HELQ,SMARCAD1,RAD50,POLK,MSH3,XRCC4,CCNH,REV3L,SHPRH,ESR1,MDC1,FANCE,ETV7,POLH,CDC5L,CD109,TTK,MMS22L,ORC5,EGFR,NAT1,WRN,NBN,RAD54B,SWI5,FANCG,SMC5,ANXA1,RMI1,CDC14B,POLA1,UBA1*

Table S3. Annotation of statistically significant variants identified by RIFT R package according to REVEAL and ClinVar databases.

SNP_detail	Hugo_Symbol	SNP_info	Race	Cancer	RSID	REVEL	CLIN_SIG
1:107600080-C/T	*PRMT6*	1:107600080	Asian	UCEC	NA	0.245	.
1:114449708-C/A	*DCLRE1B*	1:114449708	White	STAD	NA	0.506	.
1:114449708-C/A	*DCLRE1B*	1:114449708	Asian	STAD	NA	0.506	.
1:114449714-G/T	*DCLRE1B*	1:114449714	Asian	UCEC	NA	0.871	.
1:114449769-C/T	*DCLRE1B*	1:114449769	AA/B	SARC	NA	0.509	.
1:114449769-C/T	*DCLRE1B*	1:114449769	White	SARC	NA	0.509	.
1:114450790-A/C	*DCLRE1B*	1:114450790	White	LUAD	NA	0.52	.
1:114450790-A/C	*DCLRE1B*	1:114450790	AA/B	LUAD	NA	0.52	.
1:114454281-C/T	*DCLRE1B*	1:114454281	White	STAD	NA	0.332	.
1:115112680-C/A	*BCAS2*	1:115112680	Asian	UCEC	NA	0.448	.
1:115118260-G/T	*BCAS2*	1:115118260	White	UCEC	NA	0.538	.
1:117307144-G/A	*CD2*	1:117307144	White	STAD	NA	0.295	.
1:117307144-G/A	*CD2*	1:117307144	Asian	STAD	NA	0.295	.
1:117311199-C/T	*CD2*	1:117311199	White	UCEC	NA	0.056	.
1:11737658-G/A	*MAD2L2*	1:11737658	AA/B	LUAD	NA	0.694	.
1:11737658-G/A	*MAD2L2*	1:11737658	White	LUAD	NA	0.694	.
1:155234456-C/T	*CLK2*	1:155234456	White	UCEC	NA	0.463	.
1:155234529-G/A	*CLK2*	1:155234529	White	UCEC	NA	0.538	.
1:155235704-C/T	*CLK2*	1:155235704	White	CESC	NA	0.609	.
1:155235704-C/T	*CLK2*	1:155235704	AA/B	CESC	NA	0.609	.
1:161161052-C/G	*ADAMTS4*	1:161161052	AA/B	COAD	NA	0.333	.

1:161161052-C/G	*ADAMTS4*	1:161161052	White	COAD	NA	0.333	.
1:161161302-C/A	*ADAMTS4*	1:161161302	AA/B	OV	NA	0.344	.
1:161161302-C/A	*ADAMTS4*	1:161161302	White	OV	NA	0.344	.
1:161163164-C/T	*ADAMTS4*	1:161163164	AA/B	COAD	NA	0.436	.
1:161163164-C/T	*ADAMTS4*	1:161163164	White	COAD	NA	0.436	.
1:161163934-G/A	*ADAMTS4*	1:161163934	White	STAD	NA	0.209	.
1:161163934-G/A	*ADAMTS4*	1:161163934	Asian	STAD	NA	0.209	.
1:161166090-G/T	*ADAMTS4*	1:161166090	White	UCEC	NA	0.784	.
1:161166534-C/A	*ADAMTS4*	1:161166534	White	UCEC	NA	0.766	.
1:161166602-G/T	*ADAMTS4*	1:161166602	AA/B	LUSC	NA	0.582	.
1:161166602-G/T	*ADAMTS4*	1:161166602	White	LUSC	NA	0.582	.
1:161166670-G/A	*ADAMTS4*	1:161166670	White	COAD	NA	0.549	.
1:198676019-T/C	*PTPRC*	1:198676019	Asian	UCEC	NA	0.139	.
1:198677355-G/C	*PTPRC*	1:198677355	AA/B	LUAD	NA	0.246	.
1:198677355-G/C	*PTPRC*	1:198677355	White	LUAD	NA	0.246	.
1:198685876-A/C	*PTPRC*	1:198685876	White	STAD	NA	0.34	.
1:198685876-A/C	*PTPRC*	1:198685876	Asian	STAD	NA	0.34	.
1:198685877-C/T	*PTPRC*	1:198685877	White	UCEC	NA	0.291	.
1:198687382-G/T	*PTPRC*	1:198687382	White	STAD	NA	0.339	.
1:198687382-G/T	*PTPRC*	1:198687382	Asian	STAD	NA	0.339	.
1:198687388-T/C	*PTPRC*	1:198687388	White	STAD	NA	0.406	.
1:198700772-G/A	*PTPRC*	1:198700772	White	UCEC	NA	0.277	.
1:198700805-T/A	*PTPRC*	1:198700805	AA/B	LUAD	NA	0.585	.

1:198700805-T/A	*PTPRC*	1:198700805	White	LUAD	NA	0.585	.
1:198703333-A/T	*PTPRC*	1:198703333	AA/B	LUAD	NA	0.457	.
1:198703333-A/T	*PTPRC*	1:198703333	White	LUAD	NA	0.457	.
1:198703522-G/A	*PTPRC*	1:198703522	AA/B	COAD	NA	0.402	.
1:198703522-G/A	*PTPRC*	1:198703522	White	COAD	NA	0.402	.
1:198711070-G/C	*PTPRC*	1:198711070	White	BRCA	NA	0.447	.
1:198711070-G/C	*PTPRC*	1:198711070	Asian	BRCA	NA	0.447	.
1:198721444-G/T	*PTPRC*	1:198721444	White	COAD	NA	0.281	.
1:198721444-G/T	*PTPRC*	1:198721444	Asian	COAD	NA	0.281	.
1:198723441-C/T	*PTPRC*	1:198723441	Asian	UCEC	NA	0.297	.
1:198723454-C/T	*PTPRC*	1:198723454	White	COAD	NA	0.524	.
1:198725100-G/T	*PTPRC*	1:198725100	Asian	UCEC	NA	0.126	.
1:202302388-C/T	*UBE2T*	1:202302388	White	CESC	NA	0.232	.
1:202302447-G/T	*UBE2T*	1:202302447	Asian	ESCA	NA	0.557	.
1:202302447-G/T	*UBE2T*	1:202302447	White	ESCA	NA	0.557	.
1:226555974-G/A	*PARP1*	1:226555974	White	UCEC	NA	0.578	.
1:226566885-G/C	*PARP1*	1:226566885	Asian	BRCA	NA	0.516	.
1:226566885-G/C	*PARP1*	1:226566885	White	BRCA	NA	0.516	.
1:226567282-G/T	*PARP1*	1:226567282	White	COAD	NA	0.365	.
1:231483640-G/A	*SPRTN*	1:231483640	AA/B	BRCA	NA	0.632	.
1:231483640-G/A	*SPRTN*	1:231483640	AA/B	COAD	NA	0.632	.
1:231483640-G/A	*SPRTN*	1:231483640	White	BRCA	NA	0.632	.
1:231487101-C/T	*SPRTN*	1:231487101	AA/B	BRCA	NA	0.482	.

1:231487101-C/T	*SPRTN*	1:231487101	White	BRCA	NA	0.482	.
1:231488746-G/T	*SPRTN*	1:231488746	Asian	UCEC	NA	0.04	.
1:231488909-A/C	*SPRTN*	1:231488909	Asian	UCEC	NA	0.069	.
1:242020654-G/A	*EXO1*	1:242020654	Asian	UCEC	NA	0.449	.
1:242023969-G/T	*EXO1*	1:242023969	White	UCEC	NA	0.327	.
1:242024745-G/C	*EXO1*	1:242024745	White	BRCA	NA	0.731	.
1:242024745-G/C	*EXO1*	1:242024745	AA/B	BRCA	NA	0.731	.
1:242030154-G/C	*EXO1*	1:242030154	White	COAD	NA	0.086	.
1:242030154-G/C	*EXO1*	1:242030154	AA/B	COAD	NA	0.086	.
1:242030318-C/A	*EXO1*	1:242030318	White	COAD	NA	0.106	.
1:242030318-C/A	*EXO1*	1:242030318	AA/B	COAD	NA	0.106	.
1:242035389-G/T	*EXO1*	1:242035389	White	COAD	NA	0.117	.
1:28233489-C/A	*RPA2*	1:28233489	White	UCEC	NA	0.6	.
1:28233517-C/A	*RPA2*	1:28233517	White	BRCA	NA	0.062	.
1:28233517-C/A	*RPA2*	1:28233517	AA/B	BRCA	NA	0.062	.
1:45267334-A/G	*PLK3*	1:45267334	White	UCEC	NA	0.545	.
1:45268739-C/T	*PLK3*	1:45268739	White	COAD	NA	0.442	.
1:45268739-C/T	*PLK3*	1:45268739	AA/B	COAD	NA	0.442	.
1:45797719-C/A	*MUTYH*	1:45797719	White	LUAD	NA	0.064	.
1:45797719-C/A	*MUTYH*	1:45797719	AA/B	LUAD	NA	0.064	.
1:45798257-C/T	*MUTYH*	1:45798257	White	UCEC	NA	0.746	.
1:46715746-G/C	*RAD54L*	1:46715746	White	CESC	NA	0.408	.
1:46724394-A/G	*RAD54L*	1:46724394	AA/B	LUAD	NA	0.273	.

1:46724394-A/G	*RAD54L*	1:46724394	White	LUAD	NA	0.273	.
1:46739833-G/A	*RAD54L*	1:46739833	Asian	STAD	NA	0.902	.
1:46739833-G/A	*RAD54L*	1:46739833	White	STAD	NA	0.902	.
1:46740228-A/G	*RAD54L*	1:46740228	Asian	STAD	NA	0.75	.
1:46740228-A/G	*RAD54L*	1:46740228	White	STAD	NA	0.75	.
1:52841232-T/C	*ORC1*	1:52841232	AA/B	LUAD	NA	0.171	.
1:52841232-T/C	*ORC1*	1:52841232	White	LUAD	NA	0.171	.
1:52841244-G/A	*ORC1*	1:52841244	White	KIRC	NA	0.579	.
1:52841244-G/A	*ORC1*	1:52841244	AA/B	KIRC	NA	0.579	.
1:52849096-C/T	*ORC1*	1:52849096	AA/B	LUAD	NA	0.617	.
1:52849096-C/T	*ORC1*	1:52849096	White	LUAD	NA	0.617	.
1:52849519-C/T	*ORC1*	1:52849519	White	UCEC	NA	0.475	.
1:52861993-T/G	*ORC1*	1:52861993	White	UCEC	NA	0.429	.
1:62905615-A/C	*USP1*	1:62905615	Asian	COAD	NA	0.184	.
1:62905615-A/C	*USP1*	1:62905615	White	COAD	NA	0.184	.
1:62910485-C/G	*USP1*	1:62910485	AA/B	LUAD	NA	0.4	.
1:62910485-C/G	*USP1*	1:62910485	White	LUAD	NA	0.4	.
1:62910879-A/C	*USP1*	1:62910879	White	STAD	NA	0.164	.
1:62916312-A/C	*USP1*	1:62916312	White	COAD	NA	0.231	.
1:68152160-G/T	*GADD45A*	1:68152160	Asian	UCEC	NA	0.801	.
1:79094666-G/T	*IFI44L*	1:79094666	AA/B	LUAD	NA	0.039	.
1:79094666-G/T	*IFI44L*	1:79094666	White	LUAD	NA	0.039	.
1:79101116-G/C	*IFI44L*	1:79101116	White	LUAD	NA	0.137	.

1:79101116-G/C	*IFI44L*	1:79101116	AA/B	LUAD	NA	0.137	.
1:79102721-A/C	*IFI44L*	1:79102721	Asian	UCEC	NA	0.054	.
1:79107273-G/T	*IFI44L*	1:79107273	Asian	COAD	NA	0.198	.
1:79107273-G/T	*IFI44L*	1:79107273	White	COAD	NA	0.198	.
1:89726496-T/A	*GBP5*	1:89726496	White	LUAD	NA	0.145	.
1:89726496-T/A	*GBP5*	1:89726496	AA/B	LUAD	NA	0.145	.
1:89732073-G/T	*GBP5*	1:89732073	White	UCEC	NA	0.519	.
1:89732216-G/C	*GBP5*	1:89732216	White	THCA	NA	0.448	.
1:89732216-G/C	*GBP5*	1:89732216	AA/B	THCA	NA	0.448	.
1:89732235-C/T	*GBP5*	1:89732235	Asian	STAD	NA	0.569	.
1:89732235-C/T	*GBP5*	1:89732235	White	STAD	NA	0.569	.
1:91784878-A/C	*HFM1*	1:91784878	Asian	STAD	NA	0.103	.
1:91784878-A/C	*HFM1*	1:91784878	Asian	STAD	NA	0.541	.
1:91784878-A/C	*HFM1*	1:91784878	White	STAD	NA	0.103	.
1:91784878-A/C	*HFM1*	1:91784878	White	STAD	NA	0.541	.
1:91784910-C/G	*HFM1*	1:91784910	White	CESC	NA	0.75	.
1:91784910-C/G	*HFM1*	1:91784910	White	CESC	NA	0.437	.
1:91784910-C/G	*HFM1*	1:91784910	AA/B	CESC	NA	0.75	.
1:91784910-C/G	*HFM1*	1:91784910	AA/B	CESC	NA	0.437	.
1:91788701-T/G	*HFM1*	1:91788701	Asian	UCEC	NA	0.904	.
1:91788701-T/G	*HFM1*	1:91788701	Asian	UCEC	NA	0.43	.
1:91788737-C/A	*HFM1*	1:91788737	White	LUAD	NA	0.524	.
1:91788737-C/A	*HFM1*	1:91788737	White	LUAD	NA	0.146	.

1:91788737-C/A	*HFM1*	1:91788737	AA/B	LUAD	NA	0.524	.
1:91788737-C/A	*HFM1*	1:91788737	AA/B	LUAD	NA	0.146	.
1:91809039-T/A	*HFM1*	1:91809039	White	COAD	NA	0.6	.
1:91809039-T/A	*HFM1*	1:91809039	White	COAD	NA	0.734	.
1:91817190-G/C	*HFM1*	1:91817190	Asian	STAD	NA	0.613	.
1:91817190-G/C	*HFM1*	1:91817190	White	STAD	NA	0.613	.
1:91818152-T/C	*HFM1*	1:91818152	Asian	UCEC	NA	0.795	.
1:91818677-C/T	*HFM1*	1:91818677	AA/B	CESC	NA	0.757	.
1:91818677-C/T	*HFM1*	1:91818677	White	CESC	NA	0.757	.
1:91840948-G/T	*HFM1*	1:91840948	AA/B	LUAD	NA	0.23	.
1:91840948-G/T	*HFM1*	1:91840948	White	LUAD	NA	0.23	.
10:103339220-C/T	*POLL*	10:103339220	White	COAD	rs1225798009	0.87	.
10:103339220-C/T	*POLL*	10:103339220	AA/B	COAD	rs1225798009	0.87	.
10:112337595-C/G	*SMC3*	10:112337595	White	COAD	NA	0.479	.
10:112337595-C/G	*SMC3*	10:112337595	Asian	COAD	NA	0.479	.
10:112342324-C/A	*SMC3*	10:112342324	White	UCEC	NA	0.471	.
10:112349463-G/T	*SMC3*	10:112349463	White	COAD	NA	0.754	.
10:112352843-A/G	*SMC3*	10:112352843	White	STAD	NA	0.754	.
10:112352843-A/G	*SMC3*	10:112352843	Asian	STAD	NA	0.754	.
10:112360222-G/T	*SMC3*	10:112360222	Asian	UCEC	NA	0.743	.
10:115603939-C/A	*DCLRE1A*	10:115603939	Asian	UCEC	NA	0.968	.
10:115609027-C/A	*DCLRE1A*	10:115609027	White	KIRC	10:115609027_C/A	0.152	.

10:115609027-C/A	*DCLRE1A*	10:115609027	AA/B	KIRC	10:115609027_C/A	0.152	.
10:115610254-C/A	*DCLRE1A*	10:115610254	Asian	COAD	NA	0.18	.
10:115610254-C/A	*DCLRE1A*	10:115610254	White	COAD	NA	0.18	.
10:115610342-G/T	*DCLRE1A*	10:115610342	Asian	UCEC	NA	0.801	.
10:115612923-C/T	*DCLRE1A*	10:115612923	Asian	UCEC	rs1441187126	0.641	.
10:123719087-T/G	*NSMCE4A*	10:123719087	White	COAD	NA	0.091	.
10:123719087-T/G	*NSMCE4A*	10:123719087	Asian	COAD	NA	0.091	.
10:131557541-T/C	*MGMT*	10:131557541	White	STAD	NA	0.587	.
10:50679084-C/G	*ERCC6*	10:50679084	AA/B	LUAD	NA	0.503	.
10:50679084-C/G	*ERCC6*	10:50679084	White	LUAD	NA	0.503	.
10:50680495-T/A	*ERCC6*	10:50680495	White	STAD	NA	0.731	.
10:50680495-T/A	*ERCC6*	10:50680495	Asian	STAD	NA	0.731	.
10:50686456-G/A	*ERCC6*	10:50686456	AA/B	COAD	rs368188837	0.934	.
10:50686456-G/A	*ERCC6*	10:50686456	White	COAD	rs368188837	0.934	.
10:50691429-C/T	*ERCC6*	10:50691429	White	COAD	NA	0.905	.
10:50713973-C/T	*ERCC6*	10:50713973	White	COAD	rs749392467	0.569	.
10:51040912-G/T	*PARG*	10:51040912	Asian	UCEC	NA	0.206	.
10:51069666-T/C	*PARG*	10:51069666	AA/B	COAD	NA	0.3	.
10:51069666-T/C	*PARG*	10:51069666	White	COAD	NA	0.3	.
10:51087793-G/T	*PARG*	10:51087793	Asian	UCEC	NA	0.526	.
10:59955929-C/G	*IPMK*	10:59955929	AA/B	LUAD	NA	0.39	.
10:59955929-C/G	*IPMK*	10:59955929	White	LUAD	NA	0.39	.

10:59955943-C/T	*IPMK*	10:59955943	White	UCEC	rs201911489	0.207	.
10:59986839-T/C	*IPMK*	10:59986839	White	UCEC	rs778350776	0.523	.
10:70182635-T/G	*DNA2*	10:70182635	White	STAD	NA	0.743	.
10:70182635-T/G	*DNA2*	10:70182635	White	STAD	NA	0.9	.
10:70190369-T/C	*DNA2*	10:70190369	White	BRCA	NA	0.947	.
10:70190369-T/C	*DNA2*	10:70190369	AA/B	BRCA	NA	0.947	.
10:70202736-C/A	*DNA2*	10:70202736	AA/B	LUAD	NA	0.832	.
10:70202736-C/A	*DNA2*	10:70202736	White	LUAD	NA	0.832	.
10:70204814-C/T	*DNA2*	10:70204814	White	COAD	NA	0.643	.
10:70209804-G/T	*DNA2*	10:70209804	Asian	UCEC	NA	0.794	.
10:70209946-C/T	*DNA2*	10:70209946	White	STAD	rs1166986642	0.709	.
10:70209946-C/T	*DNA2*	10:70209946	Asian	STAD	rs1166986642	0.709	.
10:70227993-T/A	*DNA2*	10:70227993	White	COAD	NA	0.647	.
10:89624242-A/G	*PTEN*	10:89624242	White	STAD	NA	0.89	.
10:89624242-A/G	*PTEN*	10:89624242	Asian	STAD	NA	0.89	.
10:89624267-G/T	*PTEN*	10:89624267	White	UCEC	NA	0.912	.
10:89624271-A/C	*PTEN*	10:89624271	White	COAD	NA	0.799	.
10:89624271-A/C	*PTEN*	10:89624271	AA/B	COAD	NA	0.799	.
10:89624297-A/T	*PTEN*	10:89624297	Asian	STAD	NA	0.994	.
10:89624297-A/T	*PTEN*	10:89624297	White	STAD	NA	0.994	.
10:89653785-T/G	*PTEN*	10:89653785	White	STAD	NA	0.98	.
10:89653808-G/A	*PTEN*	10:89653808	White	UCEC	NA	0.995	.
10:89692790-G/T	*PTEN*	10:89692790	White	UCEC	NA	0.96	.

10:89692890-A/C	*PTEN*	10:89692890	White	UCEC	NA	0.914	.
10:89692892-G/T	*PTEN*	10:89692892	White	UCEC	NA	0.889	.
10:89692893-C/G	*PTEN*	10:89692893	White	BRCA	NA	0.878	.
10:89692893-C/G	*PTEN*	10:89692893	AA/B	BRCA	NA	0.878	.
10:89692902-G/A	*PTEN*	10:89692902	White	LUSC	NA	0.94	pathogenic
10:89692902-G/A	*PTEN*	10:89692902	AA/B	LUSC	NA	0.94	pathogenic
10:89692904-C/G	*PTEN*	10:89692904	White	UCEC	NA	0.962	pathogenic
10:89692904-C/G	*PTEN*	10:89692904	AA/B	UCEC	NA	0.962	pathogenic
10:89692905-G/A	*PTEN*	10:89692905	White	COAD	NA	0.976	likely_pathogenic,pathogenic,pathogenic
10:89692905-G/A	PTEN	10:89692905	Asian	UCEC	NA	0.976	likely_pathogenic,pathogenic,pathogenic
10:89692905-G/A	PTEN	10:89692905	AA/B	STAD	NA	0.976	likely_pathogenic,pathogenic,pathogenic
10:89692905-G/C	PTEN	10:89692905	White	UCEC	NA	0.984	likely_pathogenic,pathogenic,pathogenic
10:89692905-G/T	PTEN	10:89692905	White	UCEC	NA	0.987	likely_pathogenic,pathogenic,pathogenic

10:89692911-G/A	PTEN	10:89692911	White	UCEC	NA	0.991	uncertain_significance,pathogenic
10:89692920-T/A	PTEN	10:89692920	White	BRCA	NA	0.99	.
10:89692920-T/A	PTEN	10:89692920	AA/B	BRCA	NA	0.99	.
10:89692922-T/C	PTEN	10:89692922	AA/B	UCEC	NA	0.991	.
10:89692922-T/C	PTEN	10:89692922	White	UCEC	NA	0.991	.
10:89692923-G/T	PTEN	10:89692923	Asian	UCEC	NA	0.966	.
10:89692926-C/T	PTEN	10:89692926	White	UCEC	NA	0.926	.
10:89692971-T/C	*PTEN*	10:89692971	White	UCEC	NA	0.951	.
10:89711899-C/T	*PTEN*	10:89711899	AA/B	UCEC	NA	0.972	pathogenic
10:89711899-C/T	*PTEN*	10:89711899	AA/B	LGG	NA	0.972	pathogenic
10:89711899-C/T	*PTEN*	10:89711899	White	COAD	NA	0.972	pathogenic
10:89711899-C/T	*PTEN*	10:89711899	White	UCEC	NA	0.972	pathogenic
10:89711899-C/T	*PTEN*	10:89711899	White	LGG	NA	0.972	pathogenic
10:89711900-G/A	*PTEN*	10:89711900	AA/B	UCEC	rs121913294	0.949	likely_pathogenic
10:89711900-G/A	*PTEN*	10:89711900	White	UCEC	rs121913294	0.949	likely_pathogenic
10:89717625-T/G	*PTEN*	10:89717625	White	BRCA	NA	0.913	.
10:89717625-T/G	*PTEN*	10:89717625	AA/B	BRCA	NA	0.913	.
10:89717721-T/A	*PTEN*	10:89717721	Asian	UCEC	NA	0.96	.
10:89717729-G/T	*PTEN*	10:89717729	Asian	UCEC	NA	0.973	.
10:89720813-A/G	*PTEN*	10:89720813	White	LGG	NA	0.877	.

10:89720813-A/G	*PTEN*	10:89720813	AA/B	LGG	NA	0.877	.
10:96348061-G/A	*HELLS*	10:96348061	White	UCEC	rs1333262815	0.302	.
10:96350486-C/T	*HELLS*	10:96350486	White	UCEC	NA	0.665	.
10:96353359-A/C	*HELLS*	10:96353359	AA/B	BRCA	NA	0.733	.
10:96353359-A/C	*HELLS*	10:96353359	White	BRCA	NA	0.733	.
10:96356854-G/A	*HELLS*	10:96356854	White	UCEC	NA	0.659	.
10:96361331-C/G	*HELLS*	10:96361331	White	UCEC	NA	0.626	.
10:98084055-G/T	*DNTT*	10:98084055	White	UCEC	NA	0.265	.
10:98088496-G/T	*DNTT*	10:98088496	White	STAD	NA	0.568	.
10:98088496-G/T	*DNTT*	10:98088496	Asian	STAD	NA	0.568	.
10:98092286-G/A	*DNTT*	10:98092286	White	UCEC	rs142389547	0.824	.
10:98092321-G/A	*DNTT*	10:98092321	White	STAD	NA	0.371	.
10:98092321-G/A	*DNTT*	10:98092321	Asian	STAD	NA	0.371	.
10:98092349-C/T	*DNTT*	10:98092349	AA/B	LUAD	NA	0.616	.
10:98092349-C/T	*DNTT*	10:98092349	White	LUAD	NA	0.616	.
10:99225597-G/A	*MMS19*	10:99225597	White	STAD	NA	0.557	.
10:99225597-G/A	*MMS19*	10:99225597	White	STAD	NA	0.354	.
10:99228834-C/T	*MMS19*	10:99228834	White	CESC	NA	0.297	.
10:99228834-C/T	*MMS19*	10:99228834	AA/B	CESC	NA	0.297	.
11:107920618-G/A	*CUL5*	11:107920618	White	UCEC	rs775692877	0.266	.
11:107920713-T/C	*CUL5*	11:107920713	White	STAD	NA	0.583	.
11:107920713-T/C	*CUL5*	11:107920713	Asian	STAD	NA	0.583	.
11:107925500-T/C	*CUL5*	11:107925500	White	COAD	NA	0.672	.

11:107960288-C/T	*CUL5*	11:107960288	White	UCEC	NA	0.873	.
11:107965125-G/A	*CUL5*	11:107965125	White	LUAD	NA	0.861	.
11:107965125-G/A	*CUL5*	11:107965125	AA/B	LUAD	NA	0.861	.
11:107968424-C/T	*CUL5*	11:107968424	AA/B	CESC	NA	0.607	.
11:107968424-C/T	*CUL5*	11:107968424	White	CESC	NA	0.607	.
11:107974999-A/C	*CUL5*	11:107974999	White	COAD	NA	0.287	.
11:108098563-C/T	*ATM*	11:108098563	Asian	UCEC	rs3218684	0.095	uncertain_significance
11:108099998-G/T	*ATM*	11:108099998	White	COAD	NA	0.157	.
11:108117798-C/T	*ATM*	11:108117798	Asian	COAD	rs138398778	0.36	uncertain_significance
11:108119696-C/G	*ATM*	11:108119696	AA/B	LGG	NA	0.16	.
11:108119696-C/G	*ATM*	11:108119696	White	LGG	NA	0.16	.
11:108124699-T/C	*ATM*	11:108124699	Asian	UCEC	NA	0.626	.
11:108139224-C/T	*ATM*	11:108139224	White	KIRC	NA	0.305	.
11:108139224-C/T	*ATM*	11:108139224	AA/B	KIRC	NA	0.305	.
11:108141997-C/T	*ATM*	11:108141997	White	STAD	rs587780619	0.739	uncertain_significance
11:108141997-C/T	*ATM*	11:108141997	Asian	STAD	rs587780619	0.739	uncertain_significance
11:108153601-C/A	*ATM*	11:108153601	Asian	UCEC	NA	0.562	.
11:108153601-C/A	*ATM*	11:108153601	White	COAD	NA	0.562	.
11:108160347-C/A	*ATM*	11:108160347	Asian	UCEC	NA	0.062	.

11:108160347-C/A	*ATM*	11:108160347	Asian	UCEC	NA	0.3	.
11:108163396-A/G	*ATM*	11:108163396	Asian	UCEC	NA	0.659	.
11:108164059-A/G	*ATM*	11:108164059	White	UCEC	rs779718362	0.195	.
11:108170482-T/G	*ATM*	11:108170482	Asian	UCEC	NA	0.286	.
11:108186752-A/G	*ATM*	11:108186752	Asian	UCEC	NA	0.477	.
11:108192065-G/C	*ATM*	11:108192065	AA/B	BLCA	NA	0.666	.
11:108200961-G/A	*ATM*	11:108200961	White	BRCA	rs587782310	0.466	uncertain_significance
11:108200961-G/A	*ATM*	11:108200961	AA/B	BRCA	rs587782310	0.466	uncertain_significance
11:108205734-A/G	*ATM*	11:108205734	Asian	UCEC	rs1292192410	0.718	.
11:108205742-T/G	*ATM*	11:108205742	White	STAD	NA	0.937	.
11:108205742-T/G	*ATM*	11:108205742	Asian	STAD	NA	0.937	.
11:108206584-C/A	*ATM*	11:108206584	Asian	UCEC	NA	0.692	uncertain_significance
11:108214018-C/A	*ATM*	11:108214018	White	UCEC	NA	0.598	.
11:108216545-C/T	*ATM*	11:108216545	White	LUAD	rs587779872	0.83	likely_pathogenic,pathogenic
11:108216545-C/T	*ATM*	11:108216545	AA/B	LUAD	rs587779872	0.83	likely_pathogenic,pathogenic
11:108218017-C/A	*ATM*	11:108218017	Asian	UCEC	NA	0.484	uncertain_significance
11:108225584-C/A	*ATM*	11:108225584	White	UCEC	NA	0.739	.

11:125514032-G/T	*CHEK1*	11:125514032	AA/B	LUAD	NA	0.282	.
11:125514032-G/T	*CHEK1*	11:125514032	White	LUAD	NA	0.282	.
11:125514441-G/A	*CHEK1*	11:125514441	White	STAD	NA	0.725	.
11:125514441-G/A	*CHEK1*	11:125514441	Asian	STAD	NA	0.725	.
11:125525174-G/T	*CHEK1*	11:125525174	White	COAD	NA	0.688	.
11:18382202-C/T	*GTF2H1*	11:18382202	White	UCEC	rs999720593	0.268	.
11:22646875-G/T	*FANCF*	11:22646875	White	UCEC	NA	0.06	.
11:22647053-C/A	*FANCF*	11:22647053	White	UCEC	NA	0.065	.
11:43904237-G/A	*ALKBH3*	11:43904237	Asian	ESCA	NA	0.385	.
11:43904237-G/A	*ALKBH3*	11:43904237	White	ESCA	NA	0.385	.
11:43908203-G/T	*ALKBH3*	11:43908203	White	KIRP	11:43908203_G/T	0.245	.
11:43908203-G/T	*ALKBH3*	11:43908203	AA/B	KIRP	11:43908203_G/T	0.245	.
11:43911298-T/C	*ALKBH3*	11:43911298	White	UCEC	rs542847003	0.192	.
11:43940636-G/T	*ALKBH3*	11:43940636	AA/B	LUAD	NA	0.158	.
11:43940636-G/T	*ALKBH3*	11:43940636	AA/B	LUAD	NA	0.081	.
11:43940636-G/T	*ALKBH3*	11:43940636	White	LUAD	NA	0.158	.
11:43940636-G/T	*ALKBH3*	11:43940636	White	LUAD	NA	0.081	.
11:43940667-C/T	*ALKBH3*	11:43940667	White	UCEC	rs745683816	0.125	.
11:46889641-G/T	*LRP4*	11:46889641	White	LUAD	NA	0.442	.
11:46889641-G/T	*LRP4*	11:46889641	AA/B	LUAD	NA	0.442	.
11:46897369-C/T	*LRP4*	11:46897369	White	OV	rs760528582	0.79	.

11:46897369-C/T	*LRP4*	11:46897369	AA/B	OV	rs760528582	0.79	.
11:46905450-G/A	*LRP4*	11:46905450	White	UCEC	rs1216367812	0.91	.
11:46905469-C/A	*LRP4*	11:46905469	White	LUAD	NA	0.875	.
11:46905469-C/A	*LRP4*	11:46905469	AA/B	LUAD	NA	0.875	.
11:46911003-G/A	*LRP4*	11:46911003	White	STAD	NA	0.898	.
11:46911003-G/A	*LRP4*	11:46911003	Asian	STAD	NA	0.898	.
11:46911620-G/A	*LRP4*	11:46911620	White	UCEC	NA	0.912	.
11:46911654-C/T	*LRP4*	11:46911654	White	COAD	NA	0.879	.
11:46911848-C/T	*LRP4*	11:46911848	White	UCEC	NA	0.89	.
11:46911897-C/T	*LRP4*	11:46911897	White	UCEC	NA	0.906	.
11:46916184-C/T	*LRP4*	11:46916184	White	COAD	NA	0.937	.
11:46916184-C/T	*LRP4*	11:46916184	Asian	COAD	NA	0.937	.
11:46916274-T/C	*LRP4*	11:46916274	White	LUAD	NA	0.551	.
11:46916274-T/C	*LRP4*	11:46916274	AA/B	LUAD	NA	0.551	.
11:46916347-C/T	*LRP4*	11:46916347	White	UCEC	rs1023257694	0.658	.
11:47254399-T/G	*DDB2*	11:47254399	Asian	UCEC	NA	0.61	.
11:47256959-T/C	*DDB2*	11:47256959	White	UCEC	NA	0.501	.
11:57095242-C/A	*SSRP1*	11:57095242	White	UCEC	NA	0.888	.
11:57099148-A/G	*SSRP1*	11:57099148	White	STAD	NA	0.749	.
11:57099148-A/G	*SSRP1*	11:57099148	Asian	STAD	NA	0.749	.
11:57099296-G/A	*SSRP1*	11:57099296	White	STAD	NA	0.404	.
11:57099948-G/A	*SSRP1*	11:57099948	White	COAD	NA	0.717	.
11:57099948-G/A	*SSRP1*	11:57099948	AA/B	COAD	NA	0.717	.

11:57100226-T/C	*SSRP1*	11:57100226	Asian	STAD	NA	0.757	.
11:57100226-T/C	*SSRP1*	11:57100226	White	STAD	NA	0.757	.
11:57100444-C/T	*SSRP1*	11:57100444	White	UCEC	NA	0.318	.
11:57100468-G/A	*SSRP1*	11:57100468	AA/B	LUSC	NA	0.645	.
11:57100468-G/A	*SSRP1*	11:57100468	White	LUSC	NA	0.645	.
11:57102013-C/G	*SSRP1*	11:57102013	White	BRCA	NA	0.833	.
11:57102013-C/G	*SSRP1*	11:57102013	AA/B	BRCA	NA	0.833	.
11:60665619-C/T	*PRPF19*	11:60665619	White	STAD	NA	0.802	.
11:60665647-C/A	*PRPF19*	11:60665647	AA/B	LUAD	NA	0.891	.
11:60665647-C/A	*PRPF19*	11:60665647	White	LUAD	NA	0.891	.
11:60666022-C/A	*PRPF19*	11:60666022	Asian	UCEC	NA	0.855	.
11:60666653-C/T	*PRPF19*	11:60666653	White	STAD	rs1396955598	0.576	.
11:60666653-C/T	*PRPF19*	11:60666653	Asian	STAD	rs1396955598	0.576	.
11:61071504-G/A	*DDB1*	11:61071504	White	STAD	NA	0.638	.
11:61071504-G/A	*DDB1*	11:61071504	Asian	STAD	NA	0.638	.
11:61091539-C/T	*DDB1*	11:61091539	White	STAD	NA	0.946	.
11:61091539-C/T	*DDB1*	11:61091539	Asian	STAD	NA	0.946	.
11:61094352-C/T	*DDB1*	11:61094352	White	UCEC	NA	0.37	.
11:61096912-G/A	*DDB1*	11:61096912	AA/B	COAD	NA	0.507	.
11:61096912-G/A	*DDB1*	11:61096912	White	COAD	NA	0.507	.
11:61563308-G/A	*FEN1*	11:61563308	White	STAD	rs778852873	0.918	.
11:61563308-G/A	*FEN1*	11:61563308	Asian	STAD	rs778852873	0.918	.
11:65482045-A/T	*KAT5*	11:65482045	White	UCEC	NA	0.522	.

11:65631344-T/G	*MUS81*	11:65631344	White	STAD	rs138527502	0.471	.
11:65631344-T/G	*MUS81*	11:65631344	White	STAD	rs138527502	0.376	.
11:65631344-T/G	*MUS81*	11:65631344	Asian	STAD	rs138527502	0.471	.
11:65631344-T/G	*MUS81*	11:65631344	Asian	STAD	rs138527502	0.376	.
11:67163792-C/A	*RAD9A*	11:67163792	White	UCEC	NA	0.214	.
11:74340322-G/C	*POLD3*	11:74340322	AA/B	BRCA	NA	0.275	.
11:74340322-G/C	*POLD3*	11:74340322	AA/B	BRCA	NA	0.186	.
11:74340322-G/C	*POLD3*	11:74340322	White	BRCA	NA	0.275	.
11:74340322-G/C	*POLD3*	11:74340322	White	BRCA	NA	0.186	.
11:74340348-C/G	*POLD3*	11:74340348	White	CESC	NA	0.28	.
11:74340348-C/G	*POLD3*	11:74340348	White	CESC	NA	0.144	.
11:74340348-C/G	*POLD3*	11:74340348	AA/B	CESC	NA	0.28	.
11:74340348-C/G	*POLD3*	11:74340348	AA/B	CESC	NA	0.144	.
11:74521240-A/C	*RNF169*	11:74521240	White	STAD	NA	0.097	.
11:74546822-C/A	*RNF169*	11:74546822	White	THYM	NA	0.325	.
11:74546822-C/A	*RNF169*	11:74546822	Asian	THYM	NA	0.325	.
11:74546844-G/A	*RNF169*	11:74546844	Asian	ESCA	NA	0.826	.
11:74546844-G/A	*RNF169*	11:74546844	White	ESCA	NA	0.826	.
11:74546883-G/A	*RNF169*	11:74546883	White	LUAD	rs1309338384	0.339	.
11:74546883-G/A	*RNF169*	11:74546883	AA/B	LUAD	rs1309338384	0.339	.
11:9606861-G/A	*WEE1*	11:9606861	Asian	UCEC	NA	0.217	.
11:9607061-A/C	*WEE1*	11:9607061	White	BRCA	NA	0.92	.
11:9607061-A/C	*WEE1*	11:9607061	AA/B	BRCA	NA	0.92	.

11:9608313-A/G	*WEE1*	11:9608313	Asian	STAD	NA	0.566	.
11:9608313-A/G	*WEE1*	11:9608313	White	STAD	NA	0.566	.
11:9610133-G/T	*WEE1*	11:9610133	AA/B	SARC	NA	0.654	.
11:9610133-G/T	*WEE1*	11:9610133	White	SARC	NA	0.654	.
12:1023144-G/A	*RAD52*	12:1023144	White	UCEC	NA	0.146	.
12:1023149-C/A	*RAD52*	12:1023149	White	UCEC	NA	0.102	.
12:102542015-G/T	*PARPBP*	12:102542015	White	KIRP	NA	0.406	.
12:102542015-G/T	*PARPBP*	12:102542015	AA/B	KIRP	NA	0.406	.
12:102558273-A/C	*PARPBP*	12:102558273	White	COAD	NA	0.354	.
12:102559514-C/T	*PARPBP*	12:102559514	Asian	STAD	rs775852790	0.188	.
12:102559514-C/T	*PARPBP*	12:102559514	White	STAD	rs775852790	0.188	.
12:102559591-A/C	*PARPBP*	12:102559591	White	COAD	NA	0.4	.
12:102559591-A/C	*PARPBP*	12:102559591	Asian	COAD	NA	0.4	.
12:102569284-C/T	*PARPBP*	12:102569284	Asian	BRCA	NA	0.392	.
12:102569284-C/T	*PARPBP*	12:102569284	White	BRCA	NA	0.392	.
12:102569382-A/C	*PARPBP*	12:102569382	White	COAD	NA	0.104	.
12:104373818-G/A	*TDG*	12:104373818	Asian	STAD	rs149084574	0.463	.
12:104373818-G/A	*TDG*	12:104373818	White	STAD	rs149084574	0.463	.
12:104376688-C/T	*TDG*	12:104376688	Asian	COAD	rs745524841	0.3	.
12:104376688-C/T	*TDG*	12:104376688	Asian	COAD	rs745524841	0.482	.
12:104376688-C/T	*TDG*	12:104376688	White	COAD	rs745524841	0.3	.
12:104376688-C/T	*TDG*	12:104376688	White	COAD	rs745524841	0.482	.
12:104697876-G/A	*EID3*	12:104697876	AA/B	LIHC	NA	0.403	.

12:104697950-G/T	*EID3*	12:104697950	White	LUAD	NA	0.565	.
12:104697950-G/T	*EID3*	12:104697950	AA/B	LUAD	NA	0.565	.
12:104697977-G/A	*EID3*	12:104697977	White	STAD	NA	0.618	.
12:104698101-A/T	*EID3*	12:104698101	Asian	STAD	NA	0.103	.
12:104698101-A/T	*EID3*	12:104698101	White	STAD	NA	0.103	.
12:104698212-C/T	*EID3*	12:104698212	White	STAD	NA	0.171	.
12:104698212-C/T	*EID3*	12:104698212	Asian	STAD	NA	0.171	.
12:104698483-G/T	*EID3*	12:104698483	White	UCEC	NA	0.642	.
12:104698527-G/T	*EID3*	12:104698527	Asian	UCEC	NA	0.287	.
12:109526219-C/G	*ALKBH2*	12:109526219	White	BRCA	NA	0.737	.
12:109526219-C/G	*ALKBH2*	12:109526219	White	BRCA	NA	0.143	.
12:109526219-C/G	*ALKBH2*	12:109526219	Asian	BRCA	NA	0.737	.
12:109526219-C/G	*ALKBH2*	12:109526219	Asian	BRCA	NA	0.143	.
12:109539758-C/T	*UNG*	12:109539758	AA/B	CESC	NA	0.548	.
12:109539758-C/T	*UNG*	12:109539758	White	CESC	NA	0.548	.
12:109539760-C/A	*UNG*	12:109539760	White	STAD	rs376918741	0.477	.
12:109539760-C/A	*UNG*	12:109539760	Asian	STAD	rs376918741	0.477	.
12:109541373-T/G	*UNG*	12:109541373	AA/B	KIRC	NA	0.763	.
12:109541373-T/G	*UNG*	12:109541373	AA/B	KIRC	NA	0.714	.
12:109541373-T/G	*UNG*	12:109541373	White	KIRC	NA	0.763	.
12:109541373-T/G	*UNG*	12:109541373	White	KIRC	NA	0.714	.
12:110940182-G/A	*RAD9B*	12:110940182	White	UCEC	NA	0.049	.
12:110943422-C/A	*RAD9B*	12:110943422	White	UCEC	NA	0.187	.

12:110944402-A/G	*RAD9B*	12:110944402	White	LUAD	NA	0.438	.
12:110944402-A/G	*RAD9B*	12:110944402	AA/B	LUAD	NA	0.438	.
12:110950593-G/T	*RAD9B*	12:110950593	White	UCEC	rs753006923	0.266	.
12:110956716-T/G	*RAD9B*	12:110956716	AA/B	KIRC	NA	0.327	.
12:110956716-T/G	*RAD9B*	12:110956716	White	KIRC	NA	0.327	.
12:110960215-A/C	*RAD9B*	12:110960215	White	COAD	NA	0.136	.
12:118468998-C/T	*RFC5*	12:118468998	White	BRCA	NA	0.265	.
12:118468998-C/T	*RFC5*	12:118468998	Asian	BRCA	NA	0.265	.
12:124135594-G/T	*GTF2H3*	12:124135594	White	SARC	rs1191432224	0.718	.
12:124135594-G/T	*GTF2H3*	12:124135594	AA/B	SARC	rs1191432224	0.718	.
12:133208906-A/G	*POLE*	12:133208906	Asian	UCEC	NA	0.691	.
12:133209378-T/C	*POLE*	12:133209378	White	STAD	NA	0.615	.
12:133215787-G/A	*POLE*	12:133215787	White	COAD	rs762000608	0.34	.
12:133215787-G/A	*POLE*	12:133215787	White	UCEC	rs762000608	0.34	.
12:133218372-C/T	*POLE*	12:133218372	White	STAD	rs753105796	0.245	.
12:133218372-C/T	*POLE*	12:133218372	Asian	STAD	rs753105796	0.245	.
12:133219468-G/A	*POLE*	12:133219468	White	COAD	rs768741587	0.373	.
12:133240978-G/A	*POLE*	12:133240978	Asian	STAD	NA	0.528	.
12:133240978-G/A	*POLE*	12:133240978	White	STAD	NA	0.528	.
12:133249847-G/A	*POLE*	12:133249847	White	UCEC	NA	0.418	.
12:133250189-A/T	*POLE*	12:133250189	Asian	UCEC	NA	0.621	.
12:133250250-G/T	*POLE*	12:133250250	Asian	UCEC	NA	0.593	risk_factor
12:133250289-C/A	*POLE*	12:133250289	White	COAD	NA	0.457	.

12:133250289-C/A	*POLE*	12:133250289	White	STAD	NA	0.457	.
12:133250289-C/A	*POLE*	12:133250289	Asian	UCEC	NA	0.457	.
12:133253151-G/A	*POLE*	12:133253151	White	UCEC	NA	0.799	.
12:133253184-G/A	*POLE*	12:133253184	White	STAD	NA	0.812	.
12:133253184-G/A	*POLE*	12:133253184	Asian	STAD	NA	0.812	.
12:133253184-G/C	*POLE*	12:133253184	Asian	UCEC	NA	0.837	.
12:133253184-G/C	*POLE*	12:133253184	Asian	COAD	NA	0.837	.
12:133253184-G/C	*POLE*	12:133253184	White	COAD	NA	0.837	.
12:21644542-A/G	*RECQL*	12:21644542	White	STAD	NA	0.455	.
12:42853756-G/T	*PRICKLE1*	12:42853756	White	STAD	NA	0.743	.
12:42853756-G/T	*PRICKLE1*	12:42853756	Asian	STAD	NA	0.743	.
12:42854131-C/T	*PRICKLE1*	12:42854131	White	UCEC	NA	0.638	.
12:42854422-G/T	*PRICKLE1*	12:42854422	White	COAD	NA	0.466	.
12:42858731-C/T	*PRICKLE1*	12:42858731	Asian	STAD	NA	0.143	.
12:42858731-C/T	*PRICKLE1*	12:42858731	White	STAD	NA	0.143	.
12:42858865-G/A	*PRICKLE1*	12:42858865	White	STAD	NA	0.439	.
12:42858908-C/T	*PRICKLE1*	12:42858908	White	SARC	NA	0.26	.
12:42858908-C/T	*PRICKLE1*	12:42858908	AA/B	SARC	NA	0.26	.
12:42862485-A/C	*PRICKLE1*	12:42862485	White	KIRC	NA	0.611	.
12:42862485-A/C	*PRICKLE1*	12:42862485	AA/B	KIRC	NA	0.611	.
12:42864101-C/G	*PRICKLE1*	12:42864101	AA/B	BRCA	NA	0.804	.
12:42864101-C/G	*PRICKLE1*	12:42864101	White	BRCA	NA	0.804	.
12:54577441-C/A	*SMUG1*	12:54577441	AA/B	KIRP	NA	0.699	.

12:54577441-C/A	*SMUG1*	12:54577441	White	KIRP	NA	0.699	.
12:54577576-G/T	*SMUG1*	12:54577576	White	COAD	NA	0.281	.
12:54577576-G/T	*SMUG1*	12:54577576	AA/B	COAD	NA	0.281	.
12:56619478-G/C	*NABP2*	12:56619478	White	LUAD	NA	0.85	.
12:56619478-G/C	*NABP2*	12:56619478	AA/B	LUAD	NA	0.85	.
12:56815232-C/T	*TIMELESS*	12:56815232	White	UCEC	rs768425224	0.149	.
12:56815708-C/T	*TIMELESS*	12:56815708	White	COAD	NA	0.183	.
12:56815713-C/A	*TIMELESS*	12:56815713	White	LUAD	NA	0.449	.
12:56815713-C/A	*TIMELESS*	12:56815713	AA/B	LUAD	NA	0.449	.
12:56815747-G/A	*TIMELESS*	12:56815747	Asian	UCEC	rs758367557	0.539	.
12:56818547-G/A	*TIMELESS*	12:56818547	Asian	UCEC	rs780416093	0.391	.
12:56822112-G/A	*TIMELESS*	12:56822112	Asian	STAD	rs550048328	0.156	.
12:56822112-G/A	*TIMELESS*	12:56822112	White	STAD	rs550048328	0.156	.
12:56822755-G/A	*TIMELESS*	12:56822755	Asian	STAD	rs765264874	0.345	.
12:56822755-G/A	*TIMELESS*	12:56822755	White	STAD	rs765264874	0.345	.
12:56824055-T/C	*TIMELESS*	12:56824055	White	LUSC	NA	0.46	.
12:56824055-T/C	*TIMELESS*	12:56824055	AA/B	LUSC	NA	0.46	.
12:56827333-C/G	*TIMELESS*	12:56827333	AA/B	OV	NA	0.304	.
12:56827333-C/G	*TIMELESS*	12:56827333	White	OV	NA	0.304	.
12:56827937-C/T	*TIMELESS*	12:56827937	Asian	ESCA	rs1467514517	0.269	.
12:56827937-C/T	*TIMELESS*	12:56827937	White	ESCA	rs1467514517	0.269	.
12:76444373-G/A	*NAP1L1*	12:76444373	Asian	THCA	NA	0.38	.
12:76444373-G/A	*NAP1L1*	12:76444373	White	THCA	NA	0.38	.

12:76447003-A/C	*NAP1L1*	12:76447003	White	COAD	NA	0.697	.
12:76447595-G/A	*NAP1L1*	12:76447595	Asian	UCEC	NA	0.185	.
12:8800725-A/T	*MFAP5*	12:8800725	AA/B	LIHC	NA	0.655	.
12:8800752-G/A	*MFAP5*	12:8800752	White	UCEC	rs779463853	0.253	.
12:8803134-G/A	*MFAP5*	12:8803134	White	COAD	rs753225796	0.568	.
12:8803134-G/A	*MFAP5*	12:8803134	White	COAD	rs753225796	0.173	.
12:8808382-T/G	*MFAP5*	12:8808382	White	COAD	NA	0.113	.
12:8808397-A/C	*MFAP5*	12:8808397	Asian	UCEC	NA	0.218	.
12:8808397-A/C	*MFAP5*	12:8808397	Asian	UCEC	NA	0.226	.
12:8813485-G/T	*MFAP5*	12:8813485	AA/B	LUAD	NA	0.04	.
12:8813485-G/T	*MFAP5*	12:8813485	AA/B	LUAD	NA	0.011	.
12:8813485-G/T	*MFAP5*	12:8813485	White	LUAD	NA	0.04	.
12:8813485-G/T	*MFAP5*	12:8813485	White	LUAD	NA	0.011	.
13:103498683-G/A	*ERCC5*	13:103498683	White	COAD	NA	0.242	.
13:103525633-G/T	*ERCC5*	13:103525633	AA/B	OV	NA	0.939	pathogenic
13:103525633-G/T	*ERCC5*	13:103525633	White	OV	NA	0.939	pathogenic
13:103527667-G/A	*ERCC5*	13:103527667	White	UCEC	rs377390651	0.5	.
13:108861290-T/C	*LIG4*	13:108861290	Asian	UCEC	NA	0.321	.
13:108861812-A/C	*LIG4*	13:108861812	Asian	UCEC	NA	0.625	.
13:108862104-G/A	*LIG4*	13:108862104	White	UCEC	rs375284799	0.666	.
13:113873275-G/C	*CUL4A*	13:113873275	White	BRCA	NA	0.883	.
13:113873275-G/C	*CUL4A*	13:113873275	AA/B	BRCA	NA	0.883	.
13:113899329-C/T	*CUL4A*	13:113899329	White	STAD	NA	0.625	.

13:113899329-C/T	*CUL4A*	13:113899329	Asian	STAD	NA	0.625	.
13:113907430-A/G	*CUL4A*	13:113907430	White	COAD	NA	0.911	.
13:113909342-G/A	*CUL4A*	13:113909342	White	LUSC	NA	0.439	.
13:113909342-G/A	*CUL4A*	13:113909342	AA/B	LUSC	NA	0.439	.
13:21549283-T/C	*LATS2*	13:21549283	White	COAD	NA	0.888	.
13:21549283-T/C	*LATS2*	13:21549283	Asian	COAD	NA	0.888	.
13:21549329-G/A	*LATS2*	13:21549329	White	STAD	NA	0.383	.
13:21549329-G/A	*LATS2*	13:21549329	Asian	STAD	NA	0.383	.
13:21549342-G/T	*LATS2*	13:21549342	White	COAD	NA	0.206	.
13:21549425-G/T	*LATS2*	13:21549425	White	SARC	NA	0.168	.
13:21549425-G/T	*LATS2*	13:21549425	AA/B	SARC	NA	0.168	.
13:21555776-T/C	*LATS2*	13:21555776	White	STAD	NA	0.376	.
13:21557416-A/G	*LATS2*	13:21557416	White	STAD	NA	0.726	.
13:21557659-A/T	*LATS2*	13:21557659	AA/B	LGG	NA	0.741	.
13:21557659-A/T	*LATS2*	13:21557659	White	LGG	NA	0.741	.
13:21557821-C/A	*LATS2*	13:21557821	AA/B	LUAD	NA	0.896	.
13:21557821-C/A	*LATS2*	13:21557821	White	LUAD	NA	0.896	.
13:21557936-A/G	*LATS2*	13:21557936	Asian	UCEC	NA	0.203	.
13:24995378-G/T	*PARP4*	13:24995378	White	UCEC	NA	0.558	.
13:25016801-G/T	*PARP4*	13:25016801	Asian	UCEC	NA	0.264	.
13:25029279-A/C	*PARP4*	13:25029279	Asian	UCEC	NA	0.221	.
13:25067745-T/G	*PARP4*	13:25067745	White	STAD	NA	0.374	.
13:25075965-T/C	*PARP4*	13:25075965	Asian	UCEC	rs372717196	0.305	.

13:31037482-G/A	*HMGB1*	13:31037482	White	UCEC	NA	0.413	.
13:32900276-G/T	*BRCA2*	13:32900276	Asian	UCEC	rs377639990	0.168	uncertain_significance
13:32906456-G/T	*BRCA2*	13:32906456	White	COAD	NA	0.168	uncertain_significance,not_provided
13:32906949-C/A	*BRCA2*	13:32906949	White	COAD	NA	0.157	.
13:32907453-T/G	*BRCA2*	13:32907453	White	COAD	rs587780646	0.206	uncertain_significance
13:32910426-G/T	*BRCA2*	13:32910426	White	COAD	NA	0.121	.
13:32912190-C/A	*BRCA2*	13:32912190	White	UCEC	NA	0.492	.
13:32912546-G/T	*BRCA2*	13:32912546	White	COAD	rs80358655	0.152	uncertain_significance,not_provided
13:32914550-G/A	*BRCA2*	13:32914550	White	UCEC	NA	0.221	uncertain_significance,not_provided
13:32930688-G/A	*BRCA2*	13:32930688	White	COAD	rs80358982	0.638	.
13:32930746-G/T	*BRCA2*	13:32930746	Asian	STAD	NA	0.787	.
13:32930746-G/T	*BRCA2*	13:32930746	White	STAD	NA	0.787	.
13:32936758-A/G	*BRCA2*	13:32936758	White	COAD	NA	0.902	.
13:32937356-A/G	*BRCA2*	13:32937356	White	LUAD	NA	0.801	.
13:32937356-A/G	*BRCA2*	13:32937356	AA/B	LUAD	NA	0.801	.

13:32937540-C/A	*BRCA2*	13:32937540	Asian	STAD	NA	0.533	.
13:32937540-C/A	*BRCA2*	13:32937540	White	STAD	NA	0.533	.
13:32945129-C/T	*BRCA2*	13:32945129	White	UCEC	rs80359104	0.843	uncertain_significance
13:32945130-G/A	*BRCA2*	13:32945130	White	COAD	rs80359105	0.856	uncertain_significance,benign,likely_benign
13:32945130-G/A	*BRCA2*	13:32945130	AA/B	COAD	rs80359105	0.856	uncertain_significance,benign,likely_benign
13:32950833-T/A	*BRCA2*	13:32950833	White	KIRC	NA	0.202	.
13:32950833-T/A	*BRCA2*	13:32950833	AA/B	KIRC	NA	0.202	.
13:32968891-G/T	*BRCA2*	13:32968891	White	UCEC	NA	0.691	.
13:32971062-G/C	*BRCA2*	13:32971062	Asian	BRCA	NA	0.552	.
13:32971062-G/C	*BRCA2*	13:32971062	White	BRCA	NA	0.552	.
13:32972645-C/A	*BRCA2*	13:32972645	Asian	UCEC	NA	0.12	.
13:32972674-G/A	*BRCA2*	13:32972674	White	STAD	rs28897761	0.249	uncertain_significance,not_provided,likely_benign
13:32972674-G/A	*BRCA2*	13:32972674	Asian	STAD	rs28897761	0.249	uncertain_significance,not_provided,likely_benign
13:34395290-C/T	*RFC3*	13:34395290	White	CESC	NA	0.744	.

13:34395290-C/T	*RFC3*	13:34395290	AA/B	CESC	NA	0.744	.
13:34404916-G/C	*RFC3*	13:34404916	AA/B	LUAD	NA	0.684	.
13:34404916-G/C	*RFC3*	13:34404916	White	LUAD	NA	0.684	.
13:48615171-T/G	*NUDT15*	13:48615171	White	COAD	NA	0.522	.
13:48615171-T/G	*NUDT15*	13:48615171	Asian	COAD	NA	0.522	.
13:48615235-A/C	*NUDT15*	13:48615235	White	STAD	NA	0.413	.
13:53617325-T/C	*OLFM4*	13:53617325	Asian	STAD	NA	0.904	.
13:53617325-T/C	*OLFM4*	13:53617325	White	STAD	NA	0.904	.
13:53624266-G/T	*OLFM4*	13:53624266	AA/B	LUAD	NA	0.409	.
13:53624266-G/T	*OLFM4*	13:53624266	White	LUAD	NA	0.409	.
13:53624374-A/G	*OLFM4*	13:53624374	Asian	UCEC	NA	0.294	.
13:53624812-G/A	*OLFM4*	13:53624812	White	STAD	rs775118318	0.092	.
13:96212695-G/C	*CLDN10*	13:96212695	White	BRCA	NA	0.846	.
13:96212695-G/C	*CLDN10*	13:96212695	Asian	BRCA	NA	0.846	.
14:104165817-G/A	*XRCC3*	14:104165817	AA/B	BRCA	rs545840795	0.72	.
14:104165817-G/A	*XRCC3*	14:104165817	White	BRCA	rs545840795	0.72	.
14:104173478-C/A	*XRCC3*	14:104173478	AA/B	CESC	NA	0.649	.
14:104173478-C/A	*XRCC3*	14:104173478	White	CESC	NA	0.649	.
14:104174931-G/T	*XRCC3*	14:104174931	Asian	THCA	NA	0.18	.
14:104174931-G/T	*XRCC3*	14:104174931	White	THCA	NA	0.18	.
14:104174939-A/G	*XRCC3*	14:104174939	White	COAD	rs758434542	0.271	.
14:104174939-A/G	*XRCC3*	14:104174939	AA/B	COAD	rs758434542	0.271	.
14:104174984-G/A	*XRCC3*	14:104174984	White	STAD	rs748765569	0.48	.

14:104618692-C/T	*KIF26A*	14:104618692	AA/B	COAD	rs866358165	0.27	.
14:104618692-C/T	*KIF26A*	14:104618692	White	COAD	rs866358165	0.27	.
14:104639387-G/T	*KIF26A*	14:104639387	White	LUAD	NA	0.769	.
14:104639387-G/T	*KIF26A*	14:104639387	AA/B	LUAD	NA	0.769	.
14:104639437-C/T	*KIF26A*	14:104639437	AA/B	CESC	NA	0.789	.
14:104639437-C/T	*KIF26A*	14:104639437	White	CESC	NA	0.789	.
14:104641544-G/A	*KIF26A*	14:104641544	White	COAD	rs1483419517	0.506	.
14:104641544-G/A	*KIF26A*	14:104641544	AA/B	COAD	rs1483419517	0.506	.
14:104641626-A/T	*KIF26A*	14:104641626	White	STAD	NA	0.635	.
14:104641626-A/T	*KIF26A*	14:104641626	Asian	STAD	NA	0.635	.
14:20813570-C/A	*PARP2*	14:20813570	White	UCEC	NA	0.092	.
14:20820412-A/C	*PARP2*	14:20820412	AA/B	OV	NA	0.449	.
14:20820412-A/C	*PARP2*	14:20820412	White	OV	NA	0.449	.
14:20820418-C/T	*PARP2*	14:20820418	White	UCEC	rs778767947	0.593	.
14:20823000-T/C	*PARP2*	14:20823000	White	UCEC	NA	0.443	.
14:20925014-G/A	*APEX1*	14:20925014	White	STAD	rs1471676715	0.822	.
14:20925014-G/A	*APEX1*	14:20925014	White	STAD	rs1471676715	0.474	.
14:20925014-G/A	*APEX1*	14:20925014	Asian	STAD	rs1471676715	0.822	.
14:20925014-G/A	*APEX1*	14:20925014	Asian	STAD	rs1471676715	0.474	.
14:31074996-G/T	*G2E3*	14:31074996	Asian	UCEC	NA	0.208	.
14:31085547-C/T	*G2E3*	14:31085547	White	COAD	NA	0.845	.
14:31085686-A/G	*G2E3*	14:31085686	Asian	UCEC	NA	0.351	.
14:45605397-G/T	*FANCM*	14:45605397	Asian	UCEC	NA	0.293	.

14:45605505-G/A	*FANCM*	14:45605505	White	COAD	NA	0.098	.
14:45605505-G/A	*FANCM*	14:45605505	AA/B	COAD	NA	0.098	.
14:45623909-G/A	*FANCM*	14:45623909	AA/B	COAD	rs530233908	0.192	.
14:45623909-G/A	*FANCM*	14:45623909	White	COAD	rs530233908	0.192	.
14:45633710-C/A	*FANCM*	14:45633710	White	UCEC	NA	0.682	.
14:45642325-C/A	*FANCM*	14:45642325	White	STAD	NA	0.168	.
14:45642325-C/A	*FANCM*	14:45642325	Asian	STAD	NA	0.168	.
14:45657013-T/C	*FANCM*	14:45657013	White	COAD	NA	0.7	.
14:45657013-T/C	*FANCM*	14:45657013	White	COAD	NA	0.269	.
14:45658222-G/T	*FANCM*	14:45658222	AA/B	OV	NA	0.207	.
14:45658222-G/T	*FANCM*	14:45658222	AA/B	OV	NA	0.362	.
14:45658222-G/T	*FANCM*	14:45658222	White	OV	NA	0.207	.
14:45658222-G/T	*FANCM*	14:45658222	White	OV	NA	0.362	.
14:45665712-G/T	*FANCM*	14:45665712	White	COAD	NA	0.39	.
14:45665712-G/T	*FANCM*	14:45665712	White	COAD	NA	0.583	.
14:45667962-G/C	*FANCM*	14:45667962	White	ESCA	NA	0.522	.
14:45667962-G/C	*FANCM*	14:45667962	White	ESCA	NA	0.252	.
14:45667962-G/C	*FANCM*	14:45667962	Asian	ESCA	NA	0.522	.
14:45667962-G/C	*FANCM*	14:45667962	Asian	ESCA	NA	0.252	.
14:50133100-G/A	*POLE2*	14:50133100	White	UCEC	rs779836154	0.263	.
14:50140860-C/T	*POLE2*	14:50140860	White	STAD	rs773570956	0.542	.
14:50140860-C/T	*POLE2*	14:50140860	Asian	STAD	rs773570956	0.542	.
14:61285485-G/T	*MNAT1*	14:61285485	White	UCEC	NA	0.264	.

14:61285492-C/T	*MNAT1*	14:61285492	AA/B	CESC	NA	0.2	.
14:61285492-C/T	*MNAT1*	14:61285492	White	CESC	NA	0.2	.
14:68353826-G/C	*RAD51B*	14:68353826	AA/B	LUAD	NA	0.342	.
14:68353826-G/C	*RAD51B*	14:68353826	White	LUAD	NA	0.342	.
14:75483894-T/C	*MLH3*	14:75483894	White	KIRC	rs1258839619	0.401	.
14:75483894-T/C	*MLH3*	14:75483894	White	KIRC	rs1258839619	0.173	.
14:75483894-T/C	*MLH3*	14:75483894	AA/B	KIRC	rs1258839619	0.401	.
14:75483894-T/C	*MLH3*	14:75483894	AA/B	KIRC	rs1258839619	0.173	.
14:75489558-G/A	*MLH3*	14:75489558	White	UCEC	NA	0.382	.
14:75489558-G/A	*MLH3*	14:75489558	White	UCEC	NA	0.77	.
14:75498786-T/C	*MLH3*	14:75498786	White	LUSC	NA	0.627	.
14:75498786-T/C	*MLH3*	14:75498786	White	LUSC	NA	0.112	.
14:75498786-T/C	*MLH3*	14:75498786	AA/B	LUSC	NA	0.627	.
14:75498786-T/C	*MLH3*	14:75498786	AA/B	LUSC	NA	0.112	.
14:75513607-C/A	*MLH3*	14:75513607	White	STAD	rs762167939	0.533	.
14:75514101-G/T	*MLH3*	14:75514101	Asian	UCEC	rs1216078128	0.235	.
14:75515257-C/A	*MLH3*	14:75515257	White	COAD	rs1345642774	0.659	.
14:75515647-A/G	*MLH3*	14:75515647	White	BRCA	NA	0.461	.
14:75515647-A/G	*MLH3*	14:75515647	Asian	BRCA	NA	0.461	.
14:75515730-C/T	*MLH3*	14:75515730	White	UCEC	rs143631202	0.765	.
14:75516318-C/T	*MLH3*	14:75516318	White	STAD	rs760072474	0.909	.
14:75516318-C/T	*MLH3*	14:75516318	Asian	STAD	rs760072474	0.909	.
14:78140268-A/C	*ALKBH1*	14:78140268	White	UCEC	NA	0.246	.

14:78140396-G/T	*ALKBH1*	14:78140396	Asian	STAD	NA	0.218	.
14:78140396-G/T	*ALKBH1*	14:78140396	White	STAD	NA	0.218	.
14:78142116-C/A	*ALKBH1*	14:78142116	Asian	ESCA	NA	0.48	.
14:78142116-C/A	*ALKBH1*	14:78142116	White	ESCA	NA	0.48	.
14:78174215-C/T	*ALKBH1*	14:78174215	Asian	BRCA	NA	0.182	.
14:78174215-C/T	*ALKBH1*	14:78174215	White	BRCA	NA	0.182	.
14:78174263-A/C	*ALKBH1*	14:78174263	White	UCEC	NA	0.103	.
14:90429903-G/T	*TDP1*	14:90429903	AA/B	BRCA	NA	0.141	.
14:90429903-G/T	*TDP1*	14:90429903	White	BRCA	NA	0.141	.
14:90430003-C/A	*TDP1*	14:90430003	White	THCA	NA	0.34	.
14:90430003-C/A	*TDP1*	14:90430003	Asian	THCA	NA	0.34	.
14:90437554-G/T	*TDP1*	14:90437554	White	KIRC	NA	0.251	.
14:90437554-G/T	*TDP1*	14:90437554	AA/B	KIRC	NA	0.251	.
14:90509479-C/T	*TDP1*	14:90509479	Asian	ESCA	NA	0.282	.
14:90509479-C/T	*TDP1*	14:90509479	White	ESCA	NA	0.282	.
14:94674875-C/T	*PPP4R4*	14:94674875	White	HNSC	rs745732168	0.282	.
14:94674875-C/T	*PPP4R4*	14:94674875	AA/B	HNSC	rs745732168	0.282	.
14:94674896-A/G	*PPP4R4*	14:94674896	AA/B	COAD	NA	0.208	.
14:94674896-A/G	*PPP4R4*	14:94674896	White	COAD	NA	0.208	.
14:94700081-A/C	*PPP4R4*	14:94700081	Asian	UCEC	NA	0.217	.
14:94704023-G/T	*PPP4R4*	14:94704023	AA/B	LUSC	NA	0.735	.
14:94704023-G/T	*PPP4R4*	14:94704023	White	LUSC	NA	0.735	.
14:94712823-G/C	*PPP4R4*	14:94712823	Asian	ESCA	NA	0.544	.

14:94712823-G/C	*PPP4R4*	14:94712823	White	ESCA	NA	0.544	.
14:94718151-G/A	*PPP4R4*	14:94718151	White	UCEC	NA	0.281	.
15:31200384-A/C	*FAN1*	15:31200384	Asian	UCEC	NA	0.287	.
15:31212773-C/A	*FAN1*	15:31212773	White	UCEC	NA	0.601	.
15:31212791-G/C	*FAN1*	15:31212791	White	LUAD	NA	0.878	.
15:31212791-G/C	*FAN1*	15:31212791	AA/B	LUAD	NA	0.878	.
15:31214442-C/T	*FAN1*	15:31214442	White	STAD	NA	0.789	.
15:31214442-C/T	*FAN1*	15:31214442	Asian	STAD	NA	0.789	.
15:31214519-G/T	*FAN1*	15:31214519	AA/B	SARC	NA	0.636	.
15:31214519-G/T	*FAN1*	15:31214519	White	SARC	NA	0.636	.
15:31217427-C/T	*FAN1*	15:31217427	White	COAD	rs56258078	0.31	.
15:31217427-C/T	*FAN1*	15:31217427	Asian	COAD	rs56258078	0.31	.
15:31218089-G/A	*FAN1*	15:31218089	White	STAD	NA	0.602	.
15:31218089-G/A	*FAN1*	15:31218089	Asian	STAD	NA	0.602	.
15:31221547-A/G	*FAN1*	15:31221547	AA/B	LIHC	NA	0.578	.
15:40993345-G/T	*RAD51*	15:40993345	Asian	UCEC	NA	0.29	.
15:41021711-T/G	*RAD51*	15:41021711	White	BRCA	NA	0.747	.
15:41021711-T/G	*RAD51*	15:41021711	Asian	BRCA	NA	0.747	.
15:41275117-C/T	*INO80*	15:41275117	AA/B	COAD	NA	0.453	.
15:41275117-C/T	*INO80*	15:41275117	White	COAD	NA	0.453	.
15:41277616-G/A	*INO80*	15:41277616	White	UCEC	NA	0.16	.
15:41297869-C/A	*INO80*	15:41297869	White	COAD	NA	0.323	.
15:41297869-C/A	*INO80*	15:41297869	AA/B	COAD	NA	0.323	.

15:41348900-G/T	*INO80*	15:41348900	Asian	COAD	NA	0.939	.
15:41348900-G/T	*INO80*	15:41348900	White	COAD	NA	0.939	.
15:41350894-C/T	*INO80*	15:41350894	White	UCEC	NA	0.82	.
15:41364112-A/C	*INO80*	15:41364112	Asian	UCEC	NA	0.78	.
15:41366630-G/T	*INO80*	15:41366630	Asian	UCEC	rs768979105	0.534	.
15:41372056-G/A	*INO80*	15:41372056	White	UCEC	NA	0.331	.
15:41387808-C/A	*INO80*	15:41387808	White	UCEC	NA	0.469	.
15:43701236-T/C	*TP53BP1*	15:43701236	AA/B	BRCA	NA	0.949	.
15:43701236-T/C	*TP53BP1*	15:43701236	AA/B	BRCA	NA	0.656	.
15:43701236-T/C	*TP53BP1*	15:43701236	White	BRCA	NA	0.949	.
15:43701236-T/C	*TP53BP1*	15:43701236	White	BRCA	NA	0.656	.
15:43708431-A/C	*TP53BP1*	15:43708431	AA/B	BRCA	NA	0.528	.
15:43708431-A/C	*TP53BP1*	15:43708431	White	BRCA	NA	0.528	.
15:43712668-A/C	*TP53BP1*	15:43712668	Asian	COAD	NA	0.705	.
15:43712668-A/C	*TP53BP1*	15:43712668	White	COAD	NA	0.705	.
15:43712793-C/T	*TP53BP1*	15:43712793	Asian	STAD	rs770333392	0.213	.
15:43712793-C/T	*TP53BP1*	15:43712793	White	STAD	rs770333392	0.213	.
15:43712857-G/T	*TP53BP1*	15:43712857	White	STAD	NA	0.194	.
15:43712857-G/T	*TP53BP1*	15:43712857	Asian	STAD	NA	0.194	.
15:43738588-C/T	*TP53BP1*	15:43738588	White	UCEC	NA	0.154	.
15:43748803-T/C	*TP53BP1*	15:43748803	White	COAD	NA	0.126	.
15:43749260-C/A	*TP53BP1*	15:43749260	White	COAD	rs751008850	0.14	.
15:43749260-C/A	*TP53BP1*	15:43749260	AA/B	COAD	rs751008850	0.14	.

15:48633554-T/C	*DUT*	15:48633554	White	UCEC	NA	0.446	.
15:75641571-C/T	*NEIL1*	15:75641571	Asian	BRCA	rs774784149	0.015	.
15:75641571-C/T	*NEIL1*	15:75641571	Asian	BRCA	rs774784149	0.109	.
15:75641571-C/T	*NEIL1*	15:75641571	White	BRCA	rs774784149	0.015	.
15:75641571-C/T	*NEIL1*	15:75641571	White	BRCA	rs774784149	0.109	.
15:75641589-G/A	*NEIL1*	15:75641589	Asian	UCEC	rs1428879515	0.299	.
15:75641589-G/A	*NEIL1*	15:75641589	Asian	UCEC	rs1428879515	0.234	.
15:75641646-G/A	*NEIL1*	15:75641646	White	UCEC	rs1372290135	0.636	.
15:75641646-G/A	*NEIL1*	15:75641646	White	UCEC	rs1372290135	0.603	.
15:75644552-C/T	*NEIL1*	15:75644552	AA/B	LUSC	rs1262433075	0.218	.
15:75644552-C/T	*NEIL1*	15:75644552	White	LUSC	rs1262433075	0.218	.
15:79172918-A/C	*MORF4L1*	15:79172918	White	STAD	NA	0.38	.
15:79178521-A/G	*MORF4L1*	15:79178521	White	KIRC	NA	0.415	.
15:79178521-A/G	*MORF4L1*	15:79178521	AA/B	KIRC	NA	0.415	.
15:79178563-C/G	*MORF4L1*	15:79178563	Asian	BRCA	NA	0.151	.
15:79178563-C/G	*MORF4L1*	15:79178563	White	BRCA	NA	0.151	.
15:89169555-C/T	*AEN*	15:89169555	White	UCEC	rs756247681	0.209	.
15:89804904-C/A	*FANCI*	15:89804904	White	COAD	NA	0.327	.
15:89807223-C/A	*FANCI*	15:89807223	White	UCEC	NA	0.487	.
15:89817416-G/T	*FANCI*	15:89817416	Asian	UCEC	NA	0.362	.
15:89828363-G/A	*FANCI*	15:89828363	Asian	UCEC	rs147963936	0.267	.
15:89848408-A/C	*FANCI*	15:89848408	White	UCEC	NA	0.368	.
15:89849327-G/A	*FANCI*	15:89849327	White	STAD	NA	0.628	.

15:89849348-C/T	*FANCI*	15:89849348	AA/B	LUSC	NA	0.752	.
15:89849348-C/T	*FANCI*	15:89849348	White	LUSC	NA	0.752	.
15:89870423-C/T	*POLG*	15:89870423	White	COAD	NA	0.662	.
15:89870423-C/T	*POLG*	15:89870423	AA/B	COAD	NA	0.662	.
15:89870576-G/A	*POLG*	15:89870576	Asian	UCEC	NA	0.943	.
15:89873431-G/A	*POLG*	15:89873431	Asian	BRCA	NA	0.838	.
15:89873431-G/A	*POLG*	15:89873431	White	BRCA	NA	0.838	.
15:89873506-A/G	*POLG*	15:89873506	Asian	UCEC	NA	0.944	.
15:91290665-C/T	*BLM*	15:91290665	AA/B	BRCA	rs148545569	0.182	.
15:91290665-C/T	*BLM*	15:91290665	White	BRCA	rs148545569	0.182	.
15:91293070-G/T	*BLM*	15:91293070	White	UCEC	rs569086568	0.078	.
15:91293192-A/T	*BLM*	15:91293192	White	BRCA	NA	0.055	.
15:91293192-A/T	*BLM*	15:91293192	AA/B	BRCA	NA	0.055	.
15:91295100-G/T	*BLM*	15:91295100	AA/B	COAD	rs146096923	0.221	.
15:91295100-G/T	*BLM*	15:91295100	White	COAD	rs146096923	0.221	.
15:91295128-C/A	*BLM*	15:91295128	White	UCEC	NA	0.401	.
15:91306363-G/T	*BLM*	15:91306363	Asian	STAD	NA	0.681	.
15:91306363-G/T	*BLM*	15:91306363	White	STAD	NA	0.681	.
15:91310169-G/T	*BLM*	15:91310169	White	UCEC	NA	0.067	.
15:91312388-C/T	*BLM*	15:91312388	AA/B	BRCA	NA	0.677	uncertain_significance
15:91312388-C/T	*BLM*	15:91312388	White	BRCA	NA	0.677	uncertain_significance

15:91341532-G/C	*BLM*	15:91341532	White	BRCA	NA	0.645	.
15:91341532-G/C	*BLM*	15:91341532	AA/B	BRCA	NA	0.645	.
15:91347418-A/C	*BLM*	15:91347418	Asian	UCEC	NA	0.148	.
15:91358484-T/G	*BLM*	15:91358484	Asian	STAD	NA	0.333	.
15:91358484-T/G	*BLM*	15:91358484	White	STAD	NA	0.333	.
16:11444642-C/T	*RMI2*	16:11444642	White	STAD	NA	0.207	.
16:135519-C/T	*MPG*	16:135519	White	COAD	NA	0.534	.
16:14020477-C/T	*ERCC4*	16:14020477	Asian	STAD	rs145402255	0.893	.
16:14020477-C/T	*ERCC4*	16:14020477	White	STAD	rs145402255	0.893	.
16:14022058-C/T	*ERCC4*	16:14022058	White	COAD	NA	0.414	.
16:14026041-C/T	*ERCC4*	16:14026041	White	UCEC	rs750883282	0.243	.
16:14029408-C/T	*ERCC4*	16:14029408	White	UCEC	rs368830992	0.298	.
16:14029473-G/A	*ERCC4*	16:14029473	Asian	UCEC	rs55736359	0.354	.
16:14041603-T/C	*ERCC4*	16:14041603	Asian	COAD	NA	0.527	.
16:14041603-T/C	*ERCC4*	16:14041603	White	COAD	NA	0.527	.
16:14041848-C/T	*ERCC4*	16:14041848	White	UCEC	rs121913049	0.51	not_provided,pathogenic
16:1555562-T/A	*TELO2*	16:1555562	AA/B	COAD	NA	0.282	.
16:1555562-T/A	*TELO2*	16:1555562	White	COAD	NA	0.282	.
16:1556293-G/A	*TELO2*	16:1556293	White	STAD	rs145891685	0.365	.
16:1556293-G/A	*TELO2*	16:1556293	Asian	STAD	rs145891685	0.365	.
16:18827649-T/G	*SMG1*	16:18827649	Asian	UCEC	NA	0.392	.
16:18830918-G/T	*SMG1*	16:18830918	White	BRCA	NA	0.447	.

16:18830918-G/T	*SMG1*	16:18830918	Asian	BRCA	NA	0.447	.
16:18840639-T/A	*SMG1*	16:18840639	White	ESCA	NA	0.316	.
16:18840639-T/A	*SMG1*	16:18840639	Asian	ESCA	NA	0.316	.
16:18840712-C/T	*SMG1*	16:18840712	White	UCEC	NA	0.179	.
16:18840904-C/T	*SMG1*	16:18840904	White	COAD	NA	0.318	.
16:18840904-C/T	*SMG1*	16:18840904	AA/B	COAD	NA	0.318	.
16:18844442-C/T	*SMG1*	16:18844442	White	UCEC	NA	0.337	.
16:18845696-G/A	*SMG1*	16:18845696	White	COAD	NA	0.233	.
16:18845696-G/A	*SMG1*	16:18845696	AA/B	COAD	NA	0.233	.
16:18845745-C/A	*SMG1*	16:18845745	White	THCA	NA	0.361	.
16:18845745-C/A	*SMG1*	16:18845745	AA/B	THCA	NA	0.361	.
16:18846365-G/A	*SMG1*	16:18846365	Asian	STAD	NA	0.491	.
16:18846365-G/A	*SMG1*	16:18846365	White	STAD	NA	0.491	.
16:18847333-C/T	*SMG1*	16:18847333	Asian	UCEC	NA	0.462	.
16:18848585-G/T	*SMG1*	16:18848585	AA/B	CESC	NA	0.126	.
16:18848585-G/T	*SMG1*	16:18848585	White	CESC	NA	0.126	.
16:18849875-T/G	*SMG1*	16:18849875	White	COAD	NA	0.863	.
16:18851057-G/T	*SMG1*	16:18851057	White	COAD	NA	0.672	.
16:18851057-G/T	*SMG1*	16:18851057	AA/B	COAD	NA	0.672	.
16:18853011-C/T	*SMG1*	16:18853011	White	CESC	rs749375278	0.706	.
16:18853011-C/T	*SMG1*	16:18853011	AA/B	CESC	rs749375278	0.706	.
16:18856860-T/C	*SMG1*	16:18856860	White	STAD	NA	0.552	.
16:18856864-G/A	*SMG1*	16:18856864	AA/B	BRCA	NA	0.362	.

16:18856864-G/A	*SMG1*	16:18856864	White	BRCA	NA	0.362	.
16:18861600-A/C	*SMG1*	16:18861600	White	OV	NA	0.652	.
16:18861600-A/C	*SMG1*	16:18861600	AA/B	OV	NA	0.652	.
16:18865138-C/T	*SMG1*	16:18865138	White	STAD	NA	0.447	.
16:18865138-C/T	*SMG1*	16:18865138	Asian	STAD	NA	0.447	.
16:18879652-T/C	*SMG1*	16:18879652	White	UCEC	NA	0.454	.
16:18880584-C/G	*SMG1*	16:18880584	AA/B	OV	NA	0.277	.
16:18880584-C/G	*SMG1*	16:18880584	White	OV	NA	0.277	.
16:18882025-T/A	*SMG1*	16:18882025	White	LUAD	NA	0.437	.
16:18882025-T/A	*SMG1*	16:18882025	AA/B	LUAD	NA	0.437	.
16:18882026-C/T	*SMG1*	16:18882026	Asian	UCEC	NA	0.169	.
16:18883506-C/A	*SMG1*	16:18883506	White	UCEC	NA	0.476	.
16:18887715-C/A	*SMG1*	16:18887715	AA/B	COAD	NA	0.357	.
16:18887715-C/A	*SMG1*	16:18887715	White	COAD	NA	0.357	.
16:18896939-T/G	*SMG1*	16:18896939	Asian	UCEC	NA	0.291	.
16:2090176-G/A	*NTHL1*	16:2090176	White	BRCA	NA	0.254	.
16:2090176-G/A	*NTHL1*	16:2090176	Asian	BRCA	NA	0.254	.
16:2090218-C/T	*NTHL1*	16:2090218	White	CESC	NA	0.942	.
16:2090218-C/T	*NTHL1*	16:2090218	AA/B	CESC	NA	0.942	.
16:2094717-G/A	*NTHL1*	16:2094717	White	LUAD	NA	0.537	.
16:2094717-G/A	*NTHL1*	16:2094717	AA/B	LUAD	NA	0.537	.
16:2287477-G/T	*DNASE1L2*	16:2287477	White	SARC	rs1381234602	0.329	.
16:2287477-G/T	*DNASE1L2*	16:2287477	White	SARC	rs1381234602	0.25	.

16:2287477-G/T	*DNASE1L2*	16:2287477	AA/B	SARC	rs1381234602	0.329	.
16:2287477-G/T	*DNASE1L2*	16:2287477	AA/B	SARC	rs1381234602	0.25	.
16:23637631-C/T	*PALB2*	16:23637631	Asian	UCEC	rs45476495	0.273	uncertain_significance
16:23641346-G/A	*PALB2*	16:23641346	White	COAD	rs759024828	0.181	.
16:23641346-G/A	*PALB2*	16:23641346	AA/B	COAD	rs759024828	0.181	.
16:27268789-G/A	*NSMCE1*	16:27268789	White	COAD	rs1444560834	0.15	.
16:27268789-G/A	*NSMCE1*	16:27268789	AA/B	COAD	rs1444560834	0.15	.
16:28970042-A/G	*NFATC2IP*	16:28970042	White	COAD	NA	0.132	.
16:28970042-A/G	*NFATC2IP*	16:28970042	AA/B	COAD	NA	0.132	.
16:28970093-C/T	*NFATC2IP*	16:28970093	White	BRCA	NA	0.145	.
16:28970093-C/T	*NFATC2IP*	16:28970093	Asian	BRCA	NA	0.145	.
16:28975083-C/T	*NFATC2IP*	16:28975083	Asian	STAD	rs370348916	0.056	.
16:28975083-C/T	*NFATC2IP*	16:28975083	White	STAD	rs370348916	0.056	.
16:30094074-G/T	*PPP4C*	16:30094074	AA/B	SARC	NA	0.574	.
16:30094074-G/T	*PPP4C*	16:30094074	White	SARC	NA	0.574	.
16:30094776-A/G	*PPP4C*	16:30094776	AA/B	COAD	NA	0.521	.
16:30094776-A/G	*PPP4C*	16:30094776	White	COAD	NA	0.521	.
16:30096043-G/A	*PPP4C*	16:30096043	Asian	STAD	NA	0.445	.
16:30096043-G/A	*PPP4C*	16:30096043	White	STAD	NA	0.445	.
16:30096088-C/T	*PPP4C*	16:30096088	Asian	UCEC	NA	0.74	.
16:30096288-G/A	*PPP4C*	16:30096288	White	STAD	rs1388779212	0.33	.
16:30096288-G/A	*PPP4C*	16:30096288	Asian	STAD	rs1388779212	0.33	.

16:30096315-T/G	*PPP4C*	16:30096315	White	UCEC	NA	0.481	.
16:30490775-C/T	*ITGAL*	16:30490775	Asian	UCEC	NA	0.351	.
16:30495488-T/A	*ITGAL*	16:30495488	Asian	UCEC	NA	0.72	.
16:30500615-C/A	*ITGAL*	16:30500615	White	UCEC	NA	0.487	.
16:30507854-C/A	*ITGAL*	16:30507854	White	KIRC	NA	0.344	.
16:30507854-C/A	*ITGAL*	16:30507854	AA/B	KIRC	NA	0.344	.
16:30522424-C/A	*ITGAL*	16:30522424	White	STAD	NA	0.398	.
16:30530036-C/T	*ITGAL*	16:30530036	Asian	UCEC	NA	0.176	.
16:3633486-G/A	*SLX4*	16:3633486	White	STAD	rs181782315	0.256	.
16:3633486-G/A	*SLX4*	16:3633486	Asian	STAD	rs181782315	0.256	.
16:3639200-C/T	*SLX4*	16:3639200	White	KIRC	NA	0.121	.
16:3639200-C/T	*SLX4*	16:3639200	AA/B	KIRC	NA	0.121	.
16:3639441-C/T	*SLX4*	16:3639441	White	UCEC	rs376112909	0.175	.
16:3639963-G/A	*SLX4*	16:3639963	AA/B	UCEC	rs142008398	0.055	.
16:3639963-G/A	*SLX4*	16:3639963	White	UCEC	rs142008398	0.055	.
16:3640169-G/A	*SLX4*	16:3640169	Asian	STAD	rs769688259	0.238	.
16:3640169-G/A	*SLX4*	16:3640169	White	STAD	rs769688259	0.238	.
16:3640733-C/A	*SLX4*	16:3640733	White	COAD	NA	0.044	.
16:3640733-C/A	*SLX4*	16:3640733	Asian	COAD	NA	0.044	.
16:3641077-A/C	*SLX4*	16:3641077	Asian	UCEC	NA	0.184	.
16:3641118-C/G	*SLX4*	16:3641118	AA/B	LUSC	NA	0.148	.
16:3641118-C/G	*SLX4*	16:3641118	White	LUSC	NA	0.148	.
16:3647942-G/A	*SLX4*	16:3647942	AA/B	COAD	rs758029323	0.102	.

16:3647942-G/A	*SLX4*	16:3647942	White	COAD	rs758029323	0.102	.
16:3658824-C/T	*SLX4*	16:3658824	White	CESC	NA	0.216	.
16:3658824-C/T	*SLX4*	16:3658824	AA/B	CESC	NA	0.216	.
16:57057706-G/A	*NLRC5*	16:57057706	White	COAD	rs778759493	0.348	.
16:57059388-C/A	*NLRC5*	16:57059388	White	UCEC	NA	0.247	.
16:57059720-G/A	*NLRC5*	16:57059720	Asian	STAD	NA	0.247	.
16:57059720-G/A	*NLRC5*	16:57059720	Asian	STAD	NA	0.033	.
16:57059720-G/A	*NLRC5*	16:57059720	White	STAD	NA	0.247	.
16:57059720-G/A	*NLRC5*	16:57059720	White	STAD	NA	0.033	.
16:57060290-G/T	*NLRC5*	16:57060290	Asian	UCEC	NA	0.621	.
16:57060290-G/T	*NLRC5*	16:57060290	Asian	UCEC	NA	0.438	.
16:57060873-C/A	*NLRC5*	16:57060873	AA/B	KIRC	NA	0.123	.
16:57060873-C/A	*NLRC5*	16:57060873	White	KIRC	NA	0.123	.
16:57070034-G/A	*NLRC5*	16:57070034	White	CESC	NA	0.192	.
16:57070034-G/A	*NLRC5*	16:57070034	White	CESC	NA	0.232	.
16:57070034-G/T	*NLRC5*	16:57070034	Asian	UCEC	NA	0.542	.
16:57070034-G/T	*NLRC5*	16:57070034	Asian	UCEC	NA	0.335	.
16:57081459-T/A	*NLRC5*	16:57081459	AA/B	COAD	NA	0.295	.
16:57081459-T/A	*NLRC5*	16:57081459	White	COAD	NA	0.295	.
16:57088695-C/G	*NLRC5*	16:57088695	AA/B	KIRC	NA	0.072	.
16:57088695-C/G	*NLRC5*	16:57088695	White	KIRC	NA	0.072	.
16:57101739-C/T	*NLRC5*	16:57101739	White	COAD	rs772554645	0.206	.
16:57101739-C/T	*NLRC5*	16:57101739	White	COAD	rs772554645	0.219	.

16:57116382-A/G	*NLRC5*	16:57116382	AA/B	COAD	NA	0.163	.
16:57116382-A/G	*NLRC5*	16:57116382	White	COAD	NA	0.163	.
16:84792352-A/C	*USP10*	16:84792352	White	STAD	NA	0.203	.
16:84806268-C/A	*USP10*	16:84806268	White	COAD	NA	0.524	.
16:84806268-C/A	*USP10*	16:84806268	Asian	COAD	NA	0.524	.
16:84808811-G/A	*USP10*	16:84808811	White	COAD	rs779592460	0.307	.
16:89811418-G/A	*FANCA*	16:89811418	White	STAD	rs780078373	0.272	.
16:89811418-G/A	*FANCA*	16:89811418	Asian	STAD	rs780078373	0.272	.
16:89836275-C/A	*FANCA*	16:89836275	White	UCEC	NA	0.396	.
16:89858352-G/A	*FANCA*	16:89858352	White	UCEC	rs762736991	0.351	.
16:8987895-C/T	*USP7*	16:8987895	White	UCEC	NA	0.224	.
16:8988917-C/T	*USP7*	16:8988917	AA/B	COAD	NA	0.643	.
16:8988917-C/T	*USP7*	16:8988917	White	COAD	NA	0.643	.
16:8999107-C/A	*USP7*	16:8999107	White	LUAD	NA	0.465	.
16:8999107-C/A	*USP7*	16:8999107	AA/B	LUAD	NA	0.465	.
16:9004622-C/T	*USP7*	16:9004622	White	COAD	NA	0.333	.
16:9004622-C/T	*USP7*	16:9004622	Asian	COAD	NA	0.333	.
16:9004637-C/T	*USP7*	16:9004637	White	STAD	NA	0.317	.
16:9004637-C/T	*USP7*	16:9004637	Asian	STAD	NA	0.317	.
16:9012974-C/A	*USP7*	16:9012974	White	LUAD	NA	0.6	.
16:9012974-C/A	*USP7*	16:9012974	AA/B	LUAD	NA	0.6	.
16:9017177-C/T	*USP7*	16:9017177	White	COAD	NA	0.412	.
16:9017177-C/T	*USP7*	16:9017177	Asian	UCEC	NA	0.412	.

17:1791985-C/A	*RPA1*	17:1791985	Asian	UCEC	NA	0.249	.
17:18193997-G/A	*TOP3A*	17:18193997	White	UCEC	NA	0.198	.
17:18202892-G/A	*TOP3A*	17:18202892	Asian	ESCA	NA	0.647	.
17:18202892-G/A	*TOP3A*	17:18202892	White	ESCA	NA	0.647	.
17:18210210-T/C	*TOP3A*	17:18210210	AA/B	COAD	NA	0.196	.
17:18210210-T/C	*TOP3A*	17:18210210	AA/B	COAD	NA	0.29	.
17:18210210-T/C	*TOP3A*	17:18210210	White	COAD	NA	0.196	.
17:18210210-T/C	*TOP3A*	17:18210210	White	COAD	NA	0.29	.
17:29161242-A/C	*ATAD5*	17:29161242	Asian	UCEC	NA	0.174	.
17:29161280-A/G	*ATAD5*	17:29161280	Asian	UCEC	NA	0.194	.
17:29161500-T/C	*ATAD5*	17:29161500	White	COAD	NA	0.054	.
17:29161500-T/C	*ATAD5*	17:29161500	AA/B	COAD	NA	0.054	.
17:29161721-A/G	*ATAD5*	17:29161721	Asian	UCEC	NA	0.144	.
17:29162176-G/T	*ATAD5*	17:29162176	White	UCEC	NA	0.139	.
17:29162562-C/A	*ATAD5*	17:29162562	Asian	UCEC	NA	0.171	.
17:29196541-C/T	*ATAD5*	17:29196541	White	UCEC	NA	0.569	.
17:29196559-C/A	*ATAD5*	17:29196559	Asian	UCEC	NA	0.143	.
17:29206474-T/C	*ATAD5*	17:29206474	White	UCEC	NA	0.353	.
17:29219769-T/G	*ATAD5*	17:29219769	Asian	UCEC	NA	0.363	.
17:33310298-C/T	*LIG3*	17:33310298	White	UCEC	rs151123339	0.28	.
17:33318091-T/G	*LIG3*	17:33318091	Asian	UCEC	NA	0.313	.
17:33318128-G/A	*LIG3*	17:33318128	Asian	ESCA	NA	0.573	.
17:33318128-G/A	*LIG3*	17:33318128	White	ESCA	NA	0.573	.

17:33319550-G/A	*LIG3*	17:33319550	Asian	STAD	NA	0.491	.
17:33319550-G/A	*LIG3*	17:33319550	White	STAD	NA	0.491	.
17:33319550-G/A	*LIG3*	17:33319550	White	UCEC	NA	0.491	.
17:33331364-C/T	*LIG3*	17:33331364	White	STAD	rs756649783	0.402	.
17:33331364-C/T	*LIG3*	17:33331364	Asian	STAD	rs756649783	0.402	.
17:33331400-G/A	*LIG3*	17:33331400	White	UCEC	NA	0.162	.
17:33331509-T/C	*LIG3*	17:33331509	White	LUAD	NA	0.32	.
17:33331509-T/C	*LIG3*	17:33331509	AA/B	LUAD	NA	0.32	.
17:33434083-A/G	*RAD51D*	17:33434083	AA/B	COAD	rs1409370576	0.304	.
17:33434083-A/G	*RAD51D*	17:33434083	White	COAD	rs1409370576	0.304	.
17:33434093-C/T	*RAD51D*	17:33434093	White	UCEC	rs201141245	0.229	.
17:34249635-C/T	*RDM1*	17:34249635	White	STAD	NA	0.294	.
17:37922216-G/A	*IKZF3*	17:37922216	Asian	STAD	NA	0.361	.
17:37922216-G/A	*IKZF3*	17:37922216	White	STAD	NA	0.361	.
17:37922387-G/A	*IKZF3*	17:37922387	Asian	STAD	rs748446589	0.283	.
17:37922387-G/A	*IKZF3*	17:37922387	White	STAD	rs748446589	0.283	.
17:37922668-C/A	*IKZF3*	17:37922668	White	UCEC	NA	0.225	.
17:37922668-C/A	*IKZF3*	17:37922668	White	UCEC	NA	0.272	.
17:37933933-C/T	*IKZF3*	17:37933933	White	UCEC	NA	0.356	.
17:37933933-C/T	*IKZF3*	17:37933933	White	UCEC	NA	0.246	.
17:37985726-A/C	*IKZF3*	17:37985726	White	COAD	NA	0.125	.
17:37985726-A/C	*IKZF3*	17:37985726	Asian	COAD	NA	0.125	.
17:40725136-T/G	*PSMC3IP*	17:40725136	White	THCA	rs374511642	0.369	.

17:40725136-T/G	*PSMC3IP*	17:40725136	AA/B	THCA	rs374511642	0.369	.
17:41234570-T/C	*BRCA1*	17:41234570	Asian	UCEC	NA	0.407	.
17:41234570-T/C	*BRCA1*	17:41234570	Asian	UCEC	NA	0.059	.
17:41243034-C/A	*BRCA1*	17:41243034	White	LUAD	rs80357935	0.275	.
17:41243034-C/A	*BRCA1*	17:41243034	White	LUAD	rs80357935	0.556	.
17:41243034-C/A	*BRCA1*	17:41243034	AA/B	LUAD	rs80357935	0.275	.
17:41243034-C/A	*BRCA1*	17:41243034	AA/B	LUAD	rs80357935	0.556	.
17:41243518-C/G	*BRCA1*	17:41243518	Asian	BRCA	NA	0.026	.
17:41243518-C/G	*BRCA1*	17:41243518	Asian	BRCA	NA	0.541	.
17:41243518-C/G	*BRCA1*	17:41243518	White	BRCA	NA	0.026	.
17:41243518-C/G	*BRCA1*	17:41243518	White	BRCA	NA	0.541	.
17:41245429-C/G	*BRCA1*	17:41245429	White	LUAD	NA	0.437	uncertain_significance
17:41245429-C/G	*BRCA1*	17:41245429	AA/B	LUAD	NA	0.437	uncertain_significance
17:41246648-T/G	*BRCA1*	17:41246648	Asian	UCEC	rs80356861	0.225	uncertain_significance,not_provided
17:41246648-T/G	*BRCA1*	17:41246648	Asian	UCEC	rs80356861	0.745	uncertain_significance,not_provided
17:41247864-C/G	*BRCA1*	17:41247864	White	COAD	NA	0.523	.
17:41247864-C/G	*BRCA1*	17:41247864	Asian	COAD	NA	0.523	.

17:41247865-T/G	*BRCA1*	17:41247865	Asian	ESCA	NA	0.57	not_provided,not_provided,pathogenic
17:41247865-T/G	*BRCA1*	17:41247865	White	ESCA	NA	0.57	not_provided,not_provided,pathogenic
17:41256900-C/G	*BRCA1*	17:41256900	White	BRCA	NA	0.77	uncertain_significance,not_provided
17:41256900-C/G	*BRCA1*	17:41256900	White	BRCA	NA	0.456	uncertain_significance,not_provided
17:41256900-C/G	*BRCA1*	17:41256900	AA/B	BRCA	NA	0.77	uncertain_significance,not_provided
17:41256900-C/G	*BRCA1*	17:41256900	AA/B	BRCA	NA	0.456	uncertain_significance,not_provided
17:41256939-C/A	*BRCA1*	17:41256939	AA/B	BRCA	NA	0.715	.
17:41256939-C/A	*BRCA1*	17:41256939	White	BRCA	NA	0.715	.
17:41256946-A/C	*BRCA1*	17:41256946	White	UCEC	NA	0.639	.
17:48452847-T/G	*EME1*	17:48452847	White	UCEC	NA	0.114	.
17:48453138-C/A	*EME1*	17:48453138	Asian	UCEC	NA	0.078	.

17:48453138-C/A	*EME1*	17:48453138	Asian	UCEC	NA	0.024	.
17:48457845-C/T	*EME1*	17:48457845	White	COAD	NA	0.785	.
17:48457845-C/T	*EME1*	17:48457845	White	COAD	NA	0.485	.
17:48457845-C/T	*EME1*	17:48457845	AA/B	COAD	NA	0.785	.
17:48457845-C/T	*EME1*	17:48457845	AA/B	COAD	NA	0.485	.
17:48458215-G/A	*EME1*	17:48458215	White	UCEC	rs746533275	0.499	.
17:56772483-G/A	*RAD51C*	17:56772483	White	BRCA	NA	0.796	.
17:56772483-G/A	*RAD51C*	17:56772483	Asian	BRCA	NA	0.796	.
17:56774116-T/C	*RAD51C*	17:56774116	Asian	COAD	NA	0.325	.
17:56774116-T/C	*RAD51C*	17:56774116	White	COAD	NA	0.325	.
17:56787293-G/A	*RAD51C*	17:56787293	White	UCEC	rs730881926	0.208	uncertain_significance
17:59821926-C/G	*BRIP1*	17:59821926	AA/B	LUAD	NA	0.796	.
17:59821926-C/G	*BRIP1*	17:59821926	White	LUAD	NA	0.796	.
17:59853777-G/C	*BRIP1*	17:59853777	AA/B	LUAD	NA	0.805	.
17:59853777-G/C	*BRIP1*	17:59853777	White	LUAD	NA	0.805	.
17:59853839-C/A	*BRIP1*	17:59853839	White	KIRC	NA	0.592	.
17:59853839-C/A	*BRIP1*	17:59853839	AA/B	KIRC	NA	0.592	.
17:59857756-A/G	*BRIP1*	17:59857756	Asian	UCEC	NA	0.235	.
17:59861772-G/A	*BRIP1*	17:59861772	White	COAD	NA	0.356	.
17:59878681-A/G	*BRIP1*	17:59878681	White	STAD	NA	0.86	.
17:59878681-A/G	*BRIP1*	17:59878681	Asian	STAD	NA	0.86	.
17:59878801-T/G	*BRIP1*	17:59878801	White	UCEC	NA	0.571	.

17:73627235-G/A	*RECQL5*	17:73627235	White	UCEC	rs375117491	0.703	.
17:73658753-C/T	*RECQL5*	17:73658753	White	COAD	NA	0.614	.
17:73658753-C/T	*RECQL5*	17:73658753	Asian	COAD	NA	0.614	.
17:73658785-C/T	*RECQL5*	17:73658785	AA/B	COAD	rs369553617	0.251	.
17:73658785-C/T	*RECQL5*	17:73658785	White	COAD	rs369553617	0.251	.
17:73658807-G/A	*RECQL5*	17:73658807	White	UCEC	rs972145896	0.286	.
17:7574002-C/G	*TP53*	17:7574002	Asian	BRCA	NA	0.65	.
17:7574002-C/G	*TP53*	17:7574002	White	BRCA	NA	0.65	.
17:7574002-C/G	*TP53*	17:7574002	AA/B	BRCA	NA	0.65	.
17:7574002-C/G	*TP53*	17:7574002	Asian	UCEC	NA	0.65	.
17:7574017-C/G	*TP53*	17:7574017	White	LUAD	NA	0.792	.
17:7574017-C/G	*TP53*	17:7574017	AA/B	LUAD	NA	0.792	.
17:7576889-C/G	*TP53*	17:7576889	White	CESC	NA	0.215	.
17:7576889-C/G	*TP53*	17:7576889	White	CESC	NA	0.839	.
17:7576889-C/G	*TP53*	17:7576889	AA/B	CESC	NA	0.215	.
17:7576889-C/G	*TP53*	17:7576889	AA/B	CESC	NA	0.839	.
17:7577081-T/C	*TP53*	17:7577081	White	OV	NA	0.913	.
17:7577081-T/C	*TP53*	17:7577081	AA/B	OV	NA	0.913	.
17:7577082-C/G	*TP53*	17:7577082	AA/B	LUAD	NA	0.905	.
17:7577082-C/G	*TP53*	17:7577082	White	LUAD	NA	0.905	.
17:7577082-C/T	*TP53*	17:7577082	White	BRCA	NA	0.949	.
17:7577082-C/T	*TP53*	17:7577082	AA/B	BRCA	NA	0.949	.
17:7577082-C/T	*TP53*	17:7577082	AA/B	STAD	NA	0.949	.

17:7577082-C/T	*TP53*	17:7577082	AA/B	SARC	NA	0.949	.
17:7577082-C/T	*TP53*	17:7577082	White	LUAD	NA	0.949	.
17:7577082-C/T	*TP53*	17:7577082	AA/B	LUAD	NA	0.949	.
17:7577082-C/T	*TP53*	17:7577082	White	SARC	NA	0.949	.
17:7577084-T/A	*TP53*	17:7577084	Asian	ESCA	rs121912667	0.936	pathogenic
17:7577084-T/A	*TP53*	17:7577084	White	ESCA	rs121912667	0.936	pathogenic
17:7577085-C/T	*TP53*	17:7577085	Asian	BLCA	NA	0.91	.
17:7577085-C/T	*TP53*	17:7577085	White	BLCA	NA	0.91	.
17:7577094-G/A	*TP53*	17:7577094	White	OV	rs28934574	0.896	likely_benign,pathogenic
17:7577094-G/A	*TP53*	17:7577094	Asian	STAD	rs28934574	0.896	likely_benign,pathogenic
17:7577094-G/A	*TP53*	17:7577094	White	UCEC	rs28934574	0.896	likely_benign,pathogenic
17:7577094-G/A	*TP53*	17:7577094	Asian	BLCA	rs28934574	0.896	likely_benign,pathogenic
17:7577094-G/A	*TP53*	17:7577094	AA/B	HNSC	rs28934574	0.896	likely_benign,pathogenic
17:7577094-G/A	*TP53*	17:7577094	White	STAD	rs28934574	0.896	likely_benign,pathogenic
17:7577094-G/A	*TP53*	17:7577094	AA/B	BRCA	rs28934574	0.896	likely_benign,pathogenic

17:7577094-G/A	*TP53*	17:7577094	AA/B	STAD	rs28934574	0.896	likely_benign,pathogenic
17:7577094-G/A	*TP53*	17:7577094	White	ESCA	rs28934574	0.896	likely_benign,pathogenic
17:7577094-G/A	*TP53*	17:7577094	AA/B	LUSC	rs28934574	0.896	likely_benign,pathogenic
17:7577094-G/A	*TP53*	17:7577094	White	BLCA	rs28934574	0.896	likely_benign,pathogenic
17:7577094-G/A	*TP53*	17:7577094	White	LUSC	rs28934574	0.896	likely_benign,pathogenic
17:7577094-G/A	*TP53*	17:7577094	Asian	ESCA	rs28934574	0.896	likely_benign,pathogenic
17:7577094-G/A	*TP53*	17:7577094	White	HNSC	rs28934574	0.896	likely_benign,pathogenic
17:7577094-G/A	*TP53*	17:7577094	AA/B	OV	rs28934574	0.896	likely_benign,pathogenic
17:7577094-G/A	*TP53*	17:7577094	Asian	HNSC	rs28934574	0.896	likely_benign,pathogenic
17:7577094-G/A	*TP53*	17:7577094	White	BRCA	rs28934574	0.896	likely_benign,pathogenic
17:7577097-C/A	*TP53*	17:7577097	White	LUAD	NA	0.957	.
17:7577097-C/A	*TP53*	17:7577097	AA/B	LUAD	NA	0.957	.

17:7577099-C/T	*TP53*	17:7577099	AA/B	BLCA	NA	0.935	uncertain_significance,pathogenic
17:7577099-C/T	*TP53*	17:7577099	AA/B	CESC	NA	0.935	uncertain_significance,pathogenic
17:7577099-C/T	*TP53*	17:7577099	AA/B	BRCA	NA	0.935	uncertain_significance,pathogenic
17:7577099-C/T	*TP53*	17:7577099	White	CESC	NA	0.935	uncertain_significance,pathogenic
17:7577099-C/T	*TP53*	17:7577099	White	BLCA	NA	0.935	uncertain_significance,pathogenic
17:7577100-T/C	*TP53*	17:7577100	AA/B	HNSC	NA	0.924	.
17:7577100-T/C	*TP53*	17:7577100	White	HNSC	NA	0.924	.
17:7577102-C/T	*TP53*	17:7577102	AA/B	LUSC	NA	0.907	.
17:7577102-C/T	*TP53*	17:7577102	White	LUSC	NA	0.907	.
17:7577106-G/A	*TP53*	17:7577106	White	LUSC	NA	0.963	.
17:7577106-G/A	*TP53*	17:7577106	AA/B	LUSC	NA	0.963	.
17:7577106-G/A	*TP53*	17:7577106	AA/B	HNSC	NA	0.963	.
17:7577106-G/A	*TP53*	17:7577106	White	HNSC	NA	0.963	.
17:7577106-G/C	*TP53*	17:7577106	Asian	ESCA	NA	0.958	.
17:7577106-G/C	*TP53*	17:7577106	White	ESCA	NA	0.958	.
17:7577114-C/A	*TP53*	17:7577114	White	ESCA	NA	0.981	.
17:7577114-C/A	*TP53*	17:7577114	Asian	ESCA	NA	0.981	.
17:7577114-C/A	*TP53*	17:7577114	AA/B	LGG	NA	0.981	.

17:7577114-C/A	*TP53*	17:7577114	White	LGG	NA	0.981	.
17:7577114-C/A	*TP53*	17:7577114	AA/B	HNSC	NA	0.981	.
17:7577114-C/A	*TP53*	17:7577114	White	HNSC	NA	0.981	.
17:7577114-C/T	*TP53*	17:7577114	White	SARC	NA	0.972	.
17:7577114-C/T	*TP53*	17:7577114	AA/B	SARC	NA	0.972	.
17:7577114-C/T	*TP53*	17:7577114	White	BRCA	NA	0.972	.
17:7577114-C/T	*TP53*	17:7577114	AA/B	LIHC	NA	0.972	.
17:7577114-C/T	*TP53*	17:7577114	AA/B	BRCA	NA	0.972	.
17:7577120-C/A	*TP53*	17:7577120	White	OV	rs28934576	0.921	pathogenic
17:7577120-C/A	*TP53*	17:7577120	White	BRCA	rs28934576	0.921	pathogenic
17:7577120-C/A	*TP53*	17:7577120	AA/B	OV	rs28934576	0.921	pathogenic
17:7577120-C/A	*TP53*	17:7577120	AA/B	BRCA	rs28934576	0.921	pathogenic
17:7577120-C/A	*TP53*	17:7577120	White	LUSC	rs28934576	0.921	pathogenic
17:7577120-C/A	*TP53*	17:7577120	Asian	BRCA	rs28934576	0.921	pathogenic
17:7577120-C/A	*TP53*	17:7577120	AA/B	LUSC	rs28934576	0.921	pathogenic
17:7577121-G/A	*TP53*	17:7577121	White	BLCA	rs121913343	0.901	uncertain_significance,pathogenic
17:7577121-G/A	*TP53*	17:7577121	AA/B	LGG	rs121913343	0.901	uncertain_significance,pathogenic
17:7577121-G/A	*TP53*	17:7577121	AA/B	STAD	rs121913343	0.901	uncertain_significance,pathogenic
17:7577121-G/A	*TP53*	17:7577121	White	BRCA	rs121913343	0.901	uncertain_significance,pathogenic

17:7577121-G/A	*TP53*	17:7577121	White	COAD	rs121913343	0.901	uncertain_significance,pathogenic
17:7577121-G/A	*TP53*	17:7577121	White	HNSC	rs121913343	0.901	uncertain_significance,pathogenic
17:7577121-G/A	*TP53*	17:7577121	AA/B	BLCA	rs121913343	0.901	uncertain_significance,pathogenic
17:7577121-G/A	*TP53*	17:7577121	AA/B	COAD	rs121913343	0.901	uncertain_significance,pathogenic
17:7577121-G/A	*TP53*	17:7577121	White	LGG	rs121913343	0.901	uncertain_significance,pathogenic
17:7577121-G/A	*TP53*	17:7577121	AA/B	HNSC	rs121913343	0.901	uncertain_significance,pathogenic
17:7577121-G/A	*TP53*	17:7577121	Asian	STAD	rs121913343	0.901	uncertain_significance,pathogenic
17:7577121-G/A	*TP53*	17:7577121	Asian	HNSC	rs121913343	0.901	uncertain_significance,pathogenic
17:7577121-G/A	*TP53*	17:7577121	Asian	BLCA	rs121913343	0.901	uncertain_significance,pathogenic
17:7577121-G/A	*TP53*	17:7577121	White	STAD	rs121913343	0.901	uncertain_significance,pathogenic
17:7577121-G/C	*TP53*	17:7577121	AA/B	LUAD	NA	0.902	uncertain_significance,pathogenic

17:7577121-G/C	*TP53*	17:7577121	White	LUAD	NA	0.902	uncertain_significance,pathogenic
17:7577121-G/T	*TP53*	17:7577121	White	UCEC	rs121913343	0.906	uncertain_significance,pathogenic
17:7577124-C/A	*TP53*	17:7577124	White	CESC	NA	0.878	uncertain_significance,pathogenic
17:7577124-C/A	*TP53*	17:7577124	AA/B	CESC	NA	0.878	uncertain_significance,pathogenic
17:7577124-C/T	*TP53*	17:7577124	AA/B	OV	rs121912657	0.907	uncertain_significance,pathogenic
17:7577124-C/T	*TP53*	17:7577124	Asian	ESCA	rs121912657	0.907	uncertain_significance,pathogenic
17:7577124-C/T	*TP53*	17:7577124	Asian	STAD	rs121912657	0.907	uncertain_significance,pathogenic
17:7577124-C/T	*TP53*	17:7577124	AA/B	BRCA	rs121912657	0.907	uncertain_significance,pathogenic
17:7577124-C/T	*TP53*	17:7577124	White	BRCA	rs121912657	0.907	uncertain_significance,pathogenic
17:7577124-C/T	*TP53*	17:7577124	White	OV	rs121912657	0.907	uncertain_significance,pathogenic
17:7577124-C/T	*TP53*	17:7577124	AA/B	LUAD	rs121912657	0.907	uncertain_significance,pathogenic

17:7577124-C/T	*TP53*	17:7577124	White	STAD	rs121912657	0.907	uncertain_significance,pathogenic
17:7577124-C/T	*TP53*	17:7577124	White	LUAD	rs121912657	0.907	uncertain_significance,pathogenic
17:7577124-C/T	*TP53*	17:7577124	White	ESCA	rs121912657	0.907	uncertain_significance,pathogenic
17:7577124-C/T	*TP53*	17:7577124	AA/B	STAD	rs121912657	0.907	uncertain_significance,pathogenic
17:7577127-C/T	*TP53*	17:7577127	White	LUSC	NA	0.935	.
17:7577127-C/T	*TP53*	17:7577127	AA/B	LUSC	NA	0.935	.
17:7577130-A/G	*TP53*	17:7577130	Asian	STAD	NA	0.935	.
17:7577130-A/G	*TP53*	17:7577130	White	STAD	NA	0.935	.
17:7577141-C/A	*TP53*	17:7577141	White	LUAD	rs193920774	0.974	uncertain_significance
17:7577141-C/A	*TP53*	17:7577141	AA/B	LUAD	rs193920774	0.974	uncertain_significance
17:7577141-C/T	*TP53*	17:7577141	White	OV	NA	0.973	uncertain_significance
17:7577141-C/T	*TP53*	17:7577141	White	HNSC	NA	0.973	uncertain_significance
17:7577141-C/T	*TP53*	17:7577141	Asian	BRCA	NA	0.973	uncertain_significance

17:7577141-C/T	*TP53*	17:7577141	AA/B	OV	NA	0.973	uncertain_significance
17:7577141-C/T	*TP53*	17:7577141	AA/B	HNSC	NA	0.973	uncertain_significance
17:7577142-C/T	*TP53*	17:7577142	AA/B	LIHC	NA	0.963	.
17:7577144-A/G	*TP53*	17:7577144	White	STAD	NA	0.948	.
17:7577505-T/A	*TP53*	17:7577505	Asian	PAAD	NA	0.742	uncertain_significance
17:7577505-T/A	*TP53*	17:7577505	White	COAD	NA	0.742	uncertain_significance
17:7577505-T/A	*TP53*	17:7577505	White	PAAD	NA	0.742	uncertain_significance
17:7577505-T/A	*TP53*	17:7577505	AA/B	COAD	NA	0.742	uncertain_significance
17:7577509-C/G	*TP53*	17:7577509	White	LUAD	NA	0.936	pathogenic
17:7577509-C/G	*TP53*	17:7577509	AA/B	LUAD	NA	0.936	pathogenic
17:7577511-A/C	*TP53*	17:7577511	AA/B	BRCA	NA	0.922	likely_pathogenic,pathogenic
17:7577511-A/C	*TP53*	17:7577511	White	BRCA	NA	0.922	likely_pathogenic,pathogenic
17:7577532-G/A	*TP53*	17:7577532	White	BRCA	NA	0.962	.
17:7577532-G/A	*TP53*	17:7577532	AA/B	BRCA	NA	0.962	.
17:7577534-C/A	*TP53*	17:7577534	AA/B	LIHC	NA	0.769	pathogenic

17:7577534-C/A	*TP53*	17:7577534	White	LUSC	NA	0.769	pathogenic
17:7577534-C/A	*TP53*	17:7577534	AA/B	LUSC	NA	0.769	pathogenic
17:7577534-C/A	*TP53*	17:7577534	Asian	LIHC	NA	0.769	pathogenic
17:7577534-C/A	*TP53*	17:7577534	White	LIHC	NA	0.769	pathogenic
17:7577534-C/G	*TP53*	17:7577534	AA/B	LUSC	NA	0.769	pathogenic
17:7577534-C/G	*TP53*	17:7577534	White	LUSC	NA	0.769	pathogenic
17:7577538-C/T	*TP53*	17:7577538	Asian	LIHC	rs11540652	0.934	pathogenic
17:7577538-C/T	*TP53*	17:7577538	White	BLCA	rs11540652	0.934	pathogenic
17:7577538-C/T	*TP53*	17:7577538	AA/B	HNSC	rs11540652	0.934	pathogenic
17:7577538-C/T	*TP53*	17:7577538	Asian	ESCA	rs11540652	0.934	pathogenic
17:7577538-C/T	*TP53*	17:7577538	White	ESCA	rs11540652	0.934	pathogenic
17:7577538-C/T	*TP53*	17:7577538	White	STAD	rs11540652	0.934	pathogenic
17:7577538-C/T	*TP53*	17:7577538	Asian	HNSC	rs11540652	0.934	pathogenic
17:7577538-C/T	*TP53*	17:7577538	AA/B	BRCA	rs11540652	0.934	pathogenic
17:7577538-C/T	*TP53*	17:7577538	White	LIHC	rs11540652	0.934	pathogenic
17:7577538-C/T	*TP53*	17:7577538	Asian	BLCA	rs11540652	0.934	pathogenic
17:7577538-C/T	*TP53*	17:7577538	AA/B	STAD	rs11540652	0.934	pathogenic
17:7577538-C/T	*TP53*	17:7577538	White	HNSC	rs11540652	0.934	pathogenic
17:7577538-C/T	*TP53*	17:7577538	White	BRCA	rs11540652	0.934	pathogenic
17:7577538-C/T	*TP53*	17:7577538	Asian	STAD	rs11540652	0.934	pathogenic
17:7577539-G/A	*TP53*	17:7577539	White	COAD	rs121912651	0.927	pathogenic
17:7577539-G/A	*TP53*	17:7577539	Asian	PAAD	rs121912651	0.927	pathogenic
17:7577539-G/A	*TP53*	17:7577539	AA/B	HNSC	rs121912651	0.927	pathogenic

17:7577539-G/A	*TP53*	17:7577539	Asian	ESCA	rs121912651	0.927	pathogenic
17:7577539-G/A	*TP53*	17:7577539	White	LUAD	rs121912651	0.927	pathogenic
17:7577539-G/A	*TP53*	17:7577539	Asian	BRCA	rs121912651	0.927	pathogenic
17:7577539-G/A	*TP53*	17:7577539	White	ESCA	rs121912651	0.927	pathogenic
17:7577539-G/A	*TP53*	17:7577539	White	PAAD	rs121912651	0.927	pathogenic
17:7577539-G/A	*TP53*	17:7577539	White	HNSC	rs121912651	0.927	pathogenic
17:7577539-G/A	*TP53*	17:7577539	Asian	HNSC	rs121912651	0.927	pathogenic
17:7577539-G/A	*TP53*	17:7577539	AA/B	LUAD	rs121912651	0.927	pathogenic
17:7577539-G/A	*TP53*	17:7577539	AA/B	STAD	rs121912651	0.927	pathogenic
17:7577539-G/A	*TP53*	17:7577539	White	UCEC	rs121912651	0.927	pathogenic
17:7577539-G/A	*TP53*	17:7577539	White	STAD	rs121912651	0.927	pathogenic
17:7577539-G/A	*TP53*	17:7577539	AA/B	COAD	rs121912651	0.927	pathogenic
17:7577539-G/A	*TP53*	17:7577539	AA/B	BLCA	rs121912651	0.927	pathogenic
17:7577547-C/A	*TP53*	17:7577547	AA/B	HNSC	rs121912656	0.972	pathogenic
17:7577547-C/A	*TP53*	17:7577547	Asian	ESCA	rs121912656	0.972	pathogenic
17:7577547-C/A	*TP53*	17:7577547	White	HNSC	rs121912656	0.972	pathogenic
17:7577547-C/A	*TP53*	17:7577547	White	ESCA	rs121912656	0.972	pathogenic
17:7577547-C/T	*TP53*	17:7577547	AA/B	OV	rs121912656	0.974	pathogenic
17:7577547-C/T	*TP53*	17:7577547	White	OV	rs121912656	0.974	pathogenic
17:7577548-C/G	*TP53*	17:7577548	AA/B	LUSC	rs28934575	0.961	likely_pathogenic,pathogenic
17:7577548-C/G	*TP53*	17:7577548	White	LUSC	rs28934575	0.961	likely_pathogenic,pathogenic

17:7577548-C/T	*TP53*	17:7577548	Asian	HNSC	rs28934575	0.947	likely_pathogenic,pathogenic
17:7577548-C/T	*TP53*	17:7577548	AA/B	STAD	rs28934575	0.947	likely_pathogenic,pathogenic
17:7577548-C/T	*TP53*	17:7577548	AA/B	HNSC	rs28934575	0.947	likely_pathogenic,pathogenic
17:7577548-C/T	*TP53*	17:7577548	White	HNSC	rs28934575	0.947	likely_pathogenic,pathogenic
17:7577550-C/T	*TP53*	17:7577550	White	LUSC	NA	0.953	.
17:7577550-C/T	*TP53*	17:7577550	AA/B	LUSC	NA	0.953	.
17:7577551-C/A	*TP53*	17:7577551	White	STAD	NA	0.94	.
17:7577551-C/A	*TP53*	17:7577551	Asian	STAD	NA	0.94	.
17:7577551-C/T	*TP53*	17:7577551	White	UCEC	NA	0.936	.
17:7577556-C/A	*TP53*	17:7577556	AA/B	LUSC	NA	0.977	pathogenic
17:7577556-C/A	*TP53*	17:7577556	AA/B	LUAD	NA	0.977	pathogenic
17:7577556-C/A	*TP53*	17:7577556	White	LUAD	NA	0.977	pathogenic
17:7577556-C/A	*TP53*	17:7577556	White	LUSC	NA	0.977	pathogenic
17:7577556-C/T	*TP53*	17:7577556	White	STAD	rs121912655	0.973	pathogenic
17:7577556-C/T	*TP53*	17:7577556	Asian	STAD	rs121912655	0.973	pathogenic
17:7577557-A/C	*TP53*	17:7577557	White	BRCA	NA	0.963	.
17:7577557-A/C	*TP53*	17:7577557	AA/B	BRCA	NA	0.963	.
17:7577559-G/A	*TP53*	17:7577559	White	CESC	rs28934573	0.958	likely_pathogenic,pathogenic

17:7577559-G/A	*TP53*	17:7577559	White	BRCA	rs28934573	0.958	likely_pathogenic,pathogenic
17:7577559-G/A	*TP53*	17:7577559	White	UCEC	rs28934573	0.958	likely_pathogenic,pathogenic
17:7577559-G/A	*TP53*	17:7577559	AA/B	CESC	rs28934573	0.958	likely_pathogenic,pathogenic
17:7577559-G/A	*TP53*	17:7577559	Asian	BRCA	rs28934573	0.958	likely_pathogenic,pathogenic
17:7577559-G/A	*TP53*	17:7577559	Asian	BLCA	rs28934573	0.958	likely_pathogenic,pathogenic
17:7577559-G/A	*TP53*	17:7577559	White	BLCA	rs28934573	0.958	likely_pathogenic,pathogenic
17:7577559-G/A	*TP53*	17:7577559	AA/B	BLCA	rs28934573	0.958	likely_pathogenic,pathogenic
17:7577559-G/C	*TP53*	17:7577559	White	UCEC	rs28934573	0.952	likely_pathogenic,pathogenic
17:7577568-C/A	*TP53*	17:7577568	White	CESC	NA	0.958	likely_pathogenic
17:7577568-C/A	*TP53*	17:7577568	AA/B	HNSC	NA	0.958	likely_pathogenic
17:7577568-C/A	*TP53*	17:7577568	White	LUSC	NA	0.958	likely_pathogenic

17:7577568-C/A	*TP53*	17:7577568	Asian	BRCA	NA	0.958	likely_pathogenic
17:7577568-C/A	*TP53*	17:7577568	AA/B	LUSC	NA	0.958	likely_pathogenic
17:7577570-C/A	*TP53*	17:7577570	White	LUSC	NA	0.923	likely_pathogenic
17:7577570-C/A	*TP53*	17:7577570	AA/B	LUSC	NA	0.923	likely_pathogenic
17:7577570-C/T	*TP53*	17:7577570	White	BRCA	rs587782664	0.923	likely_pathogenic
17:7577570-C/T	*TP53*	17:7577570	AA/B	LUAD	rs587782664	0.923	likely_pathogenic
17:7577570-C/T	*TP53*	17:7577570	White	STAD	rs587782664	0.923	likely_pathogenic
17:7577570-C/T	*TP53*	17:7577570	AA/B	STAD	rs587782664	0.923	likely_pathogenic
17:7577570-C/T	*TP53*	17:7577570	White	LUAD	rs587782664	0.923	likely_pathogenic
17:7577570-C/T	*TP53*	17:7577570	AA/B	BRCA	rs587782664	0.923	likely_pathogenic
17:7577570-C/T	*TP53*	17:7577570	White	LGG	rs587782664	0.923	likely_pathogenic

17:7577570-C/T	*TP53*	17:7577570	AA/B	LGG	rs587782664	0.923	likely_pathogenic
17:7577574-T/C	*TP53*	17:7577574	Asian	HNSC	NA	0.811	uncertain_significance
17:7577575-A/G	*TP53*	17:7577575	White	COAD	NA	0.772	pathogenic
17:7577575-A/G	*TP53*	17:7577575	AA/B	COAD	NA	0.772	pathogenic
17:7577580-T/C	*TP53*	17:7577580	AA/B	LUSC	rs587780073	0.945	pathogenic
17:7577580-T/C	*TP53*	17:7577580	AA/B	OV	rs587780073	0.945	pathogenic
17:7577580-T/C	*TP53*	17:7577580	White	HNSC	rs587780073	0.945	pathogenic
17:7577580-T/C	*TP53*	17:7577580	White	OV	rs587780073	0.945	pathogenic
17:7577580-T/C	*TP53*	17:7577580	White	LUSC	rs587780073	0.945	pathogenic
17:7577586-A/C	*TP53*	17:7577586	Asian	BRCA	NA	0.935	likely_pathogenic
17:7577586-A/C	*TP53*	17:7577586	White	BRCA	NA	0.935	likely_pathogenic
17:7577586-A/G	*TP53*	17:7577586	Asian	UCEC	NA	0.92	likely_pathogenic
17:7577587-T/A	*TP53*	17:7577587	White	LUAD	NA	0.84	.
17:7577587-T/A	*TP53*	17:7577587	AA/B	LUAD	NA	0.84	.
17:7578190-T/C	*TP53*	17:7578190	Asian	LIHC	rs121912666	0.936	pathogenic
17:7578190-T/C	*TP53*	17:7578190	White	UCEC	rs121912666	0.936	pathogenic
17:7578190-T/C	*TP53*	17:7578190	White	LUSC	rs121912666	0.936	pathogenic
17:7578190-T/C	*TP53*	17:7578190	AA/B	LUSC	rs121912666	0.936	pathogenic

17:7578190-T/C	*TP53*	17:7578190	White	BRCA	rs121912666	0.936	pathogenic
17:7578190-T/C	*TP53*	17:7578190	AA/B	BRCA	rs121912666	0.936	pathogenic
17:7578190-T/C	*TP53*	17:7578190	White	LIHC	rs121912666	0.936	pathogenic
17:7578190-T/C	*TP53*	17:7578190	AA/B	STAD	rs121912666	0.936	pathogenic
17:7578190-T/C	*TP53*	17:7578190	Asian	HNSC	rs121912666	0.936	pathogenic
17:7578190-T/C	*TP53*	17:7578190	AA/B	UCEC	rs121912666	0.936	pathogenic
17:7578190-T/G	*TP53*	17:7578190	AA/B	BRCA	NA	0.918	pathogenic
17:7578191-A/C	*TP53*	17:7578191	Asian	ESCA	NA	0.934	.
17:7578191-A/C	*TP53*	17:7578191	White	ESCA	NA	0.934	.
17:7578191-A/G	*TP53*	17:7578191	AA/B	BRCA	rs530941076	0.909	.
17:7578191-A/G	*TP53*	17:7578191	White	BRCA	rs530941076	0.909	.
17:7578205-C/A	*TP53*	17:7578205	AA/B	BRCA	NA	0.913	uncertain_significance
17:7578205-C/A	*TP53*	17:7578205	White	BRCA	NA	0.913	uncertain_significance
17:7578208-T/C	*TP53*	17:7578208	AA/B	LUSC	NA	0.885	.
17:7578208-T/C	*TP53*	17:7578208	White	LUAD	NA	0.885	.
17:7578208-T/C	*TP53*	17:7578208	AA/B	LUAD	NA	0.885	.
17:7578208-T/C	*TP53*	17:7578208	White	LUSC	NA	0.885	.
17:7578211-C/T	*TP53*	17:7578211	White	COAD	rs587778720	0.92	not_provided,pathogenic
17:7578211-C/T	*TP53*	17:7578211	Asian	COAD	rs587778720	0.92	not_provided,pathogenic

17:7578235-T/A	*TP53*	17:7578235	White	PAAD	NA	0.823	.
17:7578235-T/A	*TP53*	17:7578235	Asian	PAAD	NA	0.823	.
17:7578235-T/C	*TP53*	17:7578235	AA/B	LUAD	NA	0.873	.
17:7578235-T/C	*TP53*	17:7578235	White	LUAD	NA	0.873	.
17:7578236-A/T	*TP53*	17:7578236	Asian	BRCA	NA	0.897	.
17:7578236-A/T	*TP53*	17:7578236	White	BRCA	NA	0.897	.
17:7578253-C/A	*TP53*	17:7578253	Asian	BRCA	NA	0.899	.
17:7578265-A/G	*TP53*	17:7578265	Asian	BRCA	rs760043106	0.851	.
17:7578265-A/T	*TP53*	17:7578265	White	STAD	NA	0.84	.
17:7578265-A/T	*TP53*	17:7578265	Asian	STAD	NA	0.84	.
17:7578266-T/A	*TP53*	17:7578266	Asian	ESCA	NA	0.904	.
17:7578266-T/A	*TP53*	17:7578266	White	LUAD	NA	0.904	.
17:7578266-T/A	*TP53*	17:7578266	White	ESCA	NA	0.904	.
17:7578266-T/A	*TP53*	17:7578266	AA/B	LUAD	NA	0.904	.
17:7578268-A/C	*TP53*	17:7578268	AA/B	BRCA	rs1057519998	0.933	.
17:7578268-A/C	*TP53*	17:7578268	White	BRCA	rs1057519998	0.933	.
17:7578268-A/G	*TP53*	17:7578268	Asian	BRCA	NA	0.937	.
17:7578268-A/G	*TP53*	17:7578268	White	BRCA	NA	0.937	.
17:7578271-T/A	*TP53*	17:7578271	Asian	HNSC	NA	0.864	.
17:7578271-T/C	*TP53*	17:7578271	Asian	BRCA	NA	0.884	.
17:7578271-T/G	*TP53*	17:7578271	White	LGG	NA	0.87	.
17:7578271-T/G	*TP53*	17:7578271	AA/B	LGG	NA	0.87	.
17:7578272-G/A	*TP53*	17:7578272	Asian	BRCA	NA	0.887	.

17:7578272-G/A	*TP53*	17:7578272	AA/B	OV	NA	0.887	.
17:7578272-G/A	*TP53*	17:7578272	White	OV	NA	0.887	.
17:7578393-A/T	*TP53*	17:7578393	AA/B	LUAD	rs876660821	0.79	.
17:7578393-A/T	*TP53*	17:7578393	White	LUAD	rs876660821	0.79	.
17:7578394-T/C	*TP53*	17:7578394	White	UCEC	rs1057519991	0.935	.
17:7578394-T/C	*TP53*	17:7578394	White	LGG	rs1057519991	0.935	.
17:7578394-T/C	*TP53*	17:7578394	AA/B	LGG	rs1057519991	0.935	.
17:7578394-T/C	*TP53*	17:7578394	White	ESCA	rs1057519991	0.935	.
17:7578394-T/C	*TP53*	17:7578394	AA/B	STAD	rs1057519991	0.935	.
17:7578394-T/C	*TP53*	17:7578394	AA/B	BRCA	rs1057519991	0.935	.
17:7578394-T/C	*TP53*	17:7578394	Asian	ESCA	rs1057519991	0.935	.
17:7578394-T/C	*TP53*	17:7578394	AA/B	UCEC	rs1057519991	0.935	.
17:7578394-T/C	*TP53*	17:7578394	Asian	HNSC	rs1057519991	0.935	.
17:7578395-G/A	*TP53*	17:7578395	White	BRCA	NA	0.948	pathogenic
17:7578395-G/A	*TP53*	17:7578395	Asian	BRCA	NA	0.948	pathogenic
17:7578403-C/A	*TP53*	17:7578403	White	HNSC	rs786202962	0.951	.
17:7578403-C/A	*TP53*	17:7578403	AA/B	HNSC	rs786202962	0.951	.
17:7578403-C/T	*TP53*	17:7578403	White	ESCA	NA	0.949	.
17:7578403-C/T	*TP53*	17:7578403	Asian	HNSC	NA	0.949	.
17:7578403-C/T	*TP53*	17:7578403	Asian	ESCA	NA	0.949	.
17:7578407-G/A	*TP53*	17:7578407	White	UCEC	rs138729528	0.833	.
17:7578407-G/A	*TP53*	17:7578407	AA/B	COAD	rs138729528	0.833	.
17:7578410-T/A	*TP53*	17:7578410	Asian	ESCA	NA	0.634	.

17:7578410-T/A	*TP53*	17:7578410	White	ESCA	NA	0.634	.
17:7578413-C/A	*TP53*	17:7578413	White	LUAD	NA	0.88	.
17:7578413-C/A	*TP53*	17:7578413	White	COAD	NA	0.88	.
17:7578413-C/A	*TP53*	17:7578413	AA/B	LUAD	NA	0.88	.
17:7578413-C/A	*TP53*	17:7578413	AA/B	LGG	NA	0.88	.
17:7578413-C/A	*TP53*	17:7578413	AA/B	COAD	NA	0.88	.
17:7578413-C/A	*TP53*	17:7578413	White	LGG	NA	0.88	.
17:7578413-C/T	*TP53*	17:7578413	AA/B	HNSC	NA	0.892	.
17:7578413-C/T	*TP53*	17:7578413	White	HNSC	NA	0.892	.
17:7578413-C/T	*TP53*	17:7578413	White	STAD	NA	0.892	.
17:7578427-T/C	*TP53*	17:7578427	Asian	ESCA	NA	0.562	.
17:7578427-T/C	*TP53*	17:7578427	White	ESCA	NA	0.562	.
17:7578427-T/G	*TP53*	17:7578427	Asian	BRCA	NA	0.526	.
17:7578427-T/G	*TP53*	17:7578427	White	BRCA	NA	0.526	.
17:7578442-T/C	*TP53*	17:7578442	White	OV	NA	0.872	pathogenic
17:7578442-T/C	*TP53*	17:7578442	White	LUAD	NA	0.872	pathogenic
17:7578442-T/C	*TP53*	17:7578442	AA/B	HNSC	NA	0.872	pathogenic
17:7578442-T/C	*TP53*	17:7578442	AA/B	OV	NA	0.872	pathogenic
17:7578442-T/C	*TP53*	17:7578442	White	LUSC	NA	0.872	pathogenic
17:7578442-T/C	*TP53*	17:7578442	AA/B	BRCA	NA	0.872	pathogenic
17:7578442-T/C	*TP53*	17:7578442	AA/B	LUAD	NA	0.872	pathogenic
17:7578442-T/C	*TP53*	17:7578442	AA/B	LUSC	NA	0.872	pathogenic
17:7578442-T/C	*TP53*	17:7578442	White	HNSC	NA	0.872	pathogenic

17:7578446-T/A	*TP53*	17:7578446	Asian	STAD	NA	0.676	.
17:7578446-T/A	*TP53*	17:7578446	White	STAD	NA	0.676	.
17:7578449-C/A	*TP53*	17:7578449	AA/B	LIHC	NA	0.752	uncertain_significance,likely_pathogenic
17:7578449-C/T	*TP53*	17:7578449	White	COAD	NA	0.79	uncertain_significance,likely_pathogenic
17:7578449-C/T	*TP53*	17:7578449	AA/B	COAD	NA	0.79	uncertain_significance,likely_pathogenic
17:7578454-G/A	*TP53*	17:7578454	White	LUSC	NA	0.774	.
17:7578454-G/A	*TP53*	17:7578454	AA/B	CESC	NA	0.774	.
17:7578454-G/A	*TP53*	17:7578454	Asian	BLCA	NA	0.774	.
17:7578454-G/A	*TP53*	17:7578454	AA/B	LUSC	NA	0.774	.
17:7578454-G/A	*TP53*	17:7578454	Asian	UCEC	NA	0.774	.
17:7578454-G/A	*TP53*	17:7578454	White	CESC	NA	0.774	.
17:7578455-C/G	*TP53*	17:7578455	White	LUAD	NA	0.815	uncertain_significance
17:7578455-C/G	*TP53*	17:7578455	AA/B	LUAD	NA	0.815	uncertain_significance
17:7578457-C/A	*TP53*	17:7578457	Asian	ESCA	rs587782144	0.891	pathogenic
17:7578457-C/A	*TP53*	17:7578457	White	ESCA	rs587782144	0.891	pathogenic

17:7578460-A/T	*TP53*	17:7578460	White	BRCA	NA	0.863	.
17:7578460-A/T	*TP53*	17:7578460	AA/B	BRCA	NA	0.863	.
17:7578461-C/A	*TP53*	17:7578461	Asian	ESCA	NA	0.708	uncertain_significance,pathogenic
17:7578461-C/A	*TP53*	17:7578461	White	ESCA	NA	0.708	uncertain_significance,pathogenic
17:7578461-C/A	*TP53*	17:7578461	Asian	LIHC	NA	0.708	uncertain_significance,pathogenic
17:7578461-C/A	*TP53*	17:7578461	Asian	HNSC	NA	0.708	uncertain_significance,pathogenic
17:7578461-C/A	*TP53*	17:7578461	White	LIHC	NA	0.708	uncertain_significance,pathogenic
17:7578461-C/A	*TP53*	17:7578461	AA/B	HNSC	NA	0.708	uncertain_significance,pathogenic
17:7578461-C/A	*TP53*	17:7578461	White	HNSC	NA	0.708	uncertain_significance,pathogenic
17:7578467-T/G	*TP53*	17:7578467	White	LUSC	NA	0.581	.
17:7578467-T/G	*TP53*	17:7578467	AA/B	LUSC	NA	0.581	.
17:7578469-C/A	*TP53*	17:7578469	White	LUSC	NA	0.754	.
17:7578469-C/A	*TP53*	17:7578469	AA/B	LUSC	NA	0.754	.
17:7578475-G/T	*TP53*	17:7578475	White	LUSC	NA	0.843	pathogenic
17:7578475-G/T	*TP53*	17:7578475	AA/B	LUSC	NA	0.843	pathogenic
17:7578478-G/C	*TP53*	17:7578478	White	PAAD	NA	0.912	.

17:7578478-G/C	*TP53*	17:7578478	Asian	PAAD	NA	0.912	.
17:7578478-G/T	*TP53*	17:7578478	AA/B	LIHC	NA	0.87	.
17:7578479-G/A	*TP53*	17:7578479	White	UCEC	NA	0.898	likely_pathogenic,pathogenic
17:7578479-G/A	*TP53*	17:7578479	White	HNSC	NA	0.898	likely_pathogenic,pathogenic
17:7578479-G/T	*TP53*	17:7578479	AA/B	STAD	NA	0.911	likely_pathogenic,pathogenic
17:7578503-C/T	*TP53*	17:7578503	White	HNSC	NA	0.659	uncertain_significance
17:7578503-C/T	*TP53*	17:7578503	AA/B	HNSC	NA	0.659	uncertain_significance
17:7578508-C/T	*TP53*	17:7578508	White	UCEC	NA	0.897	pathogenic
17:7578509-A/G	*TP53*	17:7578509	Asian	BRCA	NA	0.861	.
17:7578509-A/G	*TP53*	17:7578509	White	BRCA	NA	0.861	.
17:7578523-T/G	*TP53*	17:7578523	White	BRCA	NA	0.964	.
17:7578523-T/G	*TP53*	17:7578523	AA/B	BRCA	NA	0.964	.
17:7578526-C/A	*TP53*	17:7578526	Asian	ESCA	NA	0.96	uncertain_significance
17:7578526-C/A	*TP53*	17:7578526	AA/B	BRCA	NA	0.96	uncertain_significance
17:7578526-C/A	*TP53*	17:7578526	White	ESCA	NA	0.96	uncertain_significance

17:7578526-C/A	*TP53*	17:7578526	White	BRCA	NA	0.96	uncertain_significance
17:7578526-C/A	*TP53*	17:7578526	Asian	STAD	NA	0.96	uncertain_significance
17:7578526-C/A	*TP53*	17:7578526	White	STAD	NA	0.96	uncertain_significance
17:7578527-A/G	*TP53*	17:7578527	White	ESCA	NA	0.964	.
17:7578527-A/G	*TP53*	17:7578527	AA/B	BRCA	NA	0.964	.
17:7578527-A/G	*TP53*	17:7578527	Asian	ESCA	NA	0.964	.
17:7578527-A/G	*TP53*	17:7578527	White	BRCA	NA	0.964	.
17:7578528-A/T	*TP53*	17:7578528	Asian	ESCA	NA	0.872	.
17:7578528-A/T	*TP53*	17:7578528	White	ESCA	NA	0.872	.
17:7578534-C/G	*TP53*	17:7578534	White	BLCA	NA	0.895	.
17:7578534-C/G	*TP53*	17:7578534	Asian	BLCA	NA	0.895	.
17:7578535-T/C	*TP53*	17:7578535	AA/B	SARC	rs1057519996	0.908	.
17:7578535-T/C	*TP53*	17:7578535	White	SARC	rs1057519996	0.908	.
17:7578536-T/C	*TP53*	17:7578536	AA/B	BRCA	rs747342068	0.968	.
17:7578538-T/A	*TP53*	17:7578538	Asian	PAAD	NA	0.939	.
17:7578538-T/A	*TP53*	17:7578538	AA/B	LUSC	NA	0.939	.
17:7578538-T/A	*TP53*	17:7578538	White	PAAD	NA	0.939	.
17:7578538-T/A	*TP53*	17:7578538	White	LUSC	NA	0.939	.
17:7578542-G/C	*TP53*	17:7578542	Asian	STAD	NA	0.887	.
17:7578542-G/C	*TP53*	17:7578542	White	STAD	NA	0.887	.

17:7578550-G/A	*TP53*	17:7578550	Asian	COAD	NA	0.925	likely_pathogenic
17:7578550-G/A	*TP53*	17:7578550	White	COAD	NA	0.925	likely_pathogenic
17:7578550-G/A	*TP53*	17:7578550	AA/B	LUAD	NA	0.925	likely_pathogenic
17:7578550-G/A	*TP53*	17:7578550	White	LUAD	NA	0.925	likely_pathogenic
17:7578550-G/C	*TP53*	17:7578550	White	LUAD	NA	0.916	likely_pathogenic
17:7578550-G/C	*TP53*	17:7578550	AA/B	LUAD	NA	0.916	likely_pathogenic
17:7578550-G/T	*TP53*	17:7578550	AA/B	BRCA	NA	0.918	likely_pathogenic
17:7578550-G/T	*TP53*	17:7578550	White	BRCA	NA	0.918	likely_pathogenic
17:7578551-A/T	*TP53*	17:7578551	White	COAD	NA	0.924	.
17:7578551-A/T	*TP53*	17:7578551	AA/B	COAD	NA	0.924	.
17:7579313-G/A	*TP53*	17:7579313	Asian	STAD	NA	0.925	.
17:7579313-G/A	*TP53*	17:7579313	White	STAD	NA	0.925	.
17:7579329-T/C	*TP53*	17:7579329	AA/B	LUAD	NA	0.925	pathogenic
17:7579329-T/C	*TP53*	17:7579329	White	LUAD	NA	0.925	pathogenic
17:7579349-A/C	*TP53*	17:7579349	AA/B	BRCA	NA	0.952	.

17:7579355-A/G	*TP53*	17:7579355	AA/B	BRCA	NA	0.864	.
17:7579355-A/T	*TP53*	17:7579355	White	STAD	NA	0.869	.
17:7579355-A/T	*TP53*	17:7579355	Asian	STAD	NA	0.869	.
17:7579358-C/A	*TP53*	17:7579358	Asian	HNSC	rs11540654	0.632	.
17:7579358-C/A	*TP53*	17:7579358	White	ESCA	rs11540654	0.632	.
17:7579358-C/A	*TP53*	17:7579358	Asian	ESCA	rs11540654	0.632	.
17:7579374-C/A	*TP53*	17:7579374	AA/B	LUAD	NA	0.942	.
17:7579374-C/A	*TP53*	17:7579374	White	LUAD	NA	0.942	.
17:78397370-G/C	*ENDOV*	17:78397370	White	BRCA	NA	0.844	.
17:78397370-G/C	*ENDOV*	17:78397370	White	BRCA	NA	0.857	.
17:78397370-G/C	*ENDOV*	17:78397370	Asian	BRCA	NA	0.844	.
17:78397370-G/C	*ENDOV*	17:78397370	Asian	BRCA	NA	0.857	.
17:8045732-C/T	*PER1*	17:8045732	White	COAD	rs371070132	0.266	.
17:8045732-C/T	*PER1*	17:8045732	AA/B	COAD	rs371070132	0.266	.
17:8046045-C/A	*PER1*	17:8046045	White	UCEC	NA	0.315	.
17:8046116-G/A	*PER1*	17:8046116	White	BRCA	NA	0.158	.
17:8046116-G/A	*PER1*	17:8046116	Asian	BRCA	NA	0.158	.
17:8050807-A/G	*PER1*	17:8050807	White	BRCA	NA	0.52	.
17:8050807-A/G	*PER1*	17:8050807	AA/B	BRCA	NA	0.52	.
17:8051032-G/A	*PER1*	17:8051032	Asian	UCEC	rs771047267	0.428	.
17:8053432-G/A	*PER1*	17:8053432	Asian	BRCA	NA	0.264	.
17:8053432-G/A	*PER1*	17:8053432	White	BRCA	NA	0.264	.
18:13734563-G/T	*RNMT*	18:13734563	White	STAD	NA	0.876	.

18:13737129-G/A	*RNMT*	18:13737129	AA/B	KIRC	NA	0.687	.
18:13737129-G/A	*RNMT*	18:13737129	White	KIRC	NA	0.687	.
18:13741578-C/T	*RNMT*	18:13741578	Asian	UCEC	NA	0.957	.
18:19110356-C/T	*ESCO1*	18:19110356	White	UCEC	NA	0.589	.
18:19110434-C/T	*ESCO1*	18:19110434	White	STAD	rs1319414766	0.89	.
18:19110434-C/T	*ESCO1*	18:19110434	Asian	STAD	rs1319414766	0.89	.
18:19112497-C/T	*ESCO1*	18:19112497	White	UCEC	18:19112497_C/T	0.856	.
18:19116140-C/T	*ESCO1*	18:19116140	White	UCEC	NA	0.507	.
18:19119885-T/G	*ESCO1*	18:19119885	White	COAD	NA	0.328	.
18:19153336-T/G	*ESCO1*	18:19153336	White	COAD	NA	0.092	.
18:19153339-A/T	*ESCO1*	18:19153339	White	STAD	NA	0.166	.
18:19153339-A/T	*ESCO1*	18:19153339	Asian	STAD	NA	0.166	.
18:19153612-T/G	*ESCO1*	18:19153612	Asian	UCEC	NA	0.176	.
18:19153644-C/A	*ESCO1*	18:19153644	White	UCEC	NA	0.14	.
18:19154028-C/A	*ESCO1*	18:19154028	Asian	UCEC	NA	0.016	.
18:20548792-G/A	*RBBP8*	18:20548792	White	COAD	rs369500650	0.579	.
18:20572926-C/T	*RBBP8*	18:20572926	White	SARC	NA	0.261	.
18:20572926-C/T	*RBBP8*	18:20572926	AA/B	SARC	NA	0.261	.
18:20573261-C/G	*RBBP8*	18:20573261	White	LUAD	rs561139689	0.223	.
18:20573261-C/G	*RBBP8*	18:20573261	AA/B	LUAD	rs561139689	0.223	.
18:20576348-G/C	*RBBP8*	18:20576348	White	CESC	NA	0.175	.
18:20577626-A/G	*RBBP8*	18:20577626	White	COAD	NA	0.226	.

18:20577626-A/G	*RBBP8*	18:20577626	AA/B	COAD	NA	0.226	.
18:20602198-T/C	*RBBP8*	18:20602198	AA/B	CESC	NA	0.33	.
18:20602198-T/C	*RBBP8*	18:20602198	White	CESC	NA	0.33	.
18:20606106-G/A	*RBBP8*	18:20606106	White	UCEC	rs745335628	0.36	.
18:20606106-G/A	*RBBP8*	18:20606106	White	UCEC	rs745335628	0.079	.
18:51809249-T/C	*POLI*	18:51809249	White	COAD	NA	0.344	.
18:51809249-T/C	*POLI*	18:51809249	Asian	COAD	NA	0.344	.
18:51813698-G/A	*POLI*	18:51813698	White	UCEC	rs950431386	0.442	.
18:670835-A/G	*TYMS*	18:670835	Asian	COAD	NA	0.841	.
18:670835-A/G	*TYMS*	18:670835	White	COAD	NA	0.841	.
18:9550118-C/G	*PPP4R1*	18:9550118	Asian	ESCA	NA	0.155	.
18:9550118-C/G	*PPP4R1*	18:9550118	White	ESCA	NA	0.155	.
18:9577173-A/G	*PPP4R1*	18:9577173	White	COAD	NA	0.668	.
19:13063545-G/A	*RAD23A*	19:13063545	White	UCEC	NA	0.276	.
19:17384941-G/A	*BABAM1*	19:17384941	White	COAD	NA	0.217	.
19:17384941-G/A	*BABAM1*	19:17384941	AA/B	COAD	NA	0.217	.
19:17384974-C/T	*BABAM1*	19:17384974	White	UCEC	rs765161255	0.279	.
19:41510273-G/A	*CYP2B6*	19:41510273	White	STAD	rs774036855	0.686	.
19:41515957-C/T	*CYP2B6*	19:41515957	White	ESCA	rs1315971029	0.246	.
19:41515957-C/T	*CYP2B6*	19:41515957	Asian	ESCA	rs1315971029	0.246	.
19:41518311-G/T	*CYP2B6*	19:41518311	Asian	UCEC	NA	0.962	.
19:44047624-G/A	*XRCC1*	19:44047624	Asian	UCEC	rs199876205	0.673	.
19:44055742-G/A	*XRCC1*	19:44055742	Asian	BRCA	rs746331654	0.215	.

19:44055742-G/A	*XRCC1*	19:44055742	White	BRCA	rs746331654	0.215	.
19:44056203-G/A	*XRCC1*	19:44056203	White	UCEC	rs754041352	0.644	.
19:44057634-G/T	*XRCC1*	19:44057634	White	STAD	NA	0.103	.
19:44057634-G/T	*XRCC1*	19:44057634	Asian	STAD	NA	0.103	.
19:44079569-G/T	*XRCC1*	19:44079569	White	SARC	NA	0.377	.
19:44079569-G/T	*XRCC1*	19:44079569	AA/B	SARC	NA	0.377	.
19:4430593-C/A	*CHAF1A*	19:4430593	Asian	UCEC	NA	0.385	.
19:4432060-G/A	*CHAF1A*	19:4432060	Asian	UCEC	NA	0.566	.
19:4432190-G/T	*CHAF1A*	19:4432190	White	COAD	NA	0.08	.
19:4432190-G/T	*CHAF1A*	19:4432190	AA/B	COAD	NA	0.08	.
19:4433108-G/A	*CHAF1A*	19:4433108	White	COAD	rs761903668	0.389	.
19:4433108-G/A	*CHAF1A*	19:4433108	AA/B	COAD	rs761903668	0.389	.
19:4433137-G/T	*CHAF1A*	19:4433137	AA/B	LUAD	NA	0.25	.
19:4433137-G/T	*CHAF1A*	19:4433137	White	LUAD	NA	0.25	.
19:45855493-G/A	*ERCC2*	19:45855493	White	UCEC	rs121913026	0.795	pathogenic
19:45855888-C/T	*ERCC2*	19:45855888	White	UCEC	rs747476170	0.775	.
19:45855889-G/A	*ERCC2*	19:45855889	White	COAD	rs769085031	0.787	.
19:45856015-G/A	*ERCC2*	19:45856015	White	STAD	rs144511865	0.808	.
19:45856015-G/A	*ERCC2*	19:45856015	Asian	STAD	rs144511865	0.808	.
19:45858934-C/T	*ERCC2*	19:45858934	White	COAD	rs772572683	0.907	.
19:45858934-C/T	*ERCC2*	19:45858934	AA/B	COAD	rs772572683	0.907	.
19:45860787-G/A	*ERCC2*	19:45860787	White	ESCA	rs760834335	0.861	.
19:45860787-G/A	*ERCC2*	19:45860787	Asian	ESCA	rs760834335	0.861	.

19:45864875-G/T	*ERCC2*	19:45864875	Asian	STAD	NA	0.544	.
19:45864875-G/T	*ERCC2*	19:45864875	White	STAD	NA	0.544	.
19:45868140-C/A	*ERCC2*	19:45868140	White	KIRP	NA	0.819	.
19:45868140-C/A	*ERCC2*	19:45868140	AA/B	KIRP	NA	0.819	.
19:45872219-T/C	*ERCC2*	19:45872219	White	BLCA	NA	0.82	.
19:45872374-G/A	*ERCC2*	19:45872374	White	UCEC	NA	0.816	.
19:45916943-G/T	*ERCC1*	19:45916943	White	STAD	NA	0.387	.
19:45916943-G/T	*ERCC1*	19:45916943	Asian	STAD	NA	0.387	.
19:45916982-C/T	*ERCC1*	19:45916982	White	UCEC	rs3212977	0.32	.
19:45918121-G/A	*ERCC1*	19:45918121	White	COAD	rs772941786	0.392	.
19:45918121-G/A	*ERCC1*	19:45918121	Asian	COAD	rs772941786	0.392	.
19:45924443-G/A	*ERCC1*	19:45924443	White	UCEC	NA	0.27	.
19:45974486-G/A	*FOSB*	19:45974486	White	COAD	NA	0.693	.
19:45974486-G/A	*FOSB*	19:45974486	AA/B	COAD	NA	0.693	.
19:45974502-A/C	*FOSB*	19:45974502	White	CESC	NA	0.451	.
19:45976235-G/A	*FOSB*	19:45976235	AA/B	BRCA	NA	0.172	.
19:45976235-G/A	*FOSB*	19:45976235	White	BRCA	NA	0.172	.
19:45976262-G/T	*FOSB*	19:45976262	AA/B	LIHC	NA	0.219	.
19:48619186-G/A	*LIG1*	19:48619186	White	CESC	NA	0.794	.
19:48619186-G/A	*LIG1*	19:48619186	AA/B	CESC	NA	0.794	.
19:48636268-C/T	*LIG1*	19:48636268	White	COAD	NA	0.882	pathogenic
19:48636294-C/T	*LIG1*	19:48636294	White	UCEC	rs746737248	0.623	.
19:48660276-C/T	*LIG1*	19:48660276	White	UCEC	rs368094694	0.112	.

19:48660279-C/T	*LIG1*	19:48660279	White	STAD	rs377155700	0.23	.
19:48660279-C/T	*LIG1*	19:48660279	Asian	STAD	rs377155700	0.23	.
19:50905546-G/A	*POLD1*	19:50905546	White	COAD	rs144979965	0.201	.
19:50905546-G/A	*POLD1*	19:50905546	AA/B	COAD	rs144979965	0.201	.
19:50905630-G/A	*POLD1*	19:50905630	AA/B	COAD	rs1366413924	0.489	.
19:50905630-G/A	*POLD1*	19:50905630	White	COAD	rs1366413924	0.489	.
19:50905974-G/A	*POLD1*	19:50905974	White	UCEC	NA	0.587	.
19:50906796-G/A	*POLD1*	19:50906796	AA/B	COAD	NA	0.673	.
19:50906796-G/A	*POLD1*	19:50906796	White	COAD	NA	0.673	.
19:50909556-C/T	*POLD1*	19:50909556	White	COAD	NA	0.451	.
19:50909556-C/T	*POLD1*	19:50909556	AA/B	COAD	NA	0.451	.
19:50910318-C/T	*POLD1*	19:50910318	White	STAD	rs201804732	0.267	.
19:50910318-C/T	*POLD1*	19:50910318	Asian	STAD	rs201804732	0.267	.
19:50910642-C/T	*POLD1*	19:50910642	White	UCEC	rs547831370	0.162	.
19:50912856-G/T	*POLD1*	19:50912856	AA/B	SARC	NA	0.512	.
19:50912856-G/T	*POLD1*	19:50912856	White	SARC	NA	0.512	.
19:50917018-A/G	*POLD1*	19:50917018	White	UCEC	NA	0.97	.
19:50917041-G/A	*POLD1*	19:50917041	AA/B	COAD	rs759190487	0.313	.
19:50917041-G/A	*POLD1*	19:50917041	White	COAD	rs759190487	0.313	.
19:55493959-G/T	*NLRP2*	19:55493959	Asian	STAD	NA	0.284	.
19:55493959-G/T	*NLRP2*	19:55493959	White	STAD	NA	0.284	.
19:55494170-C/A	*NLRP2*	19:55494170	White	LUAD	NA	0.49	.
19:55494170-C/A	*NLRP2*	19:55494170	AA/B	LUAD	NA	0.49	.

19:55494276-G/A	*NLRP2*	19:55494276	Asian	STAD	rs763533364	0.305	.
19:55494276-G/A	*NLRP2*	19:55494276	White	STAD	rs763533364	0.305	.
19:55494535-G/A	*NLRP2*	19:55494535	Asian	COAD	rs767901107	0.228	.
19:55494535-G/A	*NLRP2*	19:55494535	White	COAD	rs767901107	0.228	.
19:55494940-T/C	*NLRP2*	19:55494940	White	COAD	NA	0.145	.
19:55495061-T/A	*NLRP2*	19:55495061	White	LUAD	NA	0.222	.
19:55495061-T/A	*NLRP2*	19:55495061	AA/B	LUAD	NA	0.222	.
19:55496486-T/G	*NLRP2*	19:55496486	Asian	UCEC	NA	0.518	.
19:55497679-C/A	*NLRP2*	19:55497679	White	UCEC	NA	0.359	not_provided
19:55505722-C/A	*NLRP2*	19:55505722	White	COAD	NA	0.36	.
19:7685245-G/A	*XAB2*	19:7685245	White	UCEC	NA	0.345	.
19:7686072-G/A	*XAB2*	19:7686072	White	COAD	NA	0.338	.
19:7686072-G/A	*XAB2*	19:7686072	Asian	COAD	NA	0.338	.
19:7687526-G/A	*XAB2*	19:7687526	White	STAD	rs753191949	0.198	.
19:7688658-A/T	*XAB2*	19:7688658	AA/B	COAD	NA	0.487	.
19:7688658-A/T	*XAB2*	19:7688658	White	COAD	NA	0.487	.
19:7690852-C/T	*XAB2*	19:7690852	White	STAD	NA	0.373	.
19:7690852-C/T	*XAB2*	19:7690852	Asian	STAD	NA	0.373	.
19:7692145-T/C	*XAB2*	19:7692145	White	KIRC	NA	0.537	.
19:7692145-T/C	*XAB2*	19:7692145	AA/B	KIRC	NA	0.537	.
19:7692211-C/T	*XAB2*	19:7692211	Asian	COAD	rs201040075	0.551	.
19:7692211-C/T	*XAB2*	19:7692211	White	COAD	rs201040075	0.551	.
2:100017722-G/T	*REV1*	2:100017722	AA/B	SARC	NA	0.384	.

2:100017722-G/T	*REV1*	2:100017722	White	SARC	NA	0.384	.
2:100065819-C/T	*REV1*	2:100065819	White	UCEC	rs763086864	0.411	.
2:100079062-A/G	*REV1*	2:100079062	White	UCEC	NA	0.334	.
2:128028923-C/T	*ERCC3*	2:128028923	White	UCEC	rs1366074068	0.964	.
2:128028923-C/T	*ERCC3*	2:128028923	White	STAD	rs1366074068	0.964	.
2:128028923-C/T	*ERCC3*	2:128028923	Asian	STAD	rs1366074068	0.964	.
2:128028923-C/T	*ERCC3*	2:128028923	AA/B	UCEC	rs1366074068	0.964	.
2:128030517-G/A	*ERCC3*	2:128030517	White	STAD	NA	0.725	.
2:128030517-G/A	*ERCC3*	2:128030517	Asian	STAD	NA	0.725	.
2:128046300-C/A	*ERCC3*	2:128046300	Asian	UCEC	NA	0.388	.
2:128046300-C/A	*ERCC3*	2:128046300	Asian	UCEC	NA	0.691	.
2:128047329-C/T	*ERCC3*	2:128047329	White	UCEC	rs748503195	0.548	.
2:128047329-C/T	*ERCC3*	2:128047329	White	UCEC	rs748503195	0.787	.
2:136598413-C/T	*MCM6*	2:136598413	White	UCEC	rs200231978	0.122	.
2:136605718-T/A	*MCM6*	2:136605718	White	BRCA	NA	0.45	.
2:136605718-T/A	*MCM6*	2:136605718	AA/B	BRCA	NA	0.45	.
2:136614396-A/G	*MCM6*	2:136614396	White	STAD	NA	0.515	.
2:136614396-A/G	*MCM6*	2:136614396	Asian	STAD	NA	0.515	.
2:136616898-C/A	*MCM6*	2:136616898	White	UCEC	NA	0.473	.
2:136616905-A/G	*MCM6*	2:136616905	Asian	UCEC	NA	0.462	.
2:136616960-T/C	*MCM6*	2:136616960	Asian	STAD	NA	0.525	.
2:136616960-T/C	*MCM6*	2:136616960	White	STAD	NA	0.525	.
2:136616965-G/A	*MCM6*	2:136616965	Asian	UCEC	rs771453730	0.57	.

2:136623713-T/G	*MCM6*	2:136623713	Asian	STAD	NA	0.383	.
2:136623713-T/G	*MCM6*	2:136623713	White	STAD	NA	0.383	.
2:136624145-G/A	*MCM6*	2:136624145	Asian	STAD	rs769922696	0.277	.
2:136624145-G/A	*MCM6*	2:136624145	White	STAD	rs769922696	0.277	.
2:136630277-C/T	*MCM6*	2:136630277	Asian	STAD	NA	0.442	.
2:136630277-C/T	*MCM6*	2:136630277	White	STAD	NA	0.442	.
2:152273185-G/A	*RIF1*	2:152273185	AA/B	CESC	NA	0.186	.
2:152273185-G/A	*RIF1*	2:152273185	White	CESC	NA	0.186	.
2:152285384-G/T	*RIF1*	2:152285384	Asian	UCEC	NA	0.385	.
2:152285384-G/T	*RIF1*	2:152285384	Asian	UCEC	NA	0.238	.
2:152292002-C/T	*RIF1*	2:152292002	White	UCEC	rs182886629	0.284	.
2:152293377-G/A	*RIF1*	2:152293377	Asian	UCEC	rs550389711	0.142	.
2:152293377-G/A	*RIF1*	2:152293377	Asian	UCEC	rs550389711	0.11	.
2:152296613-C/G	*RIF1*	2:152296613	AA/B	OV	NA	0.312	.
2:152296613-C/G	*RIF1*	2:152296613	White	OV	NA	0.312	.
2:152296636-A/C	*RIF1*	2:152296636	White	STAD	NA	0.079	.
2:152296636-A/C	*RIF1*	2:152296636	White	STAD	NA	0.312	.
2:152298450-C/A	*RIF1*	2:152298450	White	UCEC	NA	0.416	.
2:152302991-G/T	*RIF1*	2:152302991	AA/B	SARC	NA	0.087	.
2:152302991-G/T	*RIF1*	2:152302991	AA/B	SARC	NA	0.531	.
2:152302991-G/T	*RIF1*	2:152302991	White	SARC	NA	0.087	.
2:152302991-G/T	*RIF1*	2:152302991	White	SARC	NA	0.531	.
2:152308139-G/T	*RIF1*	2:152308139	Asian	UCEC	NA	0.268	.

2:152308139-G/T	*RIF1*	2:152308139	White	STAD	NA	0.268	.
2:152311502-C/A	*RIF1*	2:152311502	White	UCEC	NA	0.259	.
2:152311502-C/A	*RIF1*	2:152311502	White	UCEC	NA	0.231	.
2:152311556-G/A	*RIF1*	2:152311556	White	UCEC	NA	0.534	.
2:152317719-A/G	*RIF1*	2:152317719	Asian	STAD	NA	0.333	.
2:152317719-A/G	*RIF1*	2:152317719	White	STAD	NA	0.333	.
2:152318785-T/G	*RIF1*	2:152318785	White	LUAD	NA	0.618	.
2:152318785-T/G	*RIF1*	2:152318785	AA/B	LUAD	NA	0.618	.
2:152319680-A/T	*RIF1*	2:152319680	White	STAD	NA	0.188	.
2:152319680-A/T	*RIF1*	2:152319680	Asian	STAD	NA	0.188	.
2:152320293-G/A	*RIF1*	2:152320293	White	CESC	rs751461918	0.159	.
2:152320293-G/A	*RIF1*	2:152320293	AA/B	CESC	rs751461918	0.159	.
2:152320295-C/T	*RIF1*	2:152320295	White	UCEC	rs754902148	0.174	.
2:152320410-A/C	*RIF1*	2:152320410	Asian	UCEC	NA	0.076	.
2:152320412-G/T	*RIF1*	2:152320412	White	STAD	NA	0.171	.
2:152320527-A/G	*RIF1*	2:152320527	White	STAD	NA	0.109	.
2:152320527-A/G	*RIF1*	2:152320527	Asian	STAD	NA	0.109	.
2:152320704-C/T	*RIF1*	2:152320704	Asian	UCEC	rs141807972	0.152	.
2:152320733-A/C	*RIF1*	2:152320733	White	LUAD	NA	0.133	.
2:152320733-A/C	*RIF1*	2:152320733	AA/B	LUAD	NA	0.133	.
2:152322492-C/A	*RIF1*	2:152322492	White	UCEC	NA	0.084	.
2:152325030-C/T	*RIF1*	2:152325030	White	UCEC	rs780970704	0.165	.
2:152326316-C/T	*RIF1*	2:152326316	White	BRCA	NA	0.484	.

2:152326316-C/T	*RIF1*	2:152326316	Asian	BRCA	NA	0.484	.
2:152331504-G/T	*RIF1*	2:152331504	White	COAD	NA	0.136	.
2:152331504-G/T	*RIF1*	2:152331504	Asian	COAD	NA	0.136	.
2:165404194-C/G	*GRB14*	2:165404194	AA/B	BRCA	rs1339598957	0.858	.
2:165404194-C/G	*GRB14*	2:165404194	White	BRCA	rs1339598957	0.858	.
2:17851778-C/T	*SMC6*	2:17851778	Asian	UCEC	NA	0.457	.
2:17864974-A/G	*SMC6*	2:17864974	Asian	COAD	NA	0.3	.
2:17864974-A/G	*SMC6*	2:17864974	White	COAD	NA	0.3	.
2:17881495-C/G	*SMC6*	2:17881495	White	CESC	NA	0.08	.
2:17881495-C/G	*SMC6*	2:17881495	AA/B	CESC	NA	0.08	.
2:17896164-G/A	*SMC6*	2:17896164	White	BRCA	NA	0.255	.
2:17896164-G/A	*SMC6*	2:17896164	AA/B	BRCA	NA	0.255	.
2:17896206-C/A	*SMC6*	2:17896206	White	UCEC	NA	0.091	.
2:17906609-G/A	*SMC6*	2:17906609	AA/B	COAD	rs760703737	0.424	.
2:17906609-G/A	*SMC6*	2:17906609	White	COAD	rs760703737	0.424	.
2:17913124-G/A	*SMC6*	2:17913124	White	LUSC	NA	0.162	.
2:17913124-G/A	*SMC6*	2:17913124	AA/B	LUSC	NA	0.162	.
2:17919558-C/T	*SMC6*	2:17919558	White	UCEC	NA	0.37	.
2:17941274-C/T	*GEN1*	2:17941274	White	UCEC	NA	0.488	.
2:17942779-G/A	*GEN1*	2:17942779	Asian	UCEC	rs777769429	0.356	.
2:17946183-G/T	*GEN1*	2:17946183	White	SARC	NA	0.099	.
2:17946183-G/T	*GEN1*	2:17946183	AA/B	SARC	NA	0.099	.
2:17946302-G/A	*GEN1*	2:17946302	White	UCEC	NA	0.76	.

2:17950042-T/G	*GEN1*	2:17950042	White	UCEC	NA	0.756	.
2:17952495-C/A	*GEN1*	2:17952495	White	UCEC	NA	0.089	.
2:17962800-C/A	*GEN1*	2:17962800	White	UCEC	NA	0.061	.
2:190708723-C/T	*PMS1*	2:190708723	White	UCEC	rs758149651	0.422	.
2:190718672-G/A	*PMS1*	2:190718672	White	STAD	rs773928881	0.622	.
2:190718672-G/A	*PMS1*	2:190718672	White	BRCA	rs773928881	0.622	.
2:190718672-G/A	*PMS1*	2:190718672	AA/B	BRCA	rs773928881	0.622	.
2:190718672-G/A	*PMS1*	2:190718672	Asian	STAD	rs773928881	0.622	.
2:190719671-G/T	*PMS1*	2:190719671	AA/B	LUAD	NA	0.657	.
2:190719671-G/T	*PMS1*	2:190719671	White	LUAD	NA	0.657	.
2:190728815-G/A	*PMS1*	2:190728815	Asian	UCEC	NA	0.071	.
2:190732626-C/T	*PMS1*	2:190732626	White	UCEC	rs139826569	0.56	.
2:190742011-G/A	*PMS1*	2:190742011	White	STAD	rs377603311	0.781	.
2:190742011-G/A	*PMS1*	2:190742011	Asian	STAD	rs377603311	0.781	.
2:206870035-C/G	*INO80D*	2:206870035	AA/B	KIRC	NA	0.185	.
2:206870035-C/G	*INO80D*	2:206870035	White	KIRC	NA	0.185	.
2:206872095-G/T	*INO80D*	2:206872095	AA/B	BRCA	NA	0.289	.
2:206872095-G/T	*INO80D*	2:206872095	White	BRCA	NA	0.289	.
2:206920937-G/T	*INO80D*	2:206920937	White	UCEC	NA	0.187	.
2:206927685-G/A	*INO80D*	2:206927685	White	STAD	NA	0.401	.
2:206927685-G/A	*INO80D*	2:206927685	Asian	STAD	NA	0.401	.
2:215593615-G/A	*BARD1*	2:215593615	Asian	STAD	NA	0.537	.
2:215593615-G/A	*BARD1*	2:215593615	White	STAD	NA	0.537	.

2:216992348-C/G	*XRCC5*	2:216992348	Asian	STAD	NA	0.294	.
2:216992348-C/G	*XRCC5*	2:216992348	White	STAD	NA	0.294	.
2:216995653-G/T	*XRCC5*	2:216995653	White	COAD	NA	0.279	.
2:217069114-G/A	*XRCC5*	2:217069114	White	COAD	rs772117253	0.335	.
2:217297484-A/G	*SMARCAL1*	2:217297484	Asian	STAD	NA	0.949	.
2:217297484-A/G	*SMARCAL1*	2:217297484	White	STAD	NA	0.949	.
2:217311862-G/A	*SMARCAL1*	2:217311862	White	UCEC	rs753996369	0.931	.
2:217341886-G/T	*SMARCAL1*	2:217341886	AA/B	KIRP	NA	0.383	.
2:217341886-G/T	*SMARCAL1*	2:217341886	White	KIRP	NA	0.383	.
2:219537590-G/A	*STK36*	2:219537590	White	UCEC	NA	0.932	.
2:219540121-G/A	*STK36*	2:219540121	Asian	STAD	rs770755293	0.585	.
2:219540121-G/A	*STK36*	2:219540121	White	STAD	rs770755293	0.585	.
2:219553429-G/T	*STK36*	2:219553429	AA/B	CESC	NA	0.459	.
2:219553429-G/T	*STK36*	2:219553429	White	CESC	NA	0.459	.
2:219557396-C/G	*STK36*	2:219557396	Asian	BRCA	NA	0.402	.
2:219557396-C/G	*STK36*	2:219557396	White	BRCA	NA	0.402	.
2:220022258-C/T	*NHEJ1*	2:220022258	Asian	UCEC	rs777365329	0.039	.
2:225339039-G/A	*CUL3*	2:225339039	White	UCEC	rs752958265	0.304	.
2:225339039-G/A	*CUL3*	2:225339039	White	UCEC	rs752958265	0.399	.
2:225342967-G/A	*CUL3*	2:225342967	White	STAD	NA	0.88	.
2:225342967-G/A	*CUL3*	2:225342967	Asian	STAD	NA	0.88	.
2:225365164-G/A	*CUL3*	2:225365164	AA/B	COAD	NA	0.841	.
2:225365164-G/A	*CUL3*	2:225365164	White	COAD	NA	0.841	.

2:225379426-G/C	*CUL3*	2:225379426	AA/B	COAD	NA	0.695	.
2:225379426-G/C	*CUL3*	2:225379426	AA/B	COAD	NA	0.194	.
2:225379426-G/C	*CUL3*	2:225379426	White	COAD	NA	0.695	.
2:225379426-G/C	*CUL3*	2:225379426	White	COAD	NA	0.194	.
2:225400349-C/G	*CUL3*	2:225400349	White	OV	NA	0.219	.
2:225400349-C/G	*CUL3*	2:225400349	White	OV	NA	0.284	.
2:225400349-C/G	*CUL3*	2:225400349	AA/B	OV	NA	0.219	.
2:225400349-C/G	*CUL3*	2:225400349	AA/B	OV	NA	0.284	.
2:225422486-G/A	*CUL3*	2:225422486	Asian	ESCA	NA	0.638	.
2:225422486-G/A	*CUL3*	2:225422486	Asian	ESCA	NA	0.347	.
2:225422486-G/A	*CUL3*	2:225422486	White	ESCA	NA	0.638	.
2:225422486-G/A	*CUL3*	2:225422486	White	ESCA	NA	0.347	.
2:231222653-C/T	*SP140L*	2:231222653	Asian	UCEC	rs189762003	0.278	.
2:231236364-G/T	*SP140L*	2:231236364	Asian	UCEC	NA	0.204	.
2:231256883-G/T	*SP140L*	2:231256883	White	LUAD	NA	0.381	.
2:231256883-G/T	*SP140L*	2:231256883	AA/B	LUAD	NA	0.381	.
2:231256909-C/T	*SP140L*	2:231256909	White	UCEC	rs61750011	0.335	.
2:231264899-G/A	*SP140L*	2:231264899	White	HNSC	rs747016700	0.304	.
2:231266421-G/C	*SP140L*	2:231266421	Asian	ESCA	NA	0.121	.
2:231266421-G/C	*SP140L*	2:231266421	White	ESCA	NA	0.121	.
2:25457180-C/T	*DNMT3A*	2:25457180	White	UCEC	rs749167103	0.696	.
2:25457215-C/T	*DNMT3A*	2:25457215	AA/B	COAD	NA	0.798	.
2:25457215-C/T	*DNMT3A*	2:25457215	White	COAD	NA	0.798	.

2:25463181-C/T	*DNMT3A*	2:25463181	Asian	UCEC	rs757823678	0.904	.
2:25467457-C/T	*DNMT3A*	2:25467457	White	LGG	NA	0.955	.
2:25467457-C/T	*DNMT3A*	2:25467457	AA/B	LGG	NA	0.955	.
2:25469531-C/T	*DNMT3A*	2:25469531	AA/B	LUAD	NA	0.806	.
2:25469531-C/T	*DNMT3A*	2:25469531	White	LUAD	NA	0.806	.
2:25470582-C/A	*DNMT3A*	2:25470582	Asian	UCEC	NA	0.871	.
2:28117484-G/A	*BRE*	2:28117484	AA/B	BRCA	rs1445947949	0.204	.
2:28210901-G/A	*BRE*	2:28210901	Asian	STAD	2:28210901_G/A	0.419	.
2:28210901-G/A	*BRE*	2:28210901	White	STAD	2:28210901_G/A	0.419	.
2:28210901-G/A	*BRE*	2:28210901	Asian	UCEC	2:28210901_G/A	0.419	.
2:47637393-G/A	*MSH2*	2:47637393	Asian	STAD	NA	0.905	.
2:47637393-G/A	*MSH2*	2:47637393	White	STAD	NA	0.905	.
2:47639563-G/T	*MSH2*	2:47639563	White	UCEC	NA	0.875	.
2:47643493-A/C	*MSH2*	2:47643493	White	COAD	NA	0.779	.
2:47643546-G/T	*MSH2*	2:47643546	White	UCEC	rs1465121242	0.93	.
2:47693856-C/T	*MSH2*	2:47693856	White	UCEC	rs755818010	0.853	.
2:47702214-G/A	*MSH2*	2:47702214	Asian	ESCA	NA	0.844	.
2:47702214-G/A	*MSH2*	2:47702214	White	ESCA	NA	0.844	.
2:47703590-G/A	*MSH2*	2:47703590	AA/B	COAD	NA	0.854	uncertain_significance,pathogenic
2:47703590-G/A	*MSH2*	2:47703590	White	COAD	NA	0.854	uncertain_significance,pathogenic
2:47705415-G/A	*MSH2*	2:47705415	Asian	COAD	NA	0.925	.

2:47705415-G/A	*MSH2*	2:47705415	White	COAD	NA	0.925	.
2:47709954-G/A	*MSH2*	2:47709954	White	STAD	NA	0.703	.
2:48018102-G/T	*MSH6*	2:48018102	Asian	UCEC	NA	0.64	uncertain_significance
2:48026261-A/C	*MSH6*	2:48026261	Asian	UCEC	NA	0.84	.
2:48026449-G/A	*MSH6*	2:48026449	AA/B	BRCA	NA	0.959	.
2:48026449-G/A	*MSH6*	2:48026449	White	BRCA	NA	0.959	.
2:48027125-C/G	*MSH6*	2:48027125	AA/B	KIRP	NA	0.691	.
2:48027125-C/G	*MSH6*	2:48027125	White	KIRP	NA	0.691	.
2:48027198-A/C	*MSH6*	2:48027198	White	BRCA	NA	0.693	.
2:48027198-A/C	*MSH6*	2:48027198	AA/B	BRCA	NA	0.693	.
2:48028049-G/A	*MSH6*	2:48028049	Asian	STAD	NA	0.857	uncertain_significance
2:48028049-G/A	*MSH6*	2:48028049	White	STAD	NA	0.857	uncertain_significance
2:48030583-A/G	*MSH6*	2:48030583	White	ESCA	rs372103816	0.83	uncertain_significance
2:48030583-A/G	*MSH6*	2:48030583	Asian	ESCA	rs372103816	0.83	uncertain_significance
2:48030613-G/A	*MSH6*	2:48030613	White	UCEC	rs779617676	0.839	uncertain_significance
2:48033419-T/G	*MSH6*	2:48033419	White	COAD	NA	0.735	.
2:48033419-T/G	*MSH6*	2:48033419	AA/B	COAD	NA	0.735	.

2:58392928-C/T	*FANCL*	2:58392928	Asian	UCEC	rs199564543	0.699	.
2:58392928-C/T	*FANCL*	2:58392928	Asian	UCEC	rs199564543	0.772	.
2:58425763-A/C	*FANCL*	2:58425763	AA/B	COAD	NA	0.303	.
2:58425763-A/C	*FANCL*	2:58425763	AA/B	COAD	NA	0.494	.
2:58425763-A/C	*FANCL*	2:58425763	White	COAD	NA	0.303	.
2:58425763-A/C	*FANCL*	2:58425763	White	COAD	NA	0.494	.
2:58468421-G/A	*FANCL*	2:58468421	White	COAD	rs146690827	0.24	.
2:58468421-G/A	*FANCL*	2:58468421	White	COAD	rs146690827	0.262	.
2:58468421-G/A	*FANCL*	2:58468421	AA/B	COAD	rs146690827	0.24	.
2:58468421-G/A	*FANCL*	2:58468421	AA/B	COAD	rs146690827	0.262	.
2:68729914-C/A	*APLF*	2:68729914	Asian	STAD	NA	0.168	.
2:68729914-C/A	*APLF*	2:68729914	White	STAD	NA	0.168	.
2:68804999-G/C	*APLF*	2:68804999	AA/B	CESC	rs1221229078	0.125	.
2:68804999-G/C	*APLF*	2:68804999	White	CESC	rs1221229078	0.125	.
2:68805110-G/A	*APLF*	2:68805110	AA/B	BRCA	rs755954160	0.073	.
2:68805110-G/A	*APLF*	2:68805110	White	BRCA	rs755954160	0.073	.
2:68805150-A/C	*APLF*	2:68805150	Asian	UCEC	NA	0.064	.
2:7018173-G/A	*RSAD2*	2:7018173	White	COAD	rs751694276	0.782	.
2:7023509-G/T	*RSAD2*	2:7023509	Asian	UCEC	rs1258495440	0.59	.
2:7023572-G/T	*RSAD2*	2:7023572	White	THCA	NA	0.777	.
2:7023572-G/T	*RSAD2*	2:7023572	Asian	THCA	NA	0.777	.
2:7027129-T/C	*RSAD2*	2:7027129	AA/B	LUSC	NA	0.968	.
2:7027129-T/C	*RSAD2*	2:7027129	White	LUSC	NA	0.968	.

2:7027234-G/A	*RSAD2*	2:7027234	AA/B	COAD	rs753068844	0.56	.
2:7027234-G/A	*RSAD2*	2:7027234	White	COAD	rs753068844	0.56	.
2:7036014-T/A	*RSAD2*	2:7036014	Asian	UCEC	NA	0.604	.
20:3778375-T/C	*CDC25B*	20:3778375	White	COAD	NA	0.162	.
20:3778396-G/T	*CDC25B*	20:3778396	White	LUAD	rs1488303200	0.434	.
20:3778396-G/T	*CDC25B*	20:3778396	AA/B	LUAD	rs1488303200	0.434	.
20:3781144-C/T	*CDC25B*	20:3781144	White	STAD	rs775666911	0.353	.
20:3781144-C/T	*CDC25B*	20:3781144	Asian	STAD	rs775666911	0.353	.
20:3783576-T/C	*CDC25B*	20:3783576	Asian	COAD	NA	0.172	.
20:3783576-T/C	*CDC25B*	20:3783576	White	COAD	NA	0.172	.
20:3783819-G/C	*CDC25B*	20:3783819	AA/B	LIHC	NA	0.919	.
20:3784183-A/G	*CDC25B*	20:3784183	AA/B	COAD	NA	0.547	.
20:3784183-A/G	*CDC25B*	20:3784183	White	COAD	NA	0.547	.
20:55917885-T/C	*SPO11*	20:55917885	White	COAD	NA	0.67	.
20:55918512-T/C	*SPO11*	20:55918512	White	STAD	rs746992170	0.423	.
20:55918512-T/C	*SPO11*	20:55918512	Asian	STAD	rs746992170	0.423	.
20:62292678-A/G	*RTEL1*	20:62292678	AA/B	CESC	NA	0.836	.
20:62292678-A/G	*RTEL1*	20:62292678	White	CESC	NA	0.836	.
20:62293281-G/A	*RTEL1*	20:62293281	White	UCEC	rs769163032	0.261	.
20:62320953-G/T	*RTEL1*	20:62320953	White	COAD	NA	0.85	.
20:62326186-C/T	*RTEL1*	20:62326186	AA/B	BRCA	rs762640318	0.319	.
20:62326186-C/T	*RTEL1*	20:62326186	White	BRCA	rs762640318	0.319	.
21:42807920-G/C	*MX1*	21:42807920	Asian	BRCA	NA	0.965	.

21:42807920-G/C	*MX1*	21:42807920	White	BRCA	NA	0.965	.
21:42807927-T/G	*MX1*	21:42807927	White	COAD	NA	0.971	.
21:42807927-T/G	*MX1*	21:42807927	AA/B	COAD	NA	0.971	.
21:42830560-G/C	*MX1*	21:42830560	White	BRCA	NA	0.268	.
21:42830560-G/C	*MX1*	21:42830560	AA/B	BRCA	NA	0.268	.
22:19422280-G/T	*MRPL40*	22:19422280	Asian	UCEC	NA	0.317	.
22:22312960-A/C	*TOP3B*	22:22312960	White	UCEC	NA	0.511	.
22:22316801-C/T	*TOP3B*	22:22316801	White	ESCA	rs762892315	0.92	.
22:22316801-C/T	*TOP3B*	22:22316801	Asian	ESCA	rs762892315	0.92	.
22:22316924-G/A	*TOP3B*	22:22316924	White	COAD	rs776734531	0.427	.
22:22316924-G/A	*TOP3B*	22:22316924	AA/B	COAD	rs776734531	0.427	.
22:22319678-G/A	*TOP3B*	22:22319678	AA/B	COAD	rs769874024	0.514	.
22:22319678-G/A	*TOP3B*	22:22319678	White	COAD	rs769874024	0.514	.
22:22324736-C/A	*TOP3B*	22:22324736	AA/B	LUAD	rs1488574727	0.093	.
22:22324736-C/A	*TOP3B*	22:22324736	White	LUAD	rs1488574727	0.093	.
22:22892465-C/G	*PRAME*	22:22892465	White	LUAD	NA	0.146	.
22:22892465-C/G	*PRAME*	22:22892465	AA/B	LUAD	NA	0.146	.
22:22892728-G/A	*PRAME*	22:22892728	Asian	STAD	rs751654581	0.212	.
22:22892728-G/A	*PRAME*	22:22892728	Asian	STAD	rs751654581	0.15	.
22:22892728-G/A	*PRAME*	22:22892728	White	STAD	rs751654581	0.212	.
22:22892728-G/A	*PRAME*	22:22892728	White	STAD	rs751654581	0.15	.
22:22893364-A/C	*PRAME*	22:22893364	White	LUSC	rs769323726	0.225	.
22:22893364-A/C	*PRAME*	22:22893364	White	LUSC	rs769323726	0.128	.

22:22893364-A/C	*PRAME*	22:22893364	AA/B	LUSC	rs769323726	0.225	.
22:22893364-A/C	*PRAME*	22:22893364	AA/B	LUSC	rs769323726	0.128	.
22:29091738-C/T	*CHEK2*	22:29091738	White	SARC	NA	0.147	.
22:29091738-C/T	*CHEK2*	22:29091738	AA/B	SARC	NA	0.147	.
22:29091838-C/G	*CHEK2*	22:29091838	White	CESC	NA	0.14	.
22:29091838-C/G	*CHEK2*	22:29091838	White	CESC	NA	0.307	.
22:29091838-C/G	*CHEK2*	22:29091838	AA/B	CESC	NA	0.14	.
22:29091838-C/G	*CHEK2*	22:29091838	AA/B	CESC	NA	0.307	.
22:29099509-A/C	*CHEK2*	22:29099509	Asian	UCEC	NA	0.551	.
22:29099509-A/C	*CHEK2*	22:29099509	Asian	UCEC	NA	0.901	.
22:29106013-A/T	*CHEK2*	22:29106013	White	BRCA	NA	0.36	.
22:29106013-A/T	*CHEK2*	22:29106013	White	BRCA	NA	0.748	.
22:29106013-A/T	*CHEK2*	22:29106013	AA/B	BRCA	NA	0.36	.
22:29106013-A/T	*CHEK2*	22:29106013	AA/B	BRCA	NA	0.748	.
22:29107986-T/G	*CHEK2*	22:29107986	White	COAD	NA	0.222	.
22:29115390-G/A	*CHEK2*	22:29115390	AA/B	SARC	NA	0.537	.
22:29115390-G/A	*CHEK2*	22:29115390	White	SARC	NA	0.537	.
22:29121000-T/C	*CHEK2*	22:29121000	Asian	THCA	rs369223840	0.61	uncertain_significance
22:29121000-T/C	*CHEK2*	22:29121000	White	THCA	rs369223840	0.61	uncertain_significance
22:29121073-C/T	*CHEK2*	22:29121073	White	UCEC	NA	0.82	.

22:29121241-C/T	*CHEK2*	22:29121241	AA/B	COAD	rs587781667	0.678	uncertain_significance
22:29121241-C/T	*CHEK2*	22:29121241	White	COAD	rs587781667	0.678	uncertain_significance
22:29121246-G/C	*CHEK2*	22:29121246	Asian	THCA	NA	0.915	.
22:29121246-G/C	*CHEK2*	22:29121246	White	THCA	NA	0.915	.
22:36537873-C/G	*APOL3*	22:36537873	White	ESCA	NA	0.19	.
22:36537873-C/G	*APOL3*	22:36537873	Asian	ESCA	NA	0.19	.
22:36537881-G/T	*APOL3*	22:36537881	AA/B	LUAD	rs537640724	0.076	.
22:36537881-G/T	*APOL3*	22:36537881	White	LUAD	rs537640724	0.076	.
22:38363649-T/C	*POLR2F*	22:38363649	White	COAD	NA	0.878	.
22:38935337-C/T	*DMC1*	22:38935337	Asian	STAD	rs1282071888	0.674	.
22:38935337-C/T	*DMC1*	22:38935337	White	STAD	rs1282071888	0.674	.
22:42024230-T/C	*XRCC6*	22:42024230	White	UCEC	NA	0.458	.
22:42032677-G/C	*XRCC6*	22:42032677	Asian	BRCA	NA	0.366	.
22:42032677-G/C	*XRCC6*	22:42032677	White	BRCA	NA	0.366	.
22:50695537-C/T	*MAPK12*	22:50695537	White	CESC	rs750343193	0.404	.
22:50695537-C/T	*MAPK12*	22:50695537	AA/B	CESC	rs750343193	0.404	.
22:50987379-C/T	*KLHDC7B*	22:50987379	White	COAD	rs546175063	0.242	.
22:50987379-C/T	*KLHDC7B*	22:50987379	AA/B	COAD	rs546175063	0.242	.
22:50987832-G/A	*KLHDC7B*	22:50987832	Asian	UCEC	NA	0.459	.
22:50987889-G/A	*KLHDC7B*	22:50987889	White	STAD	rs371425484	0.468	.
22:50987889-G/A	*KLHDC7B*	22:50987889	Asian	STAD	rs371425484	0.468	.

22:50987890-C/T	*KLHDC7B*	22:50987890	White	CESC	NA	0.539	.
22:50987890-C/T	*KLHDC7B*	22:50987890	AA/B	CESC	NA	0.539	.
22:50988099-C/T	*KLHDC7B*	22:50988099	Asian	STAD	rs758075611	0.267	.
22:50988099-C/T	*KLHDC7B*	22:50988099	White	STAD	rs758075611	0.267	.
22:50988123-C/T	*KLHDC7B*	22:50988123	White	STAD	rs750273973	0.2	.
22:50988123-C/T	*KLHDC7B*	22:50988123	Asian	STAD	rs750273973	0.2	.
3:10078003-G/T	*FANCD2*	3:10078003	Asian	STAD	NA	0.063	.
3:10078003-G/T	*FANCD2*	3:10078003	White	STAD	NA	0.063	.
3:10081483-G/A	*FANCD2*	3:10081483	White	STAD	NA	0.488	.
3:10081483-G/A	*FANCD2*	3:10081483	Asian	STAD	NA	0.488	.
3:10107101-C/T	*FANCD2*	3:10107101	AA/B	BRCA	NA	0.194	.
3:10107101-C/T	*FANCD2*	3:10107101	White	BRCA	NA	0.194	.
3:10108924-C/A	*FANCD2*	3:10108924	White	KIRP	NA	0.187	.
3:10108924-C/A	*FANCD2*	3:10108924	AA/B	KIRP	NA	0.187	.
3:10114587-T/A	*FANCD2*	3:10114587	AA/B	BRCA	NA	0.19	.
3:10114587-T/A	*FANCD2*	3:10114587	White	BRCA	NA	0.19	.
3:121168239-T/G	*POLQ*	3:121168239	Asian	UCEC	NA	0.792	.
3:121190857-G/A	*POLQ*	3:121190857	AA/B	CESC	NA	0.359	.
3:121190857-G/A	*POLQ*	3:121190857	White	CESC	NA	0.359	.
3:121190872-C/A	*POLQ*	3:121190872	White	COAD	NA	0.518	.
3:121190872-C/A	*POLQ*	3:121190872	Asian	COAD	NA	0.518	.
3:121192268-G/T	*POLQ*	3:121192268	White	UCEC	NA	0.212	.
3:121192292-T/G	*POLQ*	3:121192292	White	UCEC	NA	0.138	.

3:121200433-T/A	*POLQ*	3:121200433	White	LUAD	NA	0.326	.
3:121200433-T/A	*POLQ*	3:121200433	AA/B	LUAD	NA	0.326	.
3:121206221-A/T	*POLQ*	3:121206221	AA/B	BRCA	NA	0.208	.
3:121206221-A/T	*POLQ*	3:121206221	White	BRCA	NA	0.208	.
3:121209250-C/T	*POLQ*	3:121209250	White	UCEC	NA	0.169	.
3:121215700-G/A	*POLQ*	3:121215700	White	STAD	rs373455498	0.456	.
3:121217345-C/T	*POLQ*	3:121217345	White	KIRC	NA	0.18	.
3:121217345-C/T	*POLQ*	3:121217345	AA/B	KIRC	NA	0.18	.
3:121217409-C/T	*POLQ*	3:121217409	White	BRCA	NA	0.212	.
3:121217409-C/T	*POLQ*	3:121217409	AA/B	BRCA	NA	0.212	.
3:121238807-C/T	*POLQ*	3:121238807	White	STAD	rs763394640	0.655	.
3:121240856-G/C	*POLQ*	3:121240856	White	CESC	NA	0.634	.
3:121240856-G/C	*POLQ*	3:121240856	AA/B	CESC	NA	0.634	.
3:121248537-C/A	*POLQ*	3:121248537	Asian	UCEC	NA	0.507	.
3:121258349-C/T	*POLQ*	3:121258349	White	STAD	NA	0.21	.
3:121258349-C/T	*POLQ*	3:121258349	Asian	STAD	NA	0.21	.
3:121264612-A/C	*POLQ*	3:121264612	AA/B	LUSC	NA	0.037	.
3:121264612-A/C	*POLQ*	3:121264612	White	LUSC	NA	0.037	.
3:127323573-G/A	*MCM2*	3:127323573	White	UCEC	rs755140598	0.607	.
3:127327751-T/A	*MCM2*	3:127327751	White	STAD	NA	0.603	.
3:127327751-T/A	*MCM2*	3:127327751	White	STAD	NA	0.966	.
3:127327751-T/A	*MCM2*	3:127327751	Asian	STAD	NA	0.603	.
3:127327751-T/A	*MCM2*	3:127327751	Asian	STAD	NA	0.966	.

3:127335861-C/T	*MCM2*	3:127335861	White	STAD	rs768309904	0.341	.
3:127335861-C/T	*MCM2*	3:127335861	White	STAD	rs768309904	0.582	.
3:127335861-C/T	*MCM2*	3:127335861	Asian	STAD	rs768309904	0.341	.
3:127335861-C/T	*MCM2*	3:127335861	Asian	STAD	rs768309904	0.582	.
3:127339709-C/T	*MCM2*	3:127339709	White	STAD	rs766565920	0.222	.
3:127339709-C/T	*MCM2*	3:127339709	Asian	STAD	rs766565920	0.222	.
3:127339709-C/T	*MCM2*	3:127339709	White	UCEC	rs766565920	0.222	.
3:127340541-T/G	*MCM2*	3:127340541	Asian	UCEC	NA	0.238	.
3:127340541-T/G	*MCM2*	3:127340541	Asian	UCEC	NA	0.26	.
3:129151945-G/T	*MBD4*	3:129151945	White	COAD	NA	0.617	.
3:129151968-G/A	*MBD4*	3:129151968	Asian	COAD	rs1381175027	0.882	.
3:129151968-G/A	*MBD4*	3:129151968	White	COAD	rs1381175027	0.882	.
3:129152019-C/G	*MBD4*	3:129152019	White	BRCA	NA	0.692	.
3:129152019-C/G	*MBD4*	3:129152019	Asian	BRCA	NA	0.692	.
3:133335685-C/G	*TOPBP1*	3:133335685	White	BRCA	rs1156859173	0.692	.
3:133335685-C/G	*TOPBP1*	3:133335685	AA/B	BRCA	rs1156859173	0.692	.
3:133339033-G/A	*TOPBP1*	3:133339033	Asian	STAD	rs751699737	0.34	.
3:133339033-G/A	*TOPBP1*	3:133339033	White	STAD	rs751699737	0.34	.
3:133339083-C/A	*TOPBP1*	3:133339083	Asian	UCEC	NA	0.21	.
3:133342953-C/A	*TOPBP1*	3:133342953	White	UCEC	NA	0.553	.
3:133347225-A/G	*TOPBP1*	3:133347225	White	COAD	NA	0.645	.
3:133347225-A/G	*TOPBP1*	3:133347225	AA/B	COAD	NA	0.645	.
3:133356736-G/T	*TOPBP1*	3:133356736	White	UCEC	NA	0.236	.

3:133358853-C/T	*TOPBP1*	3:133358853	AA/B	BRCA	NA	0.521	.
3:133358853-C/T	*TOPBP1*	3:133358853	White	BRCA	NA	0.521	.
3:133358952-A/C	*TOPBP1*	3:133358952	Asian	UCEC	NA	0.973	.
3:133358955-T/C	*TOPBP1*	3:133358955	Asian	STAD	NA	0.956	.
3:133358955-T/C	*TOPBP1*	3:133358955	White	STAD	NA	0.956	.
3:133363129-G/T	*TOPBP1*	3:133363129	White	UCEC	NA	0.044	.
3:133372215-G/T	*TOPBP1*	3:133372215	White	UCEC	NA	0.296	.
3:133372215-G/T	*TOPBP1*	3:133372215	White	UCEC	NA	0.306	.
3:133372333-G/T	*TOPBP1*	3:133372333	White	BRCA	NA	0.356	.
3:133372333-G/T	*TOPBP1*	3:133372333	Asian	BRCA	NA	0.356	.
3:133372361-C/G	*TOPBP1*	3:133372361	White	STAD	NA	0.311	.
3:133372361-C/G	*TOPBP1*	3:133372361	White	STAD	NA	0.807	.
3:133372361-C/G	*TOPBP1*	3:133372361	Asian	STAD	NA	0.311	.
3:133372361-C/G	*TOPBP1*	3:133372361	Asian	STAD	NA	0.807	.
3:133374172-A/C	*TOPBP1*	3:133374172	White	COAD	NA	0.433	.
3:133374172-A/C	*TOPBP1*	3:133374172	Asian	COAD	NA	0.433	.
3:133376643-C/T	*TOPBP1*	3:133376643	White	UCEC	rs778316444	0.429	.
3:14188850-C/A	*XPC*	3:14188850	Asian	UCEC	NA	0.815	.
3:14190063-C/T	*XPC*	3:14190063	White	UCEC	rs745660242	0.217	.
3:14190105-C/T	*XPC*	3:14190105	AA/B	BRCA	rs766730833	0.765	.
3:14190105-C/T	*XPC*	3:14190105	White	BRCA	rs766730833	0.765	.
3:14190340-G/C	*XPC*	3:14190340	AA/B	LUAD	NA	0.359	.
3:14190340-G/C	*XPC*	3:14190340	White	LUAD	NA	0.359	.

3:14201311-C/T	*XPC*	3:14201311	White	UCEC	rs758687221	0.483	.
3:14208706-T/G	*XPC*	3:14208706	White	COAD	NA	0.263	.
3:142172056-G/T	*ATR*	3:142172056	White	ESCA	NA	0.758	.
3:142172056-G/T	*ATR*	3:142172056	White	ESCA	NA	0.668	.
3:142172056-G/T	*ATR*	3:142172056	Asian	ESCA	NA	0.758	.
3:142172056-G/T	*ATR*	3:142172056	Asian	ESCA	NA	0.668	.
3:142184050-C/G	*ATR*	3:142184050	AA/B	CESC	NA	0.725	.
3:142184050-C/G	*ATR*	3:142184050	AA/B	CESC	NA	0.758	.
3:142184050-C/G	*ATR*	3:142184050	White	CESC	NA	0.725	.
3:142184050-C/G	*ATR*	3:142184050	White	CESC	NA	0.758	.
3:142185258-T/C	*ATR*	3:142185258	White	UCEC	NA	0.885	.
3:142185258-T/C	*ATR*	3:142185258	White	UCEC	NA	0.532	.
3:142215271-G/A	*ATR*	3:142215271	White	COAD	NA	0.748	.
3:142215271-G/A	*ATR*	3:142215271	AA/B	COAD	NA	0.748	.
3:142238539-C/T	*ATR*	3:142238539	White	UCEC	NA	0.338	.
3:142254967-G/A	*ATR*	3:142254967	White	COAD	NA	0.371	.
3:142257424-C/A	*ATR*	3:142257424	AA/B	LUAD	rs1250182269	0.692	.
3:142257424-C/A	*ATR*	3:142257424	White	LUAD	rs1250182269	0.692	.
3:142259752-C/T	*ATR*	3:142259752	White	BRCA	NA	0.446	.
3:142259752-C/T	*ATR*	3:142259752	AA/B	BRCA	NA	0.446	.
3:142274948-C/A	*ATR*	3:142274948	Asian	UCEC	NA	0.15	.
3:148757389-G/A	*HLTF*	3:148757389	White	UCEC	NA	0.412	.
3:148759374-C/T	*HLTF*	3:148759374	White	UCEC	NA	0.858	.

3:148759431-C/A	*HLTF*	3:148759431	Asian	UCEC	NA	0.437	.
3:148768095-C/T	*HLTF*	3:148768095	Asian	UCEC	rs1210280419	0.853	.
3:148789064-C/T	*HLTF*	3:148789064	AA/B	LUSC	rs768864078	0.928	.
3:148789064-C/T	*HLTF*	3:148789064	White	LUSC	rs768864078	0.928	.
3:148789103-T/A	*HLTF*	3:148789103	White	UCEC	NA	0.753	.
3:148789221-T/G	*HLTF*	3:148789221	White	STAD	NA	0.819	.
3:148793778-C/A	*HLTF*	3:148793778	Asian	UCEC	NA	0.481	.
3:186510722-G/A	*RFC4*	3:186510722	White	COAD	NA	0.672	.
3:186510722-G/A	*RFC4*	3:186510722	AA/B	COAD	NA	0.672	.
3:193854426-G/T	*HES1*	3:193854426	White	UCEC	NA	0.923	.
3:193854799-T/C	*HES1*	3:193854799	White	ESCA	NA	0.958	.
3:193854799-T/C	*HES1*	3:193854799	Asian	ESCA	NA	0.958	.
3:193855904-G/A	*HES1*	3:193855904	White	COAD	NA	0.243	.
3:193855904-G/A	*HES1*	3:193855904	Asian	COAD	NA	0.243	.
3:196214404-T/C	*RNF168*	3:196214404	Asian	UCEC	NA	0.155	.
3:25656736-A/C	*TOP2B*	3:25656736	Asian	BRCA	NA	0.852	.
3:25656736-A/C	*TOP2B*	3:25656736	White	BRCA	NA	0.852	.
3:25657005-C/A	*TOP2B*	3:25657005	White	STAD	NA	0.337	.
3:25657005-C/A	*TOP2B*	3:25657005	Asian	STAD	NA	0.337	.
3:25660275-G/T	*TOP2B*	3:25660275	Asian	UCEC	NA	0.602	.
3:25665077-G/A	*TOP2B*	3:25665077	AA/B	LIHC	NA	0.62	.
3:25665815-C/T	*TOP2B*	3:25665815	Asian	UCEC	NA	0.572	.
3:25668727-C/T	*TOP2B*	3:25668727	White	PAAD	rs757502615	0.514	.

3:25668727-C/T	*TOP2B*	3:25668727	White	UCEC	rs757502615	0.514	.
3:25668727-C/T	*TOP2B*	3:25668727	AA/B	COAD	rs757502615	0.514	.
3:25668727-C/T	*TOP2B*	3:25668727	Asian	PAAD	rs757502615	0.514	.
3:25670526-C/A	*TOP2B*	3:25670526	White	UCEC	NA	0.407	.
3:25679679-T/C	*TOP2B*	3:25679679	Asian	UCEC	NA	0.528	.
3:25683839-C/A	*TOP2B*	3:25683839	Asian	UCEC	NA	0.487	.
3:37035149-G/T	*MLH1*	3:37035149	Asian	UCEC	NA	0.685	.
3:37035149-G/T	*MLH1*	3:37035149	Asian	UCEC	NA	0.903	.
3:37067243-G/A	*MLH1*	3:37067243	White	COAD	rs63750430	0.844	uncertain_significance
3:37067243-G/A	*MLH1*	3:37067243	White	COAD	rs63750430	0.825	uncertain_significance
3:37067243-G/A	*MLH1*	3:37067243	Asian	COAD	rs63750430	0.844	uncertain_significance
3:37067243-G/A	*MLH1*	3:37067243	Asian	COAD	rs63750430	0.825	uncertain_significance
3:37089169-G/C	*MLH1*	3:37089169	Asian	BRCA	NA	0.584	.
3:37089169-G/C	*MLH1*	3:37089169	Asian	BRCA	NA	0.84	.
3:37089169-G/C	*MLH1*	3:37089169	White	BRCA	NA	0.584	.
3:37089169-G/C	*MLH1*	3:37089169	White	BRCA	NA	0.84	.
3:39107333-A/G	*WDR48*	3:39107333	White	STAD	NA	0.95	.
3:39107333-A/G	*WDR48*	3:39107333	Asian	STAD	NA	0.95	.
3:39108317-G/C	*WDR48*	3:39108317	AA/B	KIRC	NA	0.77	.

3:39108317-G/C	*WDR48*	3:39108317	White	KIRC	NA	0.77	.
3:39111231-C/T	*WDR48*	3:39111231	White	COAD	rs576224342	0.373	.
3:39111231-C/T	*WDR48*	3:39111231	AA/B	COAD	rs576224342	0.373	.
3:39125732-C/A	*WDR48*	3:39125732	Asian	UCEC	NA	0.504	.
3:47651784-G/A	*SMARCC1*	3:47651784	White	STAD	rs760989176	0.335	.
3:47651784-G/A	*SMARCC1*	3:47651784	Asian	STAD	rs760989176	0.335	.
3:47704027-C/T	*SMARCC1*	3:47704027	White	UCEC	NA	0.533	.
3:47718187-T/C	*SMARCC1*	3:47718187	Asian	UCEC	rs779079454	0.525	.
3:47752239-A/C	*SMARCC1*	3:47752239	Asian	UCEC	NA	0.48	.
3:47779562-C/T	*SMARCC1*	3:47779562	White	STAD	NA	0.4	.
3:48200506-G/A	*CDC25A*	3:48200506	White	UCEC	rs766101355	0.607	.
3:48200521-C/A	*CDC25A*	3:48200521	AA/B	CESC	NA	0.244	.
3:48200521-C/A	*CDC25A*	3:48200521	White	CESC	NA	0.244	.
3:48224493-G/C	*CDC25A*	3:48224493	White	CESC	NA	0.228	.
3:48224493-G/C	*CDC25A*	3:48224493	AA/B	CESC	NA	0.228	.
3:48224499-G/A	*CDC25A*	3:48224499	White	CESC	NA	0.45	.
3:48224499-G/A	*CDC25A*	3:48224499	AA/B	CESC	NA	0.45	.
3:48226174-C/A	*CDC25A*	3:48226174	White	COAD	NA	0.417	.
3:48493222-G/C	*ATRIP*	3:48493222	AA/B	BRCA	NA	0.177	.
3:48493222-G/C	*ATRIP*	3:48493222	White	BRCA	NA	0.177	.
3:48506335-C/T	*ATRIP*	3:48506335	White	UCEC	rs752926281	0.633	.
3:51979583-C/T	*PARP3*	3:51979583	Asian	STAD	NA	0.154	.
3:51979583-C/T	*PARP3*	3:51979583	White	STAD	NA	0.154	.

3:51979926-C/T	*PARP3*	3:51979926	Asian	UCEC	NA	0.093	.
3:52437675-C/T	*BAP1*	3:52437675	White	UCEC	NA	0.31	.
3:52441201-G/A	*BAP1*	3:52441201	White	UCEC	NA	0.773	.
3:52441201-G/T	*BAP1*	3:52441201	Asian	STAD	NA	0.771	.
3:52441201-G/T	*BAP1*	3:52441201	White	STAD	NA	0.771	.
3:52441235-G/A	*BAP1*	3:52441235	White	LUAD	NA	0.549	.
3:52441235-G/A	*BAP1*	3:52441235	AA/B	LUAD	NA	0.549	.
3:52441246-G/T	*BAP1*	3:52441246	Asian	STAD	NA	0.556	.
3:52441246-G/T	*BAP1*	3:52441246	White	STAD	NA	0.556	.
3:52442072-T/C	*BAP1*	3:52442072	AA/B	BRCA	rs375129361	0.829	pathogenic
3:52442072-T/C	*BAP1*	3:52442072	White	BRCA	rs375129361	0.829	pathogenic
3:52442525-C/A	*BAP1*	3:52442525	White	COAD	NA	0.321	.
3:52442600-G/C	*BAP1*	3:52442600	AA/B	BRCA	NA	0.645	.
3:52442600-G/C	*BAP1*	3:52442600	White	BRCA	NA	0.645	.
3:52443600-T/G	*BAP1*	3:52443600	AA/B	KIRC	NA	0.807	.
3:52443600-T/G	*BAP1*	3:52443600	White	KIRC	NA	0.807	.
3:73114050-A/C	*PPP4R2*	3:73114050	Asian	UCEC	NA	0.342	.
3:73114050-A/C	*PPP4R2*	3:73114050	Asian	UCEC	NA	0.222	.
3:73114220-G/T	*PPP4R2*	3:73114220	White	UCEC	NA	0.177	.
3:73114220-G/T	*PPP4R2*	3:73114220	White	UCEC	NA	0.14	.
3:8977615-T/C	*RAD18*	3:8977615	White	THYM	NA	0.821	.
3:8977615-T/C	*RAD18*	3:8977615	Asian	THYM	NA	0.821	.
3:8977636-T/G	*RAD18*	3:8977636	Asian	COAD	NA	0.119	.

3:8977636-T/G	*RAD18*	3:8977636	White	COAD	NA	0.119	.
3:8977673-C/A	*RAD18*	3:8977673	Asian	UCEC	NA	0.339	.
3:8990233-T/G	*RAD18*	3:8990233	White	STAD	NA	0.639	.
3:8990234-T/G	*RAD18*	3:8990234	Asian	UCEC	NA	0.547	.
3:9000630-A/G	*RAD18*	3:9000630	Asian	STAD	NA	0.221	.
3:9000630-A/G	*RAD18*	3:9000630	White	STAD	NA	0.221	.
3:9000667-G/A	*RAD18*	3:9000667	White	UCEC	rs907328860	0.339	.
3:9793484-A/G	*OGG1*	3:9793484	White	COAD	NA	0.901	.
3:9793484-A/G	*OGG1*	3:9793484	White	COAD	NA	0.963	.
3:9793484-A/G	*OGG1*	3:9793484	AA/B	COAD	NA	0.901	.
3:9793484-A/G	*OGG1*	3:9793484	AA/B	COAD	NA	0.963	.
4:1343439-C/T	*UVSSA*	4:1343439	White	COAD	NA	0.582	.
4:1343439-C/T	*UVSSA*	4:1343439	White	UCEC	NA	0.582	.
4:1343439-C/T	*UVSSA*	4:1343439	AA/B	COAD	NA	0.582	.
4:1343542-G/T	*UVSSA*	4:1343542	Asian	ESCA	NA	0.252	.
4:1343542-G/T	*UVSSA*	4:1343542	White	ESCA	NA	0.252	.
4:1345537-C/G	*UVSSA*	4:1345537	White	STAD	NA	0.181	.
4:1345537-C/G	*UVSSA*	4:1345537	Asian	STAD	NA	0.181	.
4:1348915-G/A	*UVSSA*	4:1348915	Asian	STAD	rs563763967	0.253	.
4:1348915-G/A	*UVSSA*	4:1348915	White	STAD	rs563763967	0.253	.
4:1369233-C/G	*UVSSA*	4:1369233	White	ESCA	NA	0.479	.
4:1369233-C/G	*UVSSA*	4:1369233	Asian	ESCA	NA	0.479	.
4:144445525-G/A	*SMARCA5*	4:144445525	White	UCEC	NA	0.723	.

4:144456093-G/T	*SMARCA5*	4:144456093	White	COAD	NA	0.734	.
4:144456093-G/T	*SMARCA5*	4:144456093	AA/B	COAD	NA	0.734	.
4:144459999-C/T	*SMARCA5*	4:144459999	Asian	STAD	rs778415632	0.75	.
4:144459999-C/T	*SMARCA5*	4:144459999	White	STAD	rs778415632	0.75	.
4:144464730-G/T	*SMARCA5*	4:144464730	White	UCEC	NA	0.62	.
4:144465105-G/T	*SMARCA5*	4:144465105	White	UCEC	NA	0.908	.
4:144469166-G/A	*SMARCA5*	4:144469166	White	COAD	NA	0.813	.
4:154271274-C/G	*MND1*	4:154271274	AA/B	BRCA	NA	0.178	.
4:154271274-C/G	*MND1*	4:154271274	White	BRCA	NA	0.178	.
4:154318378-G/A	*MND1*	4:154318378	White	UCEC	rs751477709	0.681	.
4:154335936-G/T	*MND1*	4:154335936	Asian	UCEC	NA	0.433	.
4:154335996-A/G	*MND1*	4:154335996	Asian	STAD	NA	0.651	.
4:154335996-A/G	*MND1*	4:154335996	White	STAD	NA	0.651	.
4:155458475-T/C	*PLRG1*	4:155458475	White	STAD	NA	0.942	.
4:155458475-T/C	*PLRG1*	4:155458475	Asian	STAD	NA	0.942	.
4:155458479-C/A	*PLRG1*	4:155458479	White	UCEC	NA	0.508	.
4:155461784-C/T	*PLRG1*	4:155461784	White	BRCA	rs1280751067	0.367	.
4:155461784-C/T	*PLRG1*	4:155461784	Asian	BRCA	rs1280751067	0.367	.
4:155461951-G/T	*PLRG1*	4:155461951	Asian	UCEC	NA	0.488	.
4:178231192-G/A	*NEIL3*	4:178231192	White	STAD	rs1263191299	0.249	.
4:178231192-G/A	*NEIL3*	4:178231192	Asian	STAD	rs1263191299	0.249	.
4:178262667-A/C	*NEIL3*	4:178262667	Asian	UCEC	NA	0.249	.
4:178262720-C/T	*NEIL3*	4:178262720	AA/B	COAD	rs762418261	0.432	.

4:178262720-C/T	*NEIL3*	4:178262720	White	COAD	rs762418261	0.432	.
4:178262744-A/T	*NEIL3*	4:178262744	White	STAD	NA	0.266	.
4:178262744-A/T	*NEIL3*	4:178262744	Asian	STAD	NA	0.266	.
4:178274525-A/G	*NEIL3*	4:178274525	Asian	STAD	NA	0.127	.
4:178274525-A/G	*NEIL3*	4:178274525	White	STAD	NA	0.127	.
4:178283483-G/A	*NEIL3*	4:178283483	White	STAD	rs149552701	0.467	.
4:2073999-C/T	*POLN*	4:2073999	Asian	STAD	rs530335133	0.776	.
4:2073999-C/T	*POLN*	4:2073999	White	STAD	rs530335133	0.776	.
4:2077220-A/G	*POLN*	4:2077220	White	STAD	NA	0.936	.
4:2077220-A/G	*POLN*	4:2077220	White	STAD	NA	0.596	.
4:2077220-A/G	*POLN*	4:2077220	Asian	STAD	NA	0.936	.
4:2077220-A/G	*POLN*	4:2077220	Asian	STAD	NA	0.596	.
4:2097609-C/A	*POLN*	4:2097609	White	UCEC	NA	0.327	.
4:2097609-C/A	*POLN*	4:2097609	White	UCEC	NA	0.787	.
4:2158559-T/A	*POLN*	4:2158559	White	UCEC	NA	0.861	.
4:2172450-G/A	*POLN*	4:2172450	White	STAD	NA	0.126	.
4:39304373-C/T	*RFC1*	4:39304373	White	UCEC	rs199688793	0.402	.
4:39306472-T/A	*RFC1*	4:39306472	White	ESCA	NA	0.449	.
4:39306472-T/A	*RFC1*	4:39306472	Asian	ESCA	NA	0.449	.
4:39310299-C/A	*RFC1*	4:39310299	AA/B	LIHC	NA	0.69	.
4:84342795-A/C	*HELQ*	4:84342795	White	UCEC	rs759942417	0.784	.
4:84342852-G/A	*HELQ*	4:84342852	White	COAD	NA	0.717	.
4:84348790-G/T	*HELQ*	4:84348790	White	COAD	NA	0.565	.

4:84350853-A/C	*HELQ*	4:84350853	White	COAD	NA	0.399	.
4:84368010-A/T	*HELQ*	4:84368010	White	BRCA	NA	0.742	.
4:84368010-A/T	*HELQ*	4:84368010	Asian	BRCA	NA	0.742	.
4:84368140-C/T	*HELQ*	4:84368140	AA/B	SARC	NA	0.588	.
4:84368140-C/T	*HELQ*	4:84368140	White	SARC	NA	0.588	.
4:84369965-G/T	*HELQ*	4:84369965	Asian	UCEC	NA	0.358	.
4:84370084-G/A	*HELQ*	4:84370084	Asian	UCEC	rs764392725	0.274	.
4:84370084-G/T	*HELQ*	4:84370084	White	COAD	NA	0.258	.
4:84383770-C/T	*FAM175A*	4:84383770	White	COAD	rs201627097	0.199	.
4:84383770-C/T	*FAM175A*	4:84383770	AA/B	COAD	rs201627097	0.199	.
4:84383771-G/A	*FAM175A*	4:84383771	Asian	STAD	rs1345611154	0.234	.
4:84383771-G/A	*FAM175A*	4:84383771	White	STAD	rs1345611154	0.234	.
4:95171918-C/T	*SMARCAD1*	4:95171918	White	UCEC	NA	0.258	.
4:95174040-A/C	*SMARCAD1*	4:95174040	Asian	COAD	NA	0.709	.
4:95174040-A/C	*SMARCAD1*	4:95174040	White	COAD	NA	0.709	.
4:95174133-G/A	*SMARCAD1*	4:95174133	White	LUAD	NA	0.798	.
4:95174133-G/A	*SMARCAD1*	4:95174133	AA/B	LUAD	NA	0.798	.
4:95185970-T/A	*SMARCAD1*	4:95185970	White	STAD	NA	0.795	.
4:95185970-T/A	*SMARCAD1*	4:95185970	Asian	STAD	NA	0.795	.
4:95199614-A/G	*SMARCAD1*	4:95199614	AA/B	KIRC	NA	0.814	.
4:95199614-A/G	*SMARCAD1*	4:95199614	White	KIRC	NA	0.814	.
4:95200148-C/T	*SMARCAD1*	4:95200148	White	UCEC	NA	0.799	.
5:131893034-G/T	*RAD50*	5:131893034	White	COAD	NA	0.287	.

5:131895051-G/A	*RAD50*	5:131895051	White	UCEC	rs370769989	0.443	uncertain_significance
5:131915135-G/T	*RAD50*	5:131915135	White	LUAD	NA	0.589	.
5:131915135-G/T	*RAD50*	5:131915135	AA/B	LUAD	NA	0.589	.
5:131930676-G/A	*RAD50*	5:131930676	White	UCEC	NA	0.131	.
5:131931471-C/T	*RAD50*	5:131931471	Asian	STAD	rs753002904	0.159	.
5:131931471-C/T	*RAD50*	5:131931471	White	STAD	rs753002904	0.159	.
5:131951732-G/T	*RAD50*	5:131951732	White	UCEC	NA	0.301	.
5:137621728-A/G	*CDC25C*	5:137621728	AA/B	LUAD	NA	0.665	.
5:137621728-A/G	*CDC25C*	5:137621728	AA/B	LUAD	NA	0.565	.
5:137621728-A/G	*CDC25C*	5:137621728	White	LUAD	NA	0.665	.
5:137621728-A/G	*CDC25C*	5:137621728	White	LUAD	NA	0.565	.
5:137622189-C/T	*CDC25C*	5:137622189	White	COAD	rs200759602	0.08	.
5:137802793-G/A	*EGR1*	5:137802793	AA/B	LUAD	NA	0.207	.
5:137802793-G/A	*EGR1*	5:137802793	White	LUAD	NA	0.207	.
5:176385134-C/G	*UIMC1*	5:176385134	AA/B	BRCA	NA	0.072	.
5:176385134-C/G	*UIMC1*	5:176385134	White	BRCA	NA	0.072	.
5:176409580-C/T	*UIMC1*	5:176409580	Asian	UCEC	rs548830338	0.035	.
5:34908989-G/A	*RAD1*	5:34908989	Asian	UCEC	5:34908989_G/A	0.713	.
5:34911836-G/A	*RAD1*	5:34911836	AA/B	LUSC	NA	0.543	.
5:34911836-G/A	*RAD1*	5:34911836	White	LUSC	NA	0.543	.
5:60195503-C/G	*ERCC8*	5:60195503	White	LUSC	NA	0.487	.
5:60195503-C/G	*ERCC8*	5:60195503	AA/B	LUSC	NA	0.487	.

5:68531245-G/T	*CDK7*	5:68531245	Asian	UCEC	NA	0.601	.
5:68670485-G/A	*RAD17*	5:68670485	AA/B	BRCA	NA	0.127	.
5:68670485-G/A	*RAD17*	5:68670485	White	BRCA	NA	0.127	.
5:68677857-G/T	*RAD17*	5:68677857	Asian	UCEC	NA	0.116	.
5:68680719-G/A	*RAD17*	5:68680719	White	CESC	NA	0.572	.
5:68680719-G/A	*RAD17*	5:68680719	AA/B	CESC	NA	0.572	.
5:68681898-C/A	*RAD17*	5:68681898	White	STAD	NA	0.093	.
5:68684910-G/T	*RAD17*	5:68684910	AA/B	COAD	NA	0.641	.
5:68684910-G/T	*RAD17*	5:68684910	White	COAD	NA	0.641	.
5:68684956-G/T	*RAD17*	5:68684956	Asian	UCEC	NA	0.302	.
5:68695918-T/G	*RAD17*	5:68695918	AA/B	LUAD	NA	0.441	.
5:68695918-T/G	*RAD17*	5:68695918	White	LUAD	NA	0.441	.
5:74869615-G/A	*POLK*	5:74869615	White	STAD	NA	0.753	.
5:74877213-G/A	*POLK*	5:74877213	White	UCEC	rs142203892	0.528	.
5:74877231-C/T	*POLK*	5:74877231	White	COAD	rs200548318	0.865	.
5:74879241-A/C	*POLK*	5:74879241	Asian	UCEC	NA	0.887	.
5:74880598-G/A	*POLK*	5:74880598	Asian	STAD	NA	0.967	.
5:74880598-G/A	*POLK*	5:74880598	White	STAD	NA	0.967	.
5:74882873-A/T	*POLK*	5:74882873	White	COAD	NA	0.616	.
5:74882873-A/T	*POLK*	5:74882873	AA/B	COAD	NA	0.616	.
5:79966006-C/T	*MSH3*	5:79966006	Asian	STAD	rs748207601	0.377	.
5:79966006-C/T	*MSH3*	5:79966006	White	STAD	rs748207601	0.377	.
5:79966082-G/A	*MSH3*	5:79966082	White	COAD	NA	0.639	.

5:79966082-G/A	*MSH3*	5:79966082	Asian	COAD	NA	0.639	.
5:80021297-G/A	*MSH3*	5:80021297	White	UCEC	rs963234468	0.77	.
5:80063766-G/T	*MSH3*	5:80063766	White	COAD	NA	0.608	.
5:80064756-T/A	*MSH3*	5:80064756	AA/B	BRCA	NA	0.449	.
5:80064756-T/A	*MSH3*	5:80064756	White	BRCA	NA	0.449	.
5:80109434-G/T	*MSH3*	5:80109434	White	LUAD	NA	0.988	.
5:80109434-G/T	*MSH3*	5:80109434	AA/B	LUAD	NA	0.988	.
5:80109451-A/C	*MSH3*	5:80109451	White	STAD	rs763041578	0.948	.
5:82406919-G/T	*XRCC4*	5:82406919	White	UCEC	NA	0.27	.
5:82491728-T/C	*XRCC4*	5:82491728	White	STAD	5:82491728_T/C	0.188	.
5:86700748-G/A	*CCNH*	5:86700748	White	UCEC	rs145378845	0.702	.
5:86703989-A/G	*CCNH*	5:86703989	Asian	UCEC	rs758248932	0.448	.
5:86705156-G/A	*CCNH*	5:86705156	White	UCEC	rs750420341	0.608	.
6:100957932-C/T	*ASCC3*	6:100957932	Asian	STAD	rs201205349	0.557	.
6:100957932-C/T	*ASCC3*	6:100957932	White	STAD	rs201205349	0.557	.
6:101073115-C/T	*ASCC3*	6:101073115	White	BRCA	NA	0.594	.
6:101073115-C/T	*ASCC3*	6:101073115	AA/B	BRCA	NA	0.594	.
6:101073157-G/A	*ASCC3*	6:101073157	AA/B	BRCA	rs757716813	0.797	.
6:101073157-G/A	*ASCC3*	6:101073157	White	BRCA	rs757716813	0.797	.
6:101075555-C/T	*ASCC3*	6:101075555	Asian	UCEC	rs148742449	0.941	.
6:101075816-A/C	*ASCC3*	6:101075816	Asian	UCEC	NA	0.572	.
6:101076966-C/A	*ASCC3*	6:101076966	White	STAD	NA	0.272	.
6:101076966-C/A	*ASCC3*	6:101076966	Asian	STAD	NA	0.272	.

6:101086686-G/C	*ASCC3*	6:101086686	White	STAD	NA	0.356	.
6:101086686-G/C	*ASCC3*	6:101086686	Asian	STAD	NA	0.356	.
6:101090570-A/C	*ASCC3*	6:101090570	White	COAD	NA	0.602	.
6:101090570-A/C	*ASCC3*	6:101090570	AA/B	COAD	NA	0.602	.
6:101091945-T/C	*ASCC3*	6:101091945	White	STAD	NA	0.328	.
6:101095148-T/G	*ASCC3*	6:101095148	Asian	UCEC	NA	0.221	.
6:101166000-C/T	*ASCC3*	6:101166000	White	STAD	rs142555952	0.893	.
6:101214491-C/T	*ASCC3*	6:101214491	Asian	STAD	NA	0.524	.
6:101214491-C/T	*ASCC3*	6:101214491	White	STAD	NA	0.524	.
6:111631223-C/T	*REV3L*	6:111631223	AA/B	COAD	rs749887147	0.419	.
6:111631223-C/T	*REV3L*	6:111631223	White	COAD	rs749887147	0.419	.
6:111632452-C/T	*REV3L*	6:111632452	White	STAD	rs374701728	0.359	.
6:111632452-C/T	*REV3L*	6:111632452	Asian	STAD	rs374701728	0.359	.
6:111636522-G/A	*REV3L*	6:111636522	Asian	STAD	NA	0.273	.
6:111636522-G/A	*REV3L*	6:111636522	White	STAD	NA	0.273	.
6:111636577-C/T	*REV3L*	6:111636577	White	UCEC	NA	0.427	.
6:111650820-G/A	*REV3L*	6:111650820	Asian	STAD	rs376298082	0.382	.
6:111650820-G/A	*REV3L*	6:111650820	White	STAD	rs376298082	0.382	.
6:111650832-C/T	*REV3L*	6:111650832	White	UCEC	rs141338934	0.253	.
6:111650877-A/G	*REV3L*	6:111650877	Asian	UCEC	NA	0.48	.
6:111680142-C/A	*REV3L*	6:111680142	White	STAD	NA	0.634	.
6:111688861-C/A	*REV3L*	6:111688861	AA/B	KIRC	NA	0.097	.
6:111688861-C/A	*REV3L*	6:111688861	White	KIRC	NA	0.097	.

6:111694807-C/T	*REV3L*	6:111694807	White	STAD	rs776259034	0.262	.
6:111696389-T/C	*REV3L*	6:111696389	White	BRCA	NA	0.139	.
6:111696389-T/C	*REV3L*	6:111696389	Asian	BRCA	NA	0.139	.
6:111696655-G/T	*REV3L*	6:111696655	White	COAD	NA	0.187	.
6:111696655-G/T	*REV3L*	6:111696655	Asian	COAD	NA	0.187	.
6:111701366-C/T	*REV3L*	6:111701366	Asian	STAD	NA	0.151	.
6:111701366-C/T	*REV3L*	6:111701366	White	STAD	NA	0.151	.
6:111726690-C/T	*REV3L*	6:111726690	White	UCEC	NA	0.309	.
6:146214462-G/T	*SHPRH*	6:146214462	White	UCEC	NA	0.823	.
6:146240548-C/G	*SHPRH*	6:146240548	White	STAD	NA	0.626	.
6:146240548-C/G	*SHPRH*	6:146240548	Asian	STAD	NA	0.626	.
6:146242419-C/G	*SHPRH*	6:146242419	White	BRCA	NA	0.501	.
6:146242419-C/G	*SHPRH*	6:146242419	AA/B	BRCA	NA	0.501	.
6:146256096-C/A	*SHPRH*	6:146256096	White	LUAD	NA	0.868	.
6:146256096-C/A	*SHPRH*	6:146256096	AA/B	LUAD	NA	0.868	.
6:146256106-C/A	*SHPRH*	6:146256106	Asian	STAD	NA	0.733	.
6:146256106-C/A	*SHPRH*	6:146256106	White	STAD	NA	0.733	.
6:146256140-T/A	*SHPRH*	6:146256140	White	LUAD	NA	0.785	.
6:146256140-T/A	*SHPRH*	6:146256140	AA/B	LUAD	NA	0.785	.
6:146261936-G/T	*SHPRH*	6:146261936	White	SARC	NA	0.941	.
6:146261936-G/T	*SHPRH*	6:146261936	AA/B	SARC	NA	0.941	.
6:152163841-T/C	*ESR1*	6:152163841	White	UCEC	NA	0.981	.
6:152201865-G/A	*ESR1*	6:152201865	Asian	STAD	NA	0.963	.

6:152201865-G/A	*ESR1*	6:152201865	Asian	STAD	NA	0.772	.
6:152201865-G/A	*ESR1*	6:152201865	White	STAD	NA	0.963	.
6:152201865-G/A	*ESR1*	6:152201865	White	STAD	NA	0.772	.
6:24651150-A/C	*TDP2*	6:24651150	Asian	UCEC	NA	0.7	.
6:30670606-C/T	*MDC1*	6:30670606	White	UCEC	rs1371318631	0.545	.
6:30671314-C/A	*MDC1*	6:30671314	AA/B	LUAD	NA	0.087	.
6:30671314-C/A	*MDC1*	6:30671314	White	LUAD	NA	0.087	.
6:30671903-G/T	*MDC1*	6:30671903	Asian	UCEC	rs758783364	0.082	.
6:30672029-C/A	*MDC1*	6:30672029	Asian	STAD	NA	0.071	.
6:30672029-C/A	*MDC1*	6:30672029	White	STAD	NA	0.071	.
6:30679962-G/T	*MDC1*	6:30679962	AA/B	KIRP	NA	0.099	.
6:30679962-G/T	*MDC1*	6:30679962	White	KIRP	NA	0.099	.
6:30680815-C/A	*MDC1*	6:30680815	Asian	UCEC	NA	0.139	.
6:30880141-C/T	*GTF2H4*	6:30880141	White	CESC	NA	0.427	.
6:30881608-C/A	*GTF2H4*	6:30881608	White	STAD	NA	0.156	.
6:30881608-C/A	*GTF2H4*	6:30881608	Asian	STAD	NA	0.156	.
6:35423809-G/T	*FANCE*	6:35423809	AA/B	LUAD	NA	0.06	.
6:35423809-G/T	*FANCE*	6:35423809	White	LUAD	NA	0.06	.
6:35423873-C/T	*FANCE*	6:35423873	AA/B	COAD	rs763151358	0.15	.
6:35425693-G/T	*FANCE*	6:35425693	Asian	THCA	NA	0.154	.
6:35425693-G/T	*FANCE*	6:35425693	White	THCA	NA	0.154	.
6:35434088-C/A	*FANCE*	6:35434088	White	UCEC	NA	0.214	.
6:36334674-G/C	*ETV7*	6:36334674	AA/B	BRCA	NA	0.191	.

6:36334674-G/C	*ETV7*	6:36334674	White	BRCA	NA	0.191	.
6:36334709-C/T	*ETV7*	6:36334709	White	UCEC	rs768642185	0.784	.
6:36336846-A/G	*ETV7*	6:36336846	White	UCEC	NA	0.474	.
6:36339127-G/A	*ETV7*	6:36339127	Asian	UCEC	NA	0.215	.
6:36343764-C/G	*ETV7*	6:36343764	AA/B	LUAD	NA	0.286	.
6:36343764-C/G	*ETV7*	6:36343764	White	LUAD	NA	0.286	.
6:37339288-G/T	*RNF8*	6:37339288	AA/B	LGG	NA	0.064	.
6:37339288-G/T	*RNF8*	6:37339288	AA/B	LGG	NA	0.201	.
6:37339288-G/T	*RNF8*	6:37339288	White	LGG	NA	0.064	.
6:37339288-G/T	*RNF8*	6:37339288	White	LGG	NA	0.201	.
6:37344732-G/A	*RNF8*	6:37344732	White	COAD	rs769385134	0.32	.
6:37344732-G/A	*RNF8*	6:37344732	White	COAD	rs769385134	0.348	.
6:37349065-A/C	*RNF8*	6:37349065	White	COAD	NA	0.505	.
6:43550169-A/T	*POLH*	6:43550169	AA/B	LUAD	NA	0.839	.
6:43550169-A/T	*POLH*	6:43550169	White	LUAD	NA	0.839	.
6:43578327-C/T	*POLH*	6:43578327	White	UCEC	rs147564503	0.398	.
6:43578345-C/T	*POLH*	6:43578345	White	COAD	NA	0.255	.
6:43578345-C/T	*POLH*	6:43578345	AA/B	COAD	NA	0.255	.
6:44358050-T/C	*CDC5L*	6:44358050	Asian	UCEC	NA	0.888	.
6:44360556-A/C	*CDC5L*	6:44360556	White	STAD	NA	0.708	.
6:44364173-G/A	*CDC5L*	6:44364173	White	UCEC	NA	0.299	.
6:44397637-A/T	*CDC5L*	6:44397637	Asian	UCEC	NA	0.205	.
6:52132745-G/A	*MCM3*	6:52132745	Asian	STAD	rs143308289	0.156	.

6:52132745-G/A	*MCM3*	6:52132745	White	STAD	rs143308289	0.156	.
6:74468735-G/T	*CD109*	6:74468735	White	LUAD	NA	0.275	.
6:74468735-G/T	*CD109*	6:74468735	AA/B	LUAD	NA	0.275	.
6:74473391-C/G	*CD109*	6:74473391	Asian	BRCA	NA	0.175	.
6:74473391-C/G	*CD109*	6:74473391	White	BRCA	NA	0.175	.
6:74475660-T/C	*CD109*	6:74475660	Asian	UCEC	NA	0.172	.
6:74492433-G/A	*CD109*	6:74492433	AA/B	COAD	NA	0.283	.
6:74492433-G/A	*CD109*	6:74492433	White	COAD	NA	0.283	.
6:74519785-C/T	*CD109*	6:74519785	White	UCEC	NA	0.321	.
6:74519866-G/A	*CD109*	6:74519866	Asian	UCEC	NA	0.363	.
6:74520832-A/C	*CD109*	6:74520832	White	UCEC	NA	0.054	.
6:80717571-C/T	*TTK*	6:80717571	AA/B	LUAD	NA	0.656	.
6:80717571-C/T	*TTK*	6:80717571	White	LUAD	NA	0.656	.
6:80745032-G/C	*TTK*	6:80745032	AA/B	BRCA	NA	0.891	.
6:80745032-G/C	*TTK*	6:80745032	White	BRCA	NA	0.891	.
6:80746264-G/A	*TTK*	6:80746264	Asian	BRCA	NA	0.894	.
6:80746264-G/A	*TTK*	6:80746264	White	BRCA	NA	0.894	.
6:80749470-G/T	*TTK*	6:80749470	AA/B	LUSC	NA	0.698	.
6:80749470-G/T	*TTK*	6:80749470	White	LUSC	NA	0.698	.
6:80749510-A/C	*TTK*	6:80749510	Asian	UCEC	NA	0.676	.
6:80749527-G/T	*TTK*	6:80749527	White	UCEC	NA	0.597	.
6:97599743-A/T	*MMS22L*	6:97599743	Asian	ESCA	NA	0.323	.
6:97599743-A/T	*MMS22L*	6:97599743	White	ESCA	NA	0.323	.

6:97613184-C/G	*MMS22L*	6:97613184	Asian	ESCA	NA	0.09	.
6:97613184-C/G	*MMS22L*	6:97613184	White	ESCA	NA	0.09	.
6:97626411-G/T	*MMS22L*	6:97626411	White	UCEC	NA	0.261	.
6:97627350-T/C	*MMS22L*	6:97627350	AA/B	BRCA	rs1417953473	0.132	.
6:97627350-T/C	*MMS22L*	6:97627350	White	BRCA	rs1417953473	0.132	.
6:97634457-G/T	*MMS22L*	6:97634457	Asian	UCEC	NA	0.232	.
6:97634485-C/A	*MMS22L*	6:97634485	Asian	STAD	NA	0.124	.
6:97634485-C/A	*MMS22L*	6:97634485	White	STAD	NA	0.124	.
7:103777292-C/T	*ORC5*	7:103777292	White	UCEC	rs140616327	0.163	.
7:103801549-C/A	*ORC5*	7:103801549	AA/B	LUAD	NA	0.352	.
7:103801549-C/A	*ORC5*	7:103801549	White	LUAD	NA	0.352	.
7:103838219-T/C	*ORC5*	7:103838219	White	LUSC	NA	0.372	.
7:103838219-T/C	*ORC5*	7:103838219	AA/B	LUSC	NA	0.372	.
7:103848285-C/T	*ORC5*	7:103848285	White	UCEC	NA	0.466	.
7:105903857-G/T	*NAMPT*	7:105903857	White	LGG	NA	0.837	.
7:105903857-G/T	*NAMPT*	7:105903857	AA/B	LGG	NA	0.837	.
7:105903963-C/A	*NAMPT*	7:105903963	White	LUAD	NA	0.865	.
7:105903963-C/A	*NAMPT*	7:105903963	AA/B	LUAD	NA	0.865	.
7:105917478-A/G	*NAMPT*	7:105917478	White	COAD	NA	0.543	.
7:105917501-C/A	*NAMPT*	7:105917501	AA/B	BRCA	NA	0.906	.
7:105917501-C/A	*NAMPT*	7:105917501	White	BRCA	NA	0.906	.
7:105917576-T/C	*NAMPT*	7:105917576	White	LUAD	NA	0.613	.
7:105917576-T/C	*NAMPT*	7:105917576	AA/B	LUAD	NA	0.613	.

7:150751117-G/T	*CDK5*	7:150751117	Asian	UCEC	NA	0.316	.
7:150753708-A/C	*CDK5*	7:150753708	White	COAD	NA	0.367	.
7:154774988-C/A	*PAXIP1*	7:154774988	White	STAD	NA	0.424	.
7:154774988-C/A	*PAXIP1*	7:154774988	Asian	STAD	NA	0.424	.
7:154790377-C/A	*PAXIP1*	7:154790377	White	LUAD	NA	0.47	.
7:154790377-C/A	*PAXIP1*	7:154790377	AA/B	LUAD	NA	0.47	.
7:40172675-C/T	*MPLKIP*	7:40172675	Asian	STAD	NA	0.603	.
7:40172675-C/T	*MPLKIP*	7:40172675	White	STAD	NA	0.603	.
7:44155380-C/T	*POLD2*	7:44155380	White	STAD	NA	0.385	.
7:44155380-C/T	*POLD2*	7:44155380	Asian	STAD	NA	0.385	.
7:44155855-T/C	*POLD2*	7:44155855	White	STAD	NA	0.66	.
7:44155855-T/C	*POLD2*	7:44155855	Asian	STAD	NA	0.66	.
7:55221710-C/T	*EGFR*	7:55221710	AA/B	LGG	NA	0.581	.
7:55221710-C/T	*EGFR*	7:55221710	White	LGG	NA	0.581	.
7:55221723-A/G	*EGFR*	7:55221723	AA/B	LGG	NA	0.631	.
7:55221723-A/G	*EGFR*	7:55221723	White	LGG	NA	0.631	.
7:55221822-C/T	*EGFR*	7:55221822	AA/B	BRCA	NA	0.647	.
7:55221822-C/T	*EGFR*	7:55221822	AA/B	LGG	NA	0.647	.
7:55221822-C/T	*EGFR*	7:55221822	White	LGG	NA	0.647	.
7:55221822-C/T	*EGFR*	7:55221822	White	BRCA	NA	0.647	.
7:55223531-G/A	*EGFR*	7:55223531	AA/B	LUAD	7:55223531_G/A	0.492	.
7:55223531-G/A	*EGFR*	7:55223531	White	LUAD	7:55223531_G/A	0.492	.
7:55225430-G/A	*EGFR*	7:55225430	White	UCEC	NA	0.807	.

7:55229210-G/A	*EGFR*	7:55229210	White	UCEC	NA	0.885	.
7:55240750-G/A	*EGFR*	7:55240750	White	LGG	NA	0.489	.
7:55240750-G/A	*EGFR*	7:55240750	AA/B	LGG	NA	0.489	.
7:55241668-A/C	*EGFR*	7:55241668	White	UCEC	NA	0.427	.
7:55242452-C/T	*EGFR*	7:55242452	White	SARC	NA	0.729	.
7:55242452-C/T	*EGFR*	7:55242452	AA/B	SARC	NA	0.729	.
7:55242457-G/A	*EGFR*	7:55242457	White	UCEC	rs759256622	0.91	.
7:55249069-C/G	*EGFR*	7:55249069	White	CESC	NA	0.738	.
7:55249069-C/G	*EGFR*	7:55249069	AA/B	CESC	NA	0.738	.
7:55249071-C/T	*EGFR*	7:55249071	White	LUAD	rs121434569	0.529	likely_pathogenic,drug_response,protective
7:55249071-C/T	*EGFR*	7:55249071	AA/B	LUAD	rs121434569	0.529	likely_pathogenic,drug_response,protective
7:55249134-C/G	*EGFR*	7:55249134	AA/B	CESC	rs1378775962	0.531	.
7:55249134-C/G	*EGFR*	7:55249134	White	CESC	rs1378775962	0.531	.
7:55259482-C/A	*EGFR*	7:55259482	White	BRCA	NA	0.525	.
7:55259482-C/A	*EGFR*	7:55259482	Asian	BRCA	NA	0.525	.
7:55266491-A/T	*EGFR*	7:55266491	White	BRCA	NA	0.761	.
7:55266491-A/T	*EGFR*	7:55266491	AA/B	BRCA	NA	0.761	.
7:55273092-C/T	*EGFR*	7:55273092	White	ESCA	rs1473630143	0.48	.
7:55273092-C/T	*EGFR*	7:55273092	Asian	ESCA	rs1473630143	0.48	.

7:6018254-C/A	*PMS2*	7:6018254	White	COAD	NA	0.993	.
7:6018254-C/A	*PMS2*	7:6018254	AA/B	COAD	NA	0.993	.
7:6026456-T/G	*PMS2*	7:6026456	White	STAD	NA	0.086	.
7:6026456-T/G	*PMS2*	7:6026456	Asian	STAD	NA	0.086	.
7:6026525-G/T	*PMS2*	7:6026525	Asian	UCEC	NA	0.2	.
7:6035205-T/C	*PMS2*	7:6035205	Asian	UCEC	NA	0.862	.
7:73661080-C/T	*RFC2*	7:73661080	Asian	UCEC	NA	0.452	.
7:7678715-C/T	*RPA3*	7:7678715	AA/B	COAD	rs1279111309	0.596	.
7:7678715-C/T	*RPA3*	7:7678715	White	COAD	rs1279111309	0.596	.
8:117866665-T/C	*RAD21*	8:117866665	White	COAD	NA	0.924	.
8:117875449-C/T	*RAD21*	8:117875449	White	COAD	rs773855925	0.683	.
8:117875482-C/T	*RAD21*	8:117875482	Asian	UCEC	NA	0.737	.
8:126114657-A/C	*NSMCE2*	8:126114657	White	COAD	NA	0.122	.
8:126194403-T/G	*NSMCE2*	8:126194403	White	STAD	NA	0.166	.
8:145737356-C/G	*RECQL4*	8:145737356	Asian	BRCA	NA	NA	.
8:145737356-C/G	*RECQL4*	8:145737356	White	BRCA	NA	NA	.
8:145738257-C/T	*RECQL4*	8:145738257	AA/B	BRCA	rs374225917	NA	.
8:145738257-C/T	*RECQL4*	8:145738257	White	STAD	rs374225917	NA	.
8:145738257-C/T	*RECQL4*	8:145738257	Asian	STAD	rs374225917	NA	.
8:145738257-C/T	*RECQL4*	8:145738257	White	BRCA	rs374225917	NA	.
8:145739866-G/A	*RECQL4*	8:145739866	AA/B	CESC	rs755425093	NA	.
8:145739866-G/A	*RECQL4*	8:145739866	White	CESC	rs755425093	NA	.
8:145740393-G/A	*RECQL4*	8:145740393	White	COAD	rs762113412	NA	.

8:145740393-G/A	*RECQL4*	8:145740393	AA/B	COAD	rs762113412	NA	.
8:145740623-G/C	*RECQL4*	8:145740623	White	BRCA	NA	NA	.
8:145740623-G/C	*RECQL4*	8:145740623	AA/B	BRCA	NA	NA	.
8:18079582-G/T	*NAT1*	8:18079582	Asian	UCEC	rs772568722	0.384	.
8:18079991-G/T	*NAT1*	8:18079991	White	UCEC	NA	0.243	.
8:21965115-G/T	*NUDT18*	8:21965115	Asian	STAD	NA	0.134	.
8:21965115-G/T	*NUDT18*	8:21965115	White	STAD	NA	0.134	.
8:30922579-G/T	*WRN*	8:30922579	White	UCEC	NA	0.393	.
8:30954286-G/T	*WRN*	8:30954286	AA/B	LUAD	NA	0.276	.
8:30954286-G/T	*WRN*	8:30954286	White	LUAD	NA	0.276	.
8:30969237-G/A	*WRN*	8:30969237	White	UCEC	rs777084325	0.424	.
8:30969269-A/C	*WRN*	8:30969269	White	UCEC	NA	0.174	.
8:31014945-C/T	*WRN*	8:31014945	White	STAD	rs146861737	0.209	.
8:31014945-C/T	*WRN*	8:31014945	Asian	STAD	rs146861737	0.209	.
8:39775433-G/T	*IDO1*	8:39775433	Asian	UCEC	NA	0.364	.
8:39782262-T/G	*IDO1*	8:39782262	Asian	UCEC	NA	0.427	.
8:39785379-G/C	*IDO1*	8:39785379	White	ESCA	NA	0.524	.
8:39785379-G/C	*IDO1*	8:39785379	Asian	ESCA	NA	0.524	.
8:42206594-C/T	*POLB*	8:42206594	Asian	STAD	rs201913498	0.566	.
8:42206594-C/T	*POLB*	8:42206594	Asian	STAD	rs201913498	0.285	.
8:42206594-C/T	*POLB*	8:42206594	White	STAD	rs201913498	0.566	.
8:42206594-C/T	*POLB*	8:42206594	White	STAD	rs201913498	0.285	.
8:42214904-A/G	*POLB*	8:42214904	AA/B	LUAD	NA	0.389	.

8:42214904-A/G	*POLB*	8:42214904	AA/B	LUAD	NA	0.745	.
8:42214904-A/G	*POLB*	8:42214904	White	LUAD	NA	0.389	.
8:42214904-A/G	*POLB*	8:42214904	White	LUAD	NA	0.745	.
8:42226841-G/T	*POLB*	8:42226841	Asian	UCEC	NA	0.591	.
8:42226841-G/T	*POLB*	8:42226841	Asian	UCEC	NA	0.717	.
8:54891623-A/G	*TCEA1*	8:54891623	AA/B	COAD	NA	0.965	.
8:54891623-A/G	*TCEA1*	8:54891623	White	COAD	NA	0.965	.
8:75737654-G/T	*PI15*	8:75737654	AA/B	LUAD	NA	0.39	.
8:75737654-G/T	*PI15*	8:75737654	White	LUAD	NA	0.39	.
8:75737735-C/T	*PI15*	8:75737735	Asian	STAD	rs747132125	0.445	.
8:75737735-C/T	*PI15*	8:75737735	White	STAD	rs747132125	0.445	.
8:90958385-A/C	*NBN*	8:90958385	White	UCEC	NA	0.716	.
8:90967568-G/T	*NBN*	8:90967568	White	UCEC	NA	0.228	.
8:90976667-T/C	*NBN*	8:90976667	White	COAD	NA	0.429	.
8:90983450-C/A	*NBN*	8:90983450	Asian	UCEC	NA	0.849	.
8:90983510-G/T	*NBN*	8:90983510	AA/B	BRCA	NA	0.617	.
8:90983510-G/T	*NBN*	8:90983510	White	BRCA	NA	0.617	.
8:90993081-C/A	*NBN*	8:90993081	White	UCEC	NA	0.581	.
8:90993671-C/A	*NBN*	8:90993671	Asian	STAD	NA	0.351	.
8:90993671-C/A	*NBN*	8:90993671	White	STAD	NA	0.351	.
8:90993708-G/T	*NBN*	8:90993708	White	UCEC	NA	0.866	.
8:90994993-C/T	*NBN*	8:90994993	White	COAD	rs759146120	0.449	.
8:95390494-T/G	*RAD54B*	8:95390494	Asian	UCEC	rs777020825	0.544	.

8:95392443-C/G	*RAD54B*	8:95392443	White	BRCA	NA	0.979	.
8:95392443-C/G	*RAD54B*	8:95392443	AA/B	BRCA	NA	0.979	.
8:95404031-G/T	*RAD54B*	8:95404031	White	COAD	NA	0.772	.
8:95404048-T/C	*RAD54B*	8:95404048	AA/B	BRCA	NA	0.763	.
8:95404048-T/C	*RAD54B*	8:95404048	White	BRCA	NA	0.763	.
8:95412652-C/T	*RAD54B*	8:95412652	White	ESCA	NA	0.935	.
8:95412652-C/T	*RAD54B*	8:95412652	Asian	ESCA	NA	0.935	.
8:95416359-C/T	*RAD54B*	8:95416359	Asian	BRCA	rs1032740646	0.948	.
8:95416359-C/T	*RAD54B*	8:95416359	White	UCEC	rs1032740646	0.948	.
8:95416359-C/T	*RAD54B*	8:95416359	White	BRCA	rs1032740646	0.948	.
9:110091866-T/C	*RAD23B*	9:110091866	Asian	UCEC	NA	0.627	.
9:115449873-T/A	*INIP*	9:115449873	White	KIRC	NA	0.415	.
9:115449873-T/A	*INIP*	9:115449873	White	KIRC	NA	0.716	.
9:115449873-T/A	*INIP*	9:115449873	AA/B	KIRC	NA	0.415	.
9:115449873-T/A	*INIP*	9:115449873	AA/B	KIRC	NA	0.716	.
9:115449874-C/G	*INIP*	9:115449874	White	KIRC	NA	0.328	.
9:115449874-C/G	*INIP*	9:115449874	White	KIRC	NA	0.637	.
9:115449874-C/G	*INIP*	9:115449874	AA/B	KIRC	NA	0.328	.
9:115449874-C/G	*INIP*	9:115449874	AA/B	KIRC	NA	0.637	.
9:131048293-G/T	*SWI5*	9:131048293	White	UCEC	NA	0.269	.
9:131048293-G/T	*SWI5*	9:131048293	White	UCEC	NA	0.575	.
9:131048293-G/T	*SWI5*	9:131048293	White	UCEC	NA	0.55	.
9:133729582-G/A	*ABL1*	9:133729582	White	BRCA	NA	0.536	.

9:133729582-G/A	*ABL1*	9:133729582	AA/B	BRCA	NA	0.536	.
9:133729585-T/G	*ABL1*	9:133729585	Asian	UCEC	NA	0.708	.
9:133738166-A/G	*ABL1*	9:133738166	White	STAD	NA	0.724	.
9:133738315-C/T	*ABL1*	9:133738315	AA/B	COAD	NA	0.672	.
9:133738315-C/T	*ABL1*	9:133738315	AA/B	COAD	NA	0.258	.
9:133738315-C/T	*ABL1*	9:133738315	White	COAD	NA	0.672	.
9:133738315-C/T	*ABL1*	9:133738315	White	COAD	NA	0.258	.
9:133748310-T/C	*ABL1*	9:133748310	White	COAD	NA	0.794	.
9:133748310-T/C	*ABL1*	9:133748310	AA/B	COAD	NA	0.794	.
9:133753820-G/T	*ABL1*	9:133753820	AA/B	LUAD	NA	0.841	.
9:133753820-G/T	*ABL1*	9:133753820	White	LUAD	NA	0.841	.
9:32973624-C/G	*APTX*	9:32973624	AA/B	LUAD	NA	0.804	.
9:32973624-C/G	*APTX*	9:32973624	White	LUAD	NA	0.804	.
9:32984659-C/T	*APTX*	9:32984659	Asian	UCEC	rs142133683	0.732	.
9:32985979-G/T	*APTX*	9:32985979	White	COAD	NA	0.836	.
9:32988085-T/G	*APTX*	9:32988085	White	COAD	NA	0.14	.
9:35076994-C/A	*FANCG*	9:35076994	White	UCEC	NA	0.11	.
9:35077299-A/G	*FANCG*	9:35077299	AA/B	COAD	rs776489249	0.722	.
9:35077299-A/G	*FANCG*	9:35077299	White	COAD	rs776489249	0.722	.
9:72895751-G/T	*SMC5*	9:72895751	Asian	STAD	NA	0.665	.
9:72895751-G/T	*SMC5*	9:72895751	White	STAD	NA	0.665	.
9:72915033-C/T	*SMC5*	9:72915033	White	UCEC	rs372120847	0.193	.
9:72962880-A/G	*SMC5*	9:72962880	Asian	ESCA	NA	0.36	.

9:72962880-A/G	*SMC5*	9:72962880	White	ESCA	NA	0.36	.
9:72962937-C/T	*SMC5*	9:72962937	White	STAD	NA	0.681	.
9:72965340-A/G	*SMC5*	9:72965340	White	COAD	NA	0.545	.
9:72967215-C/T	*SMC5*	9:72967215	White	LUSC	rs755324108	0.241	.
9:72967215-C/T	*SMC5*	9:72967215	AA/B	LUSC	rs755324108	0.241	.
9:75778399-G/A	*ANXA1*	9:75778399	White	STAD	rs1225300286	0.507	.
9:75778399-G/A	*ANXA1*	9:75778399	Asian	STAD	rs1225300286	0.507	.
9:75778440-G/A	*ANXA1*	9:75778440	White	COAD	NA	0.477	.
9:75778440-G/A	*ANXA1*	9:75778440	AA/B	COAD	NA	0.477	.
9:75781108-G/A	*ANXA1*	9:75781108	Asian	STAD	rs1369672677	0.371	.
9:75781108-G/A	*ANXA1*	9:75781108	White	STAD	rs1369672677	0.371	.
9:75782441-C/A	*ANXA1*	9:75782441	White	COAD	NA	0.329	.
9:75782463-G/A	*ANXA1*	9:75782463	White	UCEC	NA	0.413	.
9:75783994-G/A	*ANXA1*	9:75783994	White	KIRC	rs776755767	0.569	.
9:75783994-G/A	*ANXA1*	9:75783994	AA/B	KIRC	rs776755767	0.569	.
9:86616520-C/A	*RMI1*	9:86616520	Asian	UCEC	NA	0.208	.
9:86616989-C/T	*RMI1*	9:86616989	White	UCEC	NA	0.166	.
9:86617663-T/G	*RMI1*	9:86617663	White	COAD	NA	0.179	.
9:92220638-A/G	*GADD45G*	9:92220638	AA/B	LUAD	NA	0.749	.
9:92220638-A/G	*GADD45G*	9:92220638	White	LUAD	NA	0.749	.
9:92220676-T/G	*GADD45G*	9:92220676	White	COAD	NA	0.621	.
9:92220779-A/C	*GADD45G*	9:92220779	AA/B	LIHC	NA	0.312	.
9:97912234-G/T	*FANCC*	9:97912234	Asian	UCEC	NA	0.348	.

9:98009731-G/T	*FANCC*	9:98009731	Asian	UCEC	rs138722298	0.34	.
9:99285665-G/A	*CDC14B*	9:99285665	White	STAD	rs773237782	0.187	.
9:99296376-A/C	*CDC14B*	9:99296376	White	UCEC	NA	0.607	.
9:99296376-A/C	*CDC14B*	9:99296376	White	UCEC	NA	0.923	.
9:99327763-G/A	*CDC14B*	9:99327763	White	UCEC	rs373460925	0.54	.
9:99327763-G/A	*CDC14B*	9:99327763	White	UCEC	rs373460925	0.222	.
X:118708874-G/T	*UBE2A*	X:118708874	AA/B	LUAD	NA	0.812	.
X:118708874-G/T	*UBE2A*	X:118708874	White	LUAD	NA	0.812	.
X:118717161-A/C	*UBE2A*	X:118717161	AA/B	KIRC	NA	0.147	.
X:118717161-A/C	*UBE2A*	X:118717161	White	KIRC	NA	0.147	.
X:139865818-A/C	*CDR1*	X:139865818	Asian	STAD	NA	0.054	.
X:139865818-A/C	*CDR1*	X:139865818	White	STAD	NA	0.054	.
X:139865900-C/A	*CDR1*	X:139865900	Asian	UCEC	NA	0.124	.
X:14868746-G/C	*FANCB*	X:14868746	AA/B	BRCA	NA	0.171	.
X:14868746-G/C	*FANCB*	X:14868746	White	BRCA	NA	0.171	.
X:14871187-C/A	*FANCB*	X:14871187	Asian	UCEC	rs1354970109	0.067	.
X:14882881-T/A	*FANCB*	X:14882881	White	LUAD	NA	0.077	.
X:14882881-T/A	*FANCB*	X:14882881	AA/B	LUAD	NA	0.077	.
X:151998193-G/T	*CETN2*	X:151998193	Asian	UCEC	NA	0.503	.
X:152710700-G/C	*TREX2*	X:152710700	AA/B	LUAD	NA	0.412	.
X:152710700-G/C	*TREX2*	X:152710700	White	LUAD	NA	0.412	.
X:152710773-G/A	*TREX2*	X:152710773	White	LUSC	NA	0.154	.
X:152710773-G/A	*TREX2*	X:152710773	AA/B	LUSC	NA	0.154	.

X:152710780-G/A	*TREX2*	X:152710780	White	STAD	rs782056145	0.33	.
X:152710780-G/A	*TREX2*	X:152710780	Asian	STAD	rs782056145	0.33	.
X:152710795-G/T	*TREX2*	X:152710795	Asian	STAD	NA	0.18	.
X:152710795-G/T	*TREX2*	X:152710795	White	STAD	NA	0.18	.
X:154317544-G/A	*BRCC3*	X:154317544	Asian	BRCA	NA	0.335	.
X:154317544-G/A	*BRCC3*	X:154317544	White	BRCA	NA	0.335	.
X:154319106-G/T	*BRCC3*	X:154319106	Asian	UCEC	X:154319106_G/ T	0.093	.
X:154348284-G/T	*BRCC3*	X:154348284	Asian	UCEC	NA	0.162	.
X:24721417-C/T	*POLA1*	X:24721417	White	LUSC	rs747452548	0.282	.
X:24721417-C/T	*POLA1*	X:24721417	AA/B	LUSC	rs747452548	0.282	.
X:24732774-G/T	*POLA1*	X:24732774	Asian	UCEC	NA	0.155	.
X:24741356-G/A	*POLA1*	X:24741356	Asian	UCEC	rs751442544	0.517	.
X:24744109-G/T	*POLA1*	X:24744109	White	UCEC	NA	0.317	.
X:24746001-T/C	*POLA1*	X:24746001	Asian	UCEC	NA	0.26	.
X:24755801-A/C	*POLA1*	X:24755801	White	COAD	NA	0.262	.
X:24760155-G/A	*POLA1*	X:24760155	White	UCEC	NA	0.694	.
X:24760174-C/T	*POLA1*	X:24760174	Asian	BRCA	NA	0.379	.
X:24760174-C/T	*POLA1*	X:24760174	White	BRCA	NA	0.379	.
X:24761441-A/G	*POLA1*	X:24761441	Asian	UCEC	NA	0.424	.
X:24767001-G/T	*POLA1*	X:24767001	White	UCEC	NA	0.675	.
X:24906128-G/T	*POLA1*	X:24906128	AA/B	LUSC	NA	0.645	.
X:24906128-G/T	*POLA1*	X:24906128	White	LUSC	NA	0.645	.

X:47062354-A/G	*UBA1*	X:47062354	Asian	UCEC	NA	0.63	.
X:47069355-C/T	*UBA1*	X:47069355	White	UCEC	NA	0.205	.
X:47070254-G/A	*UBA1*	X:47070254	White	LUAD	NA	0.724	.
X:47070254-G/A	*UBA1*	X:47070254	AA/B	LUAD	NA	0.724	.
X:47072222-G/A	*UBA1*	X:47072222	White	UCEC	NA	0.73	.
X:47072254-C/T	*UBA1*	X:47072254	White	UCEC	rs782524260	0.501	.
X:47072566-G/A	*UBA1*	X:47072566	White	STAD	X:47072566_G/A	0.272	.
X:47074246-G/T	*UBA1*	X:47074246	AA/B	KIRP	NA	0.192	.
X:47074246-G/T	*UBA1*	X:47074246	White	KIRP	NA	0.192	.
X:55028033-G/T	*APEX2*	X:55028033	AA/B	SARC	NA	0.475	.
X:55028033-G/T	*APEX2*	X:55028033	White	SARC	NA	0.475	.
X:55028042-G/A	*APEX2*	X:55028042	White	STAD	rs374939146	0.651	.
X:55028042-G/A	*APEX2*	X:55028042	Asian	STAD	rs374939146	0.651	.
X:55028732-A/G	*APEX2*	X:55028732	Asian	UCEC	NA	0.87	.
X:55029522-G/T	*APEX2*	X:55029522	Asian	BRCA	NA	0.223	.
X:55029522-G/T	*APEX2*	X:55029522	White	BRCA	NA	0.223	.
X:69871378-C/T	*TEX11*	X:69871378	Asian	ESCA	NA	0.242	.
X:69871378-C/T	*TEX11*	X:69871378	White	ESCA	NA	0.242	.
X:69902665-C/A	*TEX11*	X:69902665	AA/B	KIRP	NA	0.346	.
X:69902665-C/A	*TEX11*	X:69902665	White	KIRP	NA	0.346	.
X:70026567-G/T	*TEX11*	X:70026567	Asian	UCEC	rs775355719	0.218	.
X:70073148-C/G	*TEX11*	X:70073148	White	LUAD	NA	0.12	.

X:70073148-C/G	*TEX11*	X:70073148	AA/B	LUAD	NA	0.12	.
X:96139695-G/T	*RPA4*	X:96139695	AA/B	BRCA	NA	0.536	.
X:96139695-G/T	*RPA4*	X:96139695	White	BRCA	NA	0.536	.
X:96140055-A/G	*RPA4*	X:96140055	White	ESCA	NA	0.466	.
X:96140055-A/G	*RPA4*	X:96140055	Asian	ESCA	NA	0.466	.
